# Metabolomic signatures reveal an association between healthy dietary patterns and brain aging

**DOI:** 10.1016/j.jnha.2026.100806

**Published:** 2026-02-19

**Authors:** Lingyuan Hu, Zhuotong Wang, Aomiao Chen, Geningyue Wang, Xinran Xie, Qiuyu He, Yu Wang, Huali Shi, Zongji Zheng, Yijie Jia

**Affiliations:** a Department of Endocrinology and Metabolism, Nanfang Hospital, Southern Medical University, Guangzhou 510515, China; b School of Stomatology, Southern Medical University, Guangzhou 510515, China; c The First School of Clinical Medicine, Southern Medical University, Guangzhou 510515, China

**Keywords:** Metabolomics, Brain age, Dietary pattern, AHEI-2010, DASH diet

## Abstract

**Objectives:**

The optimal dietary pattern of brain age and related diseases remains unclear, and the relationship between dietary metabolomic signature and these conditions is still poorly understood.

**Design, setting, and participants:**

This cohort study included 13,691 participants from the UK Biobank (53.67% female, mean age 54.9 ± 7.5 years), we investigated the relationship between five healthy dietary patterns and brain age gap (BAG). Metabolomic signatures were constructed using a LASSO model, and multivariable linear regression was applied to examine the relationship between metabolomic signatures and brain age.

**Results:**

Higher AHEI-2010 and DASH scores were associated with reduced BAG. Specifically, higher DASH scores reduced BAG in obese populations. Metabolomic signatures accounted for 30.43% and 35.47% of the associations between dietary patterns and BAG, respectively, and were themselves significantly correlated with BAG.

**Conclusion:**

AHEI-2010/DASH diets and plasma metabolites are associated with brain aging, offering a metabolomic basis for personalized dietary interventions.

## 1 Introduction

With the accelerated aging of the global population, maintaining brain health and delaying the onset of neurodegenerative diseases have become major public health challenges. Recent advances in neuroimaging technologies have emerged as a novel biomarker for studying brain aging: brain age. The discrepancy between predicted brain age (derived via structural MRI) and chronological age is termed the Brain Age Gap (BAG) [1]. A positive BAG value indicates accelerated brain aging, and is closely associated with progression of neurodegenerative diseases, including Alzheimer’s disease, Parkinson’s disease, multiple sclerosis, mild cognitive impairment, mood disorders, epilepsy, and schizophrenia [[2], [3], [4], [5]]. Furthermore, elevated BAG has been significantly linked to elevated risks of chronic diseases and mortality [6,7].

Lifestyle interventions, such as physical exercise and smoking cessation, are associated with slower biological aging processes [[8], [9], [10]]. Given its modifiable nature, diet has emerged as a critical and feasible strategy for promoting brain health. While studies on single nutrients have shown associations with reduced risks of chronic diseases, such research fails to fully capture the complexity of real-world dietary patterns. In contrast, healthy dietary patterns—such as the Alternative Healthy Eating Index (AHEI-2010), Dietary Approaches to Stop Hypertension (DASH), and Mediterranean diets (MED)—integrate diverse food combinations and nutrient synergies, and are associated with broader benefits for brain health. Observational studies have consistently linked these dietary patterns to enhanced brain structural integrity and delayed aging [[11], [12], [13], [14], [15], [16]]; however, the ideal dietary pattern for brain health remains debated, and the underlying mechanisms are not fully understood.

Metabolomics, a high-throughput analytical technology, enables systematic profiling of individual metabolic patterns and provides objective evidence for diet-brain associations. Although metabolomics has been extensively applied to study lifestyle factors, metabolic health, and cardiovascular diseases, its application in the field of brain aging remains limited [[17], [18], [19]]. The associations between diet-pattern-related metabolomic signatures and brain age gap have not been systematically explored to date, and the underlying links between diet, metabolism, and brain aging require further investigation.

To address these issues, this study utilizes neuroimaging and dietary data from the UK Biobank (UKB) to: (1) Investigate the hypothesized inverse associations between five healthy dietary patterns and BAG; (2) Elucidate the metabolomic signatures of these dietary patterns and analyze their correlations with brain age; and (3) validate whether metabolomic signatures mediate the inverse relationship between dietary patterns and brain aging.

By integrating neuroimaging, dietary assessment, and metabolomic analysis, this study aims to deepen the understanding of the links underlying dietary strategies to delay brain aging and provide actionable biomarkers and scientific evidence for personalized brain health interventions.

## 2 Materials and methods

### 2.1 Study population

This study is based on data from the UK Biobank, including a total of 13,691 participants. UK Biobank recruited over 500,000 participants aged 40–69 from 22 regions across the United Kingdom and collected detailed personal health information, including lifestyle and health data as well as biological sample and brain MRI. We excluded participants with missing baseline data on dietary intake, plasma metabolomics, or brain MRI (Supplementary Fig. S1).

### 2.2 Brain age calculation based on MRI

Based on brain magnetic resonance imaging (MRI) data from the UKB, this study constructed a brain age prediction model through rigorous sample selection. First, individuals were excluded if they had missing MRI data, self-reported neurological disorders, or long-term diseases, disabilities, frailty (field ID: 2188), or self-rated their health as fair or poor (field ID: 2178). The final dataset included participants meeting all inclusion criteria (N = 25,639). The model was built using 285 MRI-derived variables previously validated to correlate significantly with brain age (including brain region volumes, cortical thickness, and white matter integrity) [20]. Elastic Net Regression was employed as the prediction algorithm, and Bayesian Optimization was applied to optimize hyperparameters and enhance model generalization. Data were randomly split into training (80%) and testing (20%) sets. To address systematic biases where brain age is overestimated in younger individuals and underestimated in older individuals, we calibrated brain age using the slope and intercept derived from the training set predictions, thereby eliminating age-related systematic deviations. Brain age was predicted using the model and calculated based on the exact chronological age (in years) derived from participants' birth year/month to the MRI scan date. Brain Age Gap (BAG) was defined as the difference between predicted brain age and chronological age at MRI (BAG = Brain Age − Chronological Age). A positive BAG indicates accelerated brain aging (poorer health status), while a negative BAG suggests delayed brain aging (healthier status) (Supplementary Table S1).

### 2.3 Assessment of dietary pattern score

Based on data collected from the UKB between 2009 and 2012, participants completed the Oxford WebQ dietary assessment questionnaire at five different time points, all of which preceded the collection of MRI data in 2014. This questionnaire covered the intake frequency and portion sizes of 206 food items and 32 beverages, and detailed information regarding these aspects is available on the UKB website. For each participant, we calculated scores for five dietary pattern indices by averaging all available 24-h dietary recall data: the Alternative Healthy Eating Index (AHEI-2010), the Dietary Approaches to Stop Hypertension (DASH) diet, the energy-adjusted Dietary Inflammatory Index (e-DII), the Mediterranean Diet (MED), and the Mediterranean-DASH Intervention for Neurodegenerative Delay (MIND) diet. Except for e-DII, the scores for the other dietary patterns were standardized using the energy residual method, adjusting total energy intake to a uniform baseline of 2000 kcal to eliminate confounding effects of total energy intake on dietary quality scores, ensuring robustness of the results.

#### 2.3.1 Alternative Healthy Eating Index (AHEI-2010)

The AHEI-2010 (Alternative Healthy Eating Index-2010), developed by Harvard University based on U.S. dietary guidelines, is a dietary scoring system designed to assess the relationship between dietary patterns and the risk of chronic diseases, providing guidance for healthy eating choices [21]. It includes 11 categories of dietary intake: vegetables, fruits (excluding fruit juice), red and processed meats, nuts and legumes, sugary drinks, whole grains, trans fats, long-chain (n-3) fats, polyunsaturated fatty acids (PUFA), sodium, and alcohol consumption. Each category is scored from 0 to 10, with a total score ranging from 0 to 110. A higher score reflects greater adherence to the AHEI-2010 dietary pattern.

#### 2.3.2 Dietary Approaches to Stop Hypertension (DASH) score

The DASH diet aims to reduce hypertension and cardiovascular disease risk. In our study, the DASH score was calculated based on 8 dietary components: vegetables, fruits, red and processed meats, nuts and legumes, whole grains, low-fat foods, sugary drinks, and sodium. Each component is scored from 0 to 5 points, with a total score range of 0 to 40. A higher total score reflects greater adherence to the DASH diet.

#### 2.3.3 Energy-adjusted Dietary Inflammatory Index (e-DII)

The Dietary Inflammatory Index (DII) was initially developed in 2009 by public health expert Philip P Cavicchia, to quantify the pro-inflammatory or anti-inflammatory potential of dietary components, thereby reflecting an individual's dietary inflammatory potential [22]. The e-DII is an improved version of the DII, which adjusts for energy intake to eliminate the confounding effects of total energy consumption on inflammatory scores. According to prior research, the calculation of the e-DII score incorporates 32 nutrients, including alcohol, vitamins, fatty acids, cholesterol, dietary fiber, and their compositions, with dietary components standardized to intake per 1000 Kcal of energy [23]. A higher e-DII score indicates a greater pro-inflammatory potential of the diet.

#### 2.3.4 Mediterranean Diet (MED) score

The PREDIMED study developed a 14-point scoring system to assess adherence to the Mediterranean diet, which has been validated in diverse populations [24]. In our study, we adopted a previously used 13-point scoring system in the UK Biobank (UKB) cohort to evaluate the population's adherence to the Mediterranean diet [25]. This scoring system includes 13 food categories: vegetables, fruits, olive oil, red and processed meats, poultry, animal fats, sugary drinks, wine, legumes, nuts, seafood, sweets and desserts, and Sofrito. For each food category, a score of 1 is assigned if consumption meets or exceeds a predefined threshold, and 0 otherwise. Higher total scores reflect greater adherence to the Mediterranean diet.

#### 2.3.5 Mediterranean-DASH Intervention for Neurodegenerative Delay (MIND) diet score

The MIND diet, proposed by Martha Clare Morris, combines elements of the MED and DASH diets to capture food components with neuroprotective effects and to help delay age-related cognitive decline [26]. The calculation of the MIND diet score in the UK Biobank (UKB) has been previously reported [23], including 15 dietary categories: green leafy vegetables, other vegetables, berries, olive oil, red and processed meat, poultry, butter and margarine, cheese (not low-fat), wine, legumes, nuts, seafood, sweets/desserts/sugary drinks, whole grains, and fried foods. Each category is scored 0–1 based on intake frequency, with the total diet score ranging from 0 to 15. A higher score reflects greater adherence to the MIND diet.

### 2.4 Statistical analysis

In this study, dietary pattern scores were categorized into low, medium, and high tertiles. Continuous variables were described as mean ± standard deviation (Mean ± SD), while categorical variables were presented as frequencies and percentages (n, %). Missing covariates were imputed using multiple imputation by chained equations (MICE package) with five imputations performed to enhance model stability. The covariates with missing data (all with a missing rate of <10%) and their corresponding missing rates are detailed in Supplementary Table S2. Metabolite levels were log-transformed using natural log and analyzed in two steps to construct metabolomic signatures of dietary patterns: first, multivariable-adjusted linear regression was used to assess associations between individual metabolites and dietary pattern scores, with a Bonferroni-corrected P-value threshold of 0.05/251 applied; second, significant metabolites were selected as representative biomarkers via LASSO regression (10-fold cross-validation) to construct the metabolomic signature (Supplementary Table S3). Multivariable linear regression models were used in stratified analyses to evaluate associations between dietary pattern scores, metabolomic signatures, and BAG. Model 1 included no additional covariates. Model 2 adjusted for age, sex (male/female),race and ethnicity (white, Asian, Black, other race), Townsend deprivation index, smoking status (yes/no), alcohol consumption (yes/no), physical activity level (inactive, insufficient, sufficient), BMI category (<25, 25–30, >30), education level (college or above; A/AS levels or equivalent or O levels/GCSE or CSE or equivalent; NVQ or HND or HNC or equivalent or other professional qualifications, other). employment status (employed/unemployed/retired); and Standard PRS for AD. Model 3 further adjusted for total energy intake. Subgroup analyses were stratified by age, sex, BMI, smoking status, alcohol consumption, and chronic disease status. Sensitivity analyses additionally adjusted for diabetes, cardiovascular disease, and lipid-lowering medication use, and included individuals with complete metabolomic data but missing dietary data to assess independent associations between the metabolomic signature and BAG. All analyses were conducted via R version 4.3.0.

## 3 Results

### 3.1 Description of study populations

This study included 13,691 participants with metabolomic data (Females: 53.67%, mean age 54.9 ± 7.5). Baseline characteristics were stratified by tertiles of healthy dietary patterns as presented in Table 1 and Supplementary Table S4-S8. Except for e-DII, participants with higher healthy diet scores were more frequently female, non-working, had higher educational attainment, were less likely to be current drinkers, and exhibited lower BMI, smaller waist circumference, lower Townsend deprivation index scores. Additionally, those adhering to the AHEI-2010 and DASH dietary patterns were more likely to exhibit negative brain age.

**Table 1:** Baseline characteristics of participants across tertiles of the AHEI-2010 diet score.

Characteristics	Low (N = 4690)	Medium (N = 4516)	High (N = 4485)	p
Age, Mean (SD)	53.9 ± 7.6	55.0 ± 7.4	55.8 ± 7.4	<0.001
Sex, N (%)				<0.001
Female	2156 (46%)	2510 (55.6%)	2682 (59.8%)	
Male	2534 (54%)	2006 (44.4%)	1803 (40.2%)	
Race and ethnicity, N (%)				0.140
White	4590 (97.9%)	4415 (97.8%)	4353 (97.1%)	
Black	43 (0.9%)	43 (1%)	64 (1.4%)	
Asian	22 (0.5%)	22 (0.5%)	21 (0.5%)	
Other Race	35 (0.7%)	36 (0.8%)	47 (1%)	
Employment, N (%)				<0.001
Unemployed	209 (4.5%)	220 (4.9%)	242 (5.4%)	
Employed	2444 (52.1%)	2151 (47.6%)	1954 (43.6%)	
Retired	2037 (43.4%)	2145 (47.5%)	2289 (51%)	
Educational attainment, N (%)				<0.001
College or above	2061 (43.9%)	2318 (51.3%)	2476 (55.2%)	
A/AS levels or equivalent or O levels/GCSE or CSE or equivalent	1673 (35.7%)	1418 (31.4%)	1323 (29.5%)	
NVQ or HND or HNC or equivalent or other professional qualifications	703 (15%)	542 (12%)	498 (11.1%)	
Other	253 (5.4%)	238 (5.3%)	188 (4.2%)	
Alcohol use, N (%)				0.165
Yes	4502 (96%)	4333 (95.9%)	4273 (95.3%)	
No	188 (4%)	183 (4.1%)	212 (4.7%)	
Smoking, N (%)				<0.001
Yes	352 (7.5%)	213 (4.7%)	200 (4.5%)	
No	4338 (92.5%)	4303 (95.3%)	4285 (95.5%)	
BMI, N (%)				<0.001
<25	1614 (34.4%)	1873 (41.5%)	2223 (49.6%)	
25 to 30	2113 (45.1%)	1933 (42.8%)	1707 (38.1%)	
≥30	963 (20.5%)	710 (15.7%)	555 (12.4%)	
Waist, Mean (SD)	89.8 ± 12.6	87.1 ± 12.4	84.9 ± 11.8	<0.001
LTPA, N (%)				<0.001
None	3182 (67.8%)	3205 (71%)	3303 (73.6%)	
Inactive	1451 (30.9%)	1256 (27.8%)	1132 (25.2%)	
Active	57 (1.2%)	55 (1.2%)	50 (1.1%)	
Townsend deprivation index, Mean (SD)	−1.9 ± 2.7	−2.1 ± 2.6	−2.1 ± 2.6	<0.001
Standard PRS for AD, Mean (SD)	0.0 ± 1.0	0.0 ± 1.0	0.1 ± 1.0	0.018
BAG, Mean (SD)	0.2 ± 4.9	−0.4 ± 4.9	−0.5 ± 4.8	<0.001

LTPA: leisure time physical activity, "None" represents 0 min/wk of moderate-to-vigorous intensity physical activity (MVPA); "Inactive" corresponds to >0 to ≤150 min/wk of MVPA; and "Active" denotes >150 min/wk of MVPA; BAG, brain age gap.

### 3.2 Association between dietary patterns and BAG

**Fig. 1:**
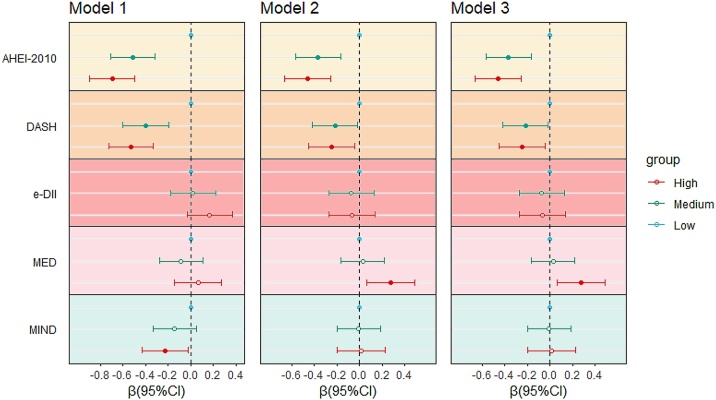
The association between dietary patterns and BAG (results from linear regression). Dietary pattern scores were categorized into low, medium, and high tertiles. Model 1 included no additional covariates. Model 2 adjusted for age, sex (male/female), race and ethnicity (white, Asian, Black, other race), Townsend deprivation index (continuous), smoking status (yes/no), alcohol consumption (yes/no), physical activity level (inactive, insufficient, sufficient), BMI category (<25, 25–30, >30), education level (college or above, A/AS levels or equivalent or O levels/GCSE or CSE or equivalent; NVQ or HND or HNC or equivalent or other professional qualifications; other), employment status (employed/unemployed/retired), and Standard PRS for AD. Model 3 further adjusted for total energy intake. Solid dots represent P < 0.05.

This study categorized dietary pattern scores into low, medium, and high tertiles. Higher dietary pattern scores were associated with reduced brain age (AHEI-2010, DASH and MIND) (Fig. 1 and Table 2). We observed that higher adherence to the AHEI-2010 was linked to negative BAG (β = −0.459 [−0.662, −0.255], P < 0.001). Each SD increase in the AHEI-2010 score was significantly associated with a reduction in BAG (β = −0.209 [−0.293, −0.125], P < 0.001). Compared to the lowest tertile, highest DASH scores were associated with lower BAG (β = −0.266 [−0.469, −0.062], P = 0.010), with each SD increase reducing BAG by approximately 14% (β = −0.139 [−0.224, −0.053], P = 0.001). The highest tertile of the MIND diet was associated with reduced BAG (β = −0.228 [−0.434, −0.021], P = 0.031), but significance was lost after adjustment for covariates. The e-DII and Mediterranean Diet (MED) showed nonsignificant associations with BAG. Surprisingly, after covariate adjustment, the highest MED tertile was positively associated with increased BAG (β = 0.259 [0.045, 0.473], P = 0.018), contradicting previous findings. This positive association disappeared after removing the wine component score from the MED score in sensitivity analyses (Supplementary Table S19). Subgroup analyses further revealed that in the MED highest tertile with high wine intake (≥7 wine drinks/day), the positive association between MED and BAG remained consistent in direction (β = 0.342 [−0.021, 0.704], P = 0.065), showing a marginally significant trend that may be limited by the small sample size of this subgroup. In detailed dietary component analyses, higher intake of fruits, whole grains, trans fatty acids, polyunsaturated fatty acids (PUFA), and low-fat dairy were associated with slowed brain aging and improved brain health (fruits: β = −0.151 [−0.232, −0.069]; grains: β = −0.137 [−0.219, −0.055]; trans fats: β = −0.115 [−0.197, −0.034]; PUFA: β = −0.133 [−0.215, −0.052]; low-fat dairy: β = −0.143 [−0.225, −0.061]). In contrast, higher consumption of red meat, sugar-sweetened beverages, and alcohol was associated with larger BAG, potentially harming brain health (red meat: β = 0.112 [0.030, 0.193]; sugary drinks: β = 0.082 [0.000, 0.163]; alcohol: β = 0.496 [0.413, 0.580]) (Supplementary Table S9).

**Table 2:** The association between dietary patterns and BAG (results from linear regression).

Dietary pattern	Model 1	P	Model 2	P	Model 3	P
**MED**						
Low	Reference	–	Reference	–	Reference	–
Medium	−0.088 (−0.279, 0.103)	0.367	0.027 (−0.164, 0.219)	0.779	0.019 (−0.173, 0.211)	0.842
High	0.063 (−0.146, 0.272)	0.555	0.276 (0.064, 0.488)	0.011	0.259 (0.045, 0.473)	0.018
P for trend		0.631		0.014		0.022
**DASH**						
Low	Reference	–	Reference	–	Reference	–
Medium	−0.398 (−0.598, −0.198)	<0.001	−0.217 (−0.419, −0.016)	0.035	−0.217 (−0.419, −0.016)	0.035
High	−0.529 (−0.725, −0.332)	<0.001	−0.250 (−0.452, −0.047)	0.016	−0.266 (−0.469, −0.062)	0.010
P for trend		<0.001		0.015		0.010
**MIND**						
Low	Reference	–	Reference	–	Reference	–
Medium	−0.143 (−0.336, 0.049)	0.144	−0.007 (−0.201, 0.186)	0.941	−0.009 (−0.203, 0.184)	0.924
High	−0.228 (−0.434, −0.021)	0.031	0.016 (−0.196, 0.229)	0.879	0.003 (−0.210, 0.216)	0.979
P for trend		0.028		0.888		0.985
**AHEI-2010**						
Low	Reference	–	Reference	–	Reference	–
Medium	−0.513 (−0.711, −0.314)	<0.001	−0.366 (−0.566, −0.167)	<0.001	−0.362 (−0.562, −0.163)	<0.001
High	−0.695 (−0.895, −0.496)	<0.001	−0.458 (−0.662, −0.255)	<0.001	−0.459 (−0.662, −0.255)	<0.001
P for trend		<0.001		<0.001		<0.001
**e-DII**						
Low	Reference	–	Reference	–	Reference	–
Medium	0.020 (−0.180, 0.220)	0.841	−0.074 (−0.274, 0.126)	0.468	−0.098 (−0.300, 0.103)	0.339
High	0.167 (−0.033, 0.366)	0.103	−0.071 (−0.275, 0.133)	0.496	−0.119 (−0.329, 0.092)	0.268
P for trend		0.103		0.495		0.268

Dietary pattern scores were categorized into low, medium, and high tertiles. Model 1 included no additional covariates. Model 2 adjusted for age, sex (male/female), race and ethnicity (white, Asian, Black, other race), Townsend deprivation index (continuous), smoking status (yes/no), alcohol consumption (yes/no), physical activity level (inactive, insufficient, sufficient), BMI category (<25, 25–30, >30), education level (college or above; A/AS levels or equivalent or O levels/GCSE or CSE or equivalent; NVQ or HND or HNC or equivalent or other professional qualifications; other), employment status (employed/unemployed/retired), and Standard PRS for AD. Model 3 further adjusted for total energy intake.

### 3.3 Metabolomic signatures for dietary patterns

**Fig. 2:**
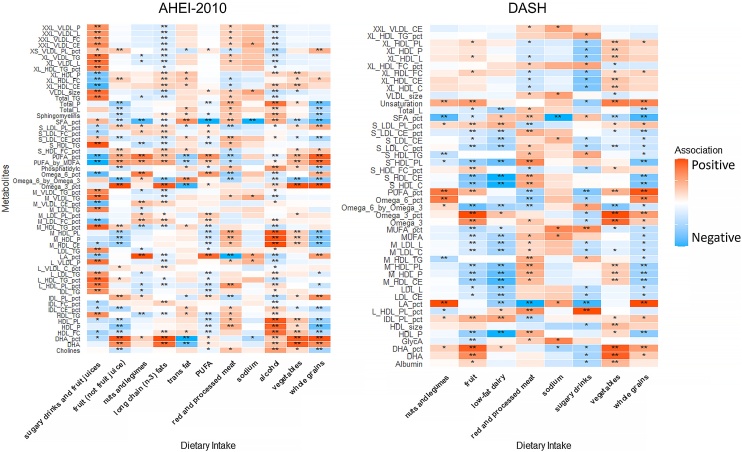
Association between diet components and individual metabolites in metabolomic signature. Red indicates positive correlations, blue indicates negative correlations, and the shading depth reflects the absolute value of the correlation coefficients in linear regression, with darker shades corresponding to larger values. Significance: * P < 0.05, ** P < 0.001 (For interpretation of the references to colour in this figure legend, the reader is referred to the web version of this article).

We further explored the relationships between metabolites and the AHEI-2010 and DASH scores. Metabolites significantly associated with dietary pattern scores were selected using Bonferroni-corrected P-values, and LASSO models were applied to construct metabolomic signatures for the AHEI-2010 and DASH patterns. The AHEI-2010 metabolomic signature incorporated 56 metabolites related to healthy dietary patterns, including triglycerides, fatty acids, lipoprotein-related components, phospholipids, and choline. The DASH metabolomic signature included 46 metabolites, which, in addition to lipid-related markers, also contained albumin and glycoprotein acetyls—markers of inflammation (Supplementary Tables S10 & S11). Within the AHEI-2010 signature, 20 metabolites were positively correlated with dietary scores, while 36 were negatively correlated; phospholipid concentrations and proportions showed significant positive correlations (Supplementary Table S10). For DASH, 19 metabolites were positively correlated and 27 were negatively correlated with the dietary score (Supplementary Table S11). The metabolomic signatures demonstrated strong associations with their respective dietary patterns (AHEI-2010: Pearson r = 0.327, R^2^ = 0.107, P < 0.001; DASH: Pearson r = 0.280, R² = 0.078, P < 0.001). Additionally, the AHEI-2010 and DASH metabolomic signatures were highly positively correlated (Pearson r = 0.905, P < 0.001), indicating that the two signatures capture similar metabolic pathways underlying healthy dietary patterns. We further examined correlations between metabolomic components and dietary component intake (Fig. 2). Most metabolites showed significant associations with dietary component intake, with many demonstrating positive correlations with alcohol consumption and negative correlations with whole grain intake (Supplementary Tables S12 and S13). A SD increase in the metabolomic signature was significantly associated with negative BAG values. For the AHEI-2010 score, a one-SD increase in the metabolomic signature was associated with lower BAG (β = −0.306 [−0.396, −0.216], P < 0.001), and similarly for DASH (β = −0.218 [−0.285, −0.150], P < 0.001). The metabolomic signatures demonstrated stronger associations with BAG compared to the dietary scores themselves (Table 3).

**Table 3:** Effect of a SD Increase in dietary patterns and metabolomic signatures on BAG.

Per-SD β(95%CI)	Model 1	Model 2	Model 3
AHEI-2010	−0.318 (−0.399, −0.236)	−0.209 (−0.293, −0.125)	−0.209 (−0.293, −0.125)
Metabolomic signature	−0.305 (−0.395, −0.215)	−0.305 (−0.395, −0.215)	−0.306 (−0.396, −0.216)
DASH	−0.263 (−0.345, −0.182)	−0.131 (−0.216, −0.046)	−0.139 (−0.224, −0.053)
Metabolomic signature	−0.455 (−0.536, −0.374)	−0.298 (−0.391, −0.205)	−0.218 (−0.285, −0.150)

Model 1 included no additional covariates. Model 2 adjusted for age, sex (male/female), race and ethnicity (white, Asian, Black, other race), Townsend deprivation index (continuous), smoking status (yes/no), alcohol consumption (yes/no), physical activity level (inactive, insufficient, sufficient), BMI category (<25, 25–30, >30), education level (college or above; A/AS levels or equivalent or O levels/GCSE or CSE or equivalent; NVQ or HND or HNC or equivalent or other professional qualifications; other), employment status (employed/unemployed/retired), and Standard PRS for AD. Model 3 further adjusted for total energy intake.

### 3.4 Mediation analysis

We further conducted causal mediation analysis to examine the associations between dietary patterns, metabolomic signatures, and BAG. In the AHEI-2010 model, the metabolomic signature accounted for 30.43% (95%CI: 0.168, 0.541) of the association between dietary scores and BAG. For DASH, the metabolomic signature accounted for 35.47% (95%CI: 0.188, 1.005) of the association between dietary scores and BAG (Supplementary Table S14).

### 3.5 Subgroup and sensitivity analyses

We further explored the associations between dietary patterns, metabolomic signatures, and BAG through stratified analysis by age, sex, BMI, alcohol consumption, cardiovascular disease, and diabetes (Supplementary Tables S15 & S16). We observed a significant interaction between BMI stratification and DASH (P for interaction = 0.012), where the association between DASH and BAG was only evident in individuals with BMI ≥ 30. In contrast, for AHEI-2010, the significant association between the diet score and BAG disappeared in the BMI ≥ 30 subgroup. This suggests that the DASH diet may confer greater benefits for brain health in obese individuals, while the AHEI-2010 appears to be more strongly associated with reduced brain aging in populations with lower BMI. When further adjusted for chronic diseases and lipid-lowering medication use, the results of sensitivity analyses remained consistent (Supplementary Table S17). In participants with metabolomic data but missing dietary data, the association between metabolomic signatures and BAG persisted (Supplementary Table S18).

## 4 Discussion

This study demonstrates that both the AHEI-2010 and DASH dietary patterns are significantly associated with a reduction in BAG, with AHEI-2010 exhibiting a stronger association. Additionally, we found that the AHEI-2010 dietary pattern is more conducive to promoting brain health in non-obese individuals, whereas the DASH dietary pattern is more beneficial for improving brain health in obese populations. The distinct metabolomic signatures associated with these dietary patterns show significant correlations with BAG and mediate 30.43% (AHEI-2010) and 35.47% (DASH) of the total effect, respectively.

Multiple studies have highlighted the close association between dietary patterns and brain health in recent years. Dietary patterns rich in polyphenols and plant-based components, such as the Mediterranean diet (MED), MIND diet, and AHEI-2010 diet, have been shown to reduce the risk of Alzheimer’s disease (AD) and cognitive decline by optimizing nutrient intake [12,27,28]. Conversely, pro-inflammatory diets high in red meat and ultra-processed foods are linked to reduced brain volume and cognitive dysfunction [[29], [30], [31]]. The association of alcohol consumption with brain health exhibits a dual-edged sword pattern: moderate red wine consumption, rich in polyphenols and ketone metabolites, may confer neuroprotective benefits, while excessive alcohol intake increases amyloid-β (Aβ) deposition and elevates AD risk [32]. Previous studies have reported inconsistent findings regarding the association between healthy dietary patterns and brain health: some studies report protective effects on cognitive function and brain disorder [33,34]. For instance, higher AHEI-2010 scores were associated with larger hippocampal volumes and higher hippocampal functional connectivity, suggesting a role in delaying age-related structural brain degeneration [16,35]. However, studies by Bernhard and Lizanne et al. reported no significant associations between healthy dietary patterns and cognitive decline or brain structural connectivity [36,37]. This study further validated the association between adherence to healthy dietary patterns like AHEI-2010 and DASH and structure-based brain age metrics through metabolomic profiling, thereby supporting the conclusion that these diets are linked to younger brain age.

Different dietary patterns are associated with brain health in distinct ways due to their unique nutritional compositions: the MIND diet specifically emphasizes berry consumption, which may exert antioxidant effects; the MED promotes olive oil intake, which is linked to lower lipid levels and less inflammation; the DASH diet is associated with less metabolomic dysfunction in relation to sodium restriction; in our study, we observed that higher AHEI-2010 scores were significantly associated with lower brain age. These observed benefits may relate to the diet’s comprehensive emphasis on plant-based foods and restriction of ultra-processed food—links aligned with associations between these dietary factors and improved cardiovascular health [38,39]. Notably, the DASH diet showed associations with enhanced brain health outcomes in obese individuals (BMI > 30), likely relating to its focus on lowering saturated fats and sodium. This is linked to less metabolic dysfunction, a factor associated with neurodegenerative changes in this population. In this study, higher MED scores were associated with accelerated brain aging, contrary to previous research findings. However, this unexpected positive association was no longer evident after excluding the alcohol component from the MED score. This result suggests that alcohol consumption, as a component of the standard MED dietary pattern, may counteract its potential neuroprotective effects in our study population. Although moderate alcohol consumption is a hallmark of the traditional MED diet, individual variations in drinking frequency or quantity might lead to excessive alcohol exposure in certain populations, thus offsetting the benefits of other healthy nutritional components of the diet. Further research is needed to clarify the potential threshold of alcohol intake associated with brain health in diverse populations. We did not observe an association between the e-DII diet and reduced brain aging. After adjusting for energy intake, no significant association was found between the MIND diet and BAG in our study population.

In our study, we identified 56 AHEI-2010-associated and 46 DASH-associated metabolites, predominantly involving lipid metabolic pathways. The AHEI-2010 score was positively correlated with smaller low-density lipoproteins (LDL), unsaturated fatty acids, and PUFAs, while negatively associated with larger lipoproteins, triglycerides, and choline. In contrast, the DASH pattern showed similar lipid-related associations but additionally exhibited positive correlations with albumin and negative correlations with glycoprotein acetylation markers. These metabolites are associated with neurofunctional processes, as well as with inflammation and oxidative stress. Omega-6 fatty acids are associated with various diseases, including stroke, Alzheimer's disease, and dementia [40,41]. Previous studies have highlighted that PUFAs are implicated in neuronal signaling, neuroinflammation, and cerebral glucose uptake, playing critical roles in maintaining brain function [42,43]. The metabolomic signatures of these dietary patterns reflect their distinct metabolic regulatory links: the AHEI-2010 metabolomic profile uniquely included choline-related markers, while the DASH signature additionally encompassed albumin and glycoprotein acetylation markers linked to inflammatory pathways. Choline can alleviate hippocampal pathological damage, significantly reduce inflammatory and oxidative stress biomarkers, and attenuate microglial activation in the hippocampus, thereby preserving synaptic plasticity [44]. Glycoprotein acetylation, conversely, is tied to low-grade inflammation, modulating microglial function and contributing to neuroinflammation, amyloid-β (Aβ) accumulation, and tau pathology [45]. Among nutrients, metabolites were most strongly positively correlated with alcohol consumption and negatively correlated with grain intake, consistent with prior research findings [46].

Diet plays a critical role in shaping individual metabolomic profiles. In our study, metabolomic signatures objectively reflect the association between diet and brain health. Even in populations with only metabolomic data available, these signatures remained significantly associated with BAG. Metabolomic signatures integrate genetic susceptibility and individual-specific factors, providing a comprehensive representation of biological interactions. Increasingly, metabolomic signatures are being used as predictive biomarkers for disease, revealing associations between specific metabolites or metabolic patterns and disease risk, thereby underscoring the potential of metabolomics in elucidating the impact of diet on health [47]. Compared to self-reported dietary scores, metabolomic-based dietary signatures offer a more objective and precise assessment of individual nutritional status. This approach enables a deeper understanding of the reasons behind individual differences in response to the same dietary interventions. Utilizing metabolomic signatures not only aids in identifying individuals who benefit most from specific dietary patterns but also provides a scientific foundation for developing personalized brain health management strategies. Such strategies could enhance the promotion of brain health and delay aging processes through targeted, evidence-based interventions.

This study provides new insights into identifying healthy dietary patterns that benefit to brain and elucidates the metabolomic signatures associated with these dietary patterns. A key strength of our research is the use of a large population sample and brain MRI data to evaluate the relationship between healthy dietary practices and brain age. However, our study has several limitations. First, dietary data in the UK Biobank relied on self-reported questionnaires, which may introduce measurement inaccuracies. Additionally, the generally healthier profile of UK Biobank participants may limit the generalizability of our findings to broader populations. While we extensively adjusted for multiple covariates in our analyses, residual confounding from unmeasured variables cannot be fully excluded. Moreover, the cross-sectional nature of our study design precludes the establishment of a causal temporal relationship between metabolomic signatures and BAG. Furthermore, there may be bidirectional relationships between diet and brain age, as older brain age could potentially impair an individual’s ability to adhere to healthy dietary practices. In addition, the limited range of metabolites measured in the UK Biobank platform might have omitted important metabolites, which could affect the robustness of our conclusions. Lastly, potential overfitting may exist during metabolomic signature construction, which could lead to the derived signature performing optimally only in the current dataset. Future research should further explore the complex associations between diet and brain age in more diverse populations and employ more comprehensive metabolomic profiling techniques to validate and expand upon our findings.

## 5 Conclusion

In conclusion, this study demonstrates that adherence to both the AHEI-2010 and DASH dietary patterns is significantly associated with negative BAG. Further analysis revealed distinct dietary effects based on obesity status: the AHEI-2010 was more effective in promoting brain health in non-obese individuals, while the DASH pattern showed greater benefits for improving brain health in obese populations. Metabolomic signatures unique to each dietary pattern were strongly correlated with BAG. This study demonstrates that dietary pattern-specific metabolomic signatures provide an objective molecular reflection of dietary intake relevant to healthy dietary patterns, offering a scientific foundation for developing personalized brain health strategies to promote brain health maintenance and delay the aging process.

## CRediT authorship contribution statement

L.H.: Conceptualization, Methodology, Software, Writing - Original Draft. Z.W.: Conceptualization, Software, Validation, Writing - Original Draft. A.C.: Validation, Investigation, Writing - Review & Editing. G.W.: Validation, Investigation, Writing - Review & Editing. X.X.: Validation, Investigation, Writing - Review & Editing. Q.H.: Validation, Investigation, Writing - Review & Editing. Y.W.: Validation, Investigation, Writing - Review & Editing. H.S.: Validation, Investigation, Writing - Review & Editing. Z.Z.: Conceptualization, Writing - Review & Editing, Supervision. Y.J.: Conceptualization, Writing - Review & Editing, Supervision.

## Ethics statement and consent for publication

UK Biobank was conducted according to the guidelines of the Declaration of Helsinki and had approval from the North West Multicentre Research Ethics Committee (REC reference: 21/NW/0157), and all participants provided written informed consent. No additional ethical approval was required. Not applicable to clinical trial number.

## Declaration of Generative AI and AI-assisted technologies in the writing process

Not applicable.

## Funding

This work was supported by the 10.13039/501100001809National Natural Science Foundation of China (82270862, 82370818); Guangdong Basic and Applied Basic Research Foundation (2024A1515012744, 2024A1515220024); the Guangzhou Science and Technology Project (2024A04J5098, 2025A04J3541); and the National Undergraduate Training Program for Innovation and Entrepreneurship, Southern Medical University (202512121246, 202312121031, S202312121167).

## Data availability statement

The data supporting the findings of this study are available from the UK Biobank, but access is subject to restrictions. Researchers may request access through the UK Biobank application process.

## Declaration of competing interest

All authors declare that they have no competing interests.

## References

[1] Cole J.H., Marioni R.E., Harris S.E., Deary I.J. (2019). Brain age and other bodily ‘ages’: implications for neuropsychiatry. Mol Psychiatry..

[2] Pontillo G., Prados F., Colman J., Kanber B., Abdel-Mannan O., Al-Araji S. (2024). Disentangling neurodegeneration from aging in multiple sclerosis using deep learning: the brain-predicted disease duration gap. Neurology..

[3] Sone D., Beheshti I. (2022). Neuroimaging-based brain age estimation: a promising personalized biomarker in neuropsychiatry. J Pers Med.

[4] Moguilner S., Baez S., Hernandez H., Migeot J., Legaz A., Gonzalez-Gomez R. (2024). Brain clocks capture diversity and disparities in aging and dementia across geographically diverse populations. Nat Med..

[5] Gaser C., Kalc P., Cole J.H. (2024). A perspective on brain-age estimation and its clinical promise. Nat Comput Sci.

[6] Huang H., Wang J., Dunk M.M., Guo J., Dove A., Ma J. (2024). Association of cardiovascular health with brain age estimated using machine learning methods in middle-aged and older adults. Neurology..

[7] Kahhale I., Puccetti N.A., Heller A.S., Hanson J.L. (2025). Probing connections between social connectedness, mortality risk, and brain age: a preregistered study. J Personality Social Psychol..

[8] Senff J., Tack R.W.P., Mallick A., Gutierrez-Martinez L., Duskin J., Kimball T.N. (2025). Modifiable risk factors for stroke, dementia and late-life depression: a systematic review and DALY-weighted risk factors for a composite outcome. J Neurol Neurosurg Psychiatry..

[9] Tari A.R., Walker T.L., Huuha A.M., Sando S.B., Wisloff U. (2025). Neuroprotective mechanisms of exercise and the importance of fitness for healthy brain ageing. Lancet (London, England)..

[10] Pradeepkiran J.A., Islam M.A., Sehar U., Reddy A.P., Vijayan M., Reddy P.H. (2025). Impact of diet and exercise on mitochondrial quality and mitophagy in Alzheimer’s disease. Ageing Res Rev.

[11] Arnoldy L., Gauci S., Young L.M., Macpherson H., Civier O., Scholey A. (2025). Assessing the association between the Mediterranean, DASH, and MIND dietary patterns, structural connectivity, and cognitive function. Br J Nutr.

[12] Gong Y., Chen H., Gu Y., Shen J., Shen T., Ding Y. (2025). Healthy dietary patterns in relation to cognitive performance and Alzheimer’s disease mortality. J Prev Alzheimers Dis.

[13] Morris M.C., Wang Y., Barnes L.L., Bennett D.A., Dawson-Hughes B., Booth S.L. (2018). Nutrients and bioactives in green leafy vegetables and cognitive decline: Prospective study. Neurology..

[14] van Soest A.P., Beers S., van de Rest O., de Groot L.C. (2024). The Mediterranean-dietary approaches to stop hypertension intervention for neurodegenerative delay (MIND) diet for the aging brain: a systematic review. Adv Nutr (Bethesda, Md)..

[15] Morris M.C., Tangney C.C., Wang Y., Sacks F.M., Bennett D.A., Aggarwal N.T. (2015). MIND diet associated with reduced incidence of Alzheimer’s disease. Alzheimers Dement.

[16] Jensen D.E.A., Ebmeier K.P., Akbaraly T., Jansen M.G., Singh-Manoux A., Kivimäki M. (2025). Association of diet and waist-to-hip ratio with brain connectivity and memory in aging. JAMA Network Open.

[17] Barovic M., Hahn J.J., Heinrich A., Adhikari T., Schwarz P., Mirtschink P. (2025). Proteomic and metabolomic signatures in prediabetes progressing to diabetes or reversing to normoglycemia within 1 year. Diabetes Care.

[18] Zhou Z., Wang L., Chen Y. (2025). Enhancing the study of air pollution, metabolomic signatures, and chronic respiratory disease risk: addressing dietary, noise, and exposure factors. Chest..

[19] Hang D., Zeleznik O.A., He X., Guasch-Ferre M., Jiang X., Li J. (2020). Metabolomic signatures of long-term coffee consumption and risk of type 2 diabetes in women. Diabetes Care.

[20] Dove A., Wang J., Huang H., Dunk M.M., Sakakibara S., Guitart-Masip M. (2024). Diabetes, prediabetes, and brain aging: the role of healthy lifestyle. Diabetes Care..

[21] Shivappa N., Hebert J.R., Kivimaki M., Akbaraly T. (2017). Alternative Healthy Eating Index 2010, Dietary Inflammatory Index and risk of mortality: results from the Whitehall II cohort study and meta-analysis of previous Dietary Inflammatory Index and mortality studies. Br J Nutr..

[22] Cavicchia P.P., Steck S.E., Hurley T.G., Hussey J.R., Ma Y., Ockene I.S. (2009). A new dietary inflammatory index predicts interval changes in serum high-sensitivity C-reactive protein. J Nutr.

[23] Zhu K., Li R., Yao P., Yu H., Pan A., Manson J.E. (2025). Proteomic signatures of healthy dietary patterns are associated with lower risks of major chronic diseases and mortality. Nat Food..

[24] Martínez-González M.Á, Corella D., Salas-Salvadó J., Ros E., Covas M.I., Fiol M. (2012). Cohort profile: design and methods of the PREDIMED study. Int J Epidemiol..

[25] Shannon O.M., Ranson J.M., Gregory S., Macpherson H., Milte C., Lentjes M. (2023). Mediterranean diet adherence is associated with lower dementia risk, independent of genetic predisposition: findings from the UK Biobank prospective cohort study. BMC Med..

[26] Morris M.C., Tangney C.C., Wang Y., Sacks F.M., Barnes L.L., Bennett D.A. (2015). MIND diet slows cognitive decline with aging. Alzheimers Dement..

[27] Joyce E.E., Yin W., Löf M., Wirdefeldt K., Sandin S., Fang F. (2025). Mediterranean dietary pattern and risk of neurodegenerative diseases in a cohort of Swedish women. NPJ Parkinsons Dis.

[28] Molero P., De Lorenzi F., Gędek A., Strater C., Popescu E., Ortuño F. (2025). Diet quality and depression risk: a systematic review and meta-analysis of prospective studies. J Affect Disord.

[29] Gomes Gonçalves N., Vidal Ferreira N., Khandpur N., Martinez Steele E., Bertazzi Levy R., Andrade Lotufo P. (2023). Association between consumption of ultraprocessed foods and cognitive decline. JAMA Neurol..

[30] Qi Z., Cao J., Liu J., Chen J., Chen S., Zhang L. (2025). Toxicological mechanisms of carbon polymers in accelerating cognitive decline in Alzheimer’s disease. J Adv Res.

[31] Bhave V.M., Oladele C.R., Ament Z., Kijpaisalratana N., Jones A.C., Couch C.A. (2024). Associations between ultra-processed food consumption and adverse brain health outcomes. Neurology..

[32] Drouka A., Ntetsika K.D., Brikou D., Mamalaki E., Ntanasi E., Chatzipanagiotou S. (2025). Associations of moderate alcohol intake with cerebrospinal fluid biomarkers of Alzheimer’s disease: data from the ALBION study. Eur J Nutr.

[33] Lu Z., Chen C., Zhang J., Wang X., Zhang D., Li S. (2022). The relationship between alternative healthy diet index and cognitive function in the older adults: the mediating effect of depressive symptoms. Nutrients..

[34] Cornelis M.C., Agarwal P., Holland T.M., van Dam R.M. (2022). MIND dietary pattern and its association with cognition and incident dementia in the UK Biobank. Nutrients..

[35] Akbaraly T., Sexton C., Zsoldos E., Mahmood A., Filippini N., Kerleau C. (2018). Association of long-term diet quality with hippocampal volume: longitudinal cohort study. Am J Med.

[36] Rotstein D.L., Cortese M., Fung T.T., Chitnis T., Ascherio A., Munger K.L. (2019). Diet quality and risk of multiple sclerosis in two cohorts of US women. Mult Scler (Houndmills, Basingstoke, England)..

[37] Haring B., Wu C., Mossavar-Rahmani Y., Snetselaar L., Brunner R., Wallace R.B. (2016). No association between dietary patterns and risk for cognitive decline in older women with 9-year follow-up: data from the women’s health initiative memory study. J Acad Nutr Diet..

[38] Akbaraly T.N., Shipley M.J., Ferrie J.E., Virtanen M., Lowe G., Hamer M. (2015). Long-term adherence to healthy dietary guidelines and chronic inflammation in the prospective Whitehall II study. Am J Med.

[39] Tessier A.J., Wang F., Korat A.A., Eliassen A.H., Chavarro J., Grodstein F. (2025). Optimal dietary patterns for healthy aging. Nat Med.

[40] (2024). Global, regional, and national burden of stroke and its risk factors, 1990-2021: a systematic analysis for the Global Burden of Disease Study 2021. Lancet Neurol..

[41] Dicks L.M.T. (2024). How important are fatty acids in human health and can they be used in treating diseases?. Gut Microbes.

[42] Tian J., Zhang Y., Zhao X. (2025). The effects and mechanisms of n-3 and n-6 polyunsaturated fatty acids in the central nervous system. Cell Mol Neurobiol.

[43] Janssen C.I., Kiliaan A.J. (2014). Long-chain polyunsaturated fatty acids (LCPUFA) from genesis to senescence: the influence of LCPUFA on neural development, aging, and neurodegeneration. Prog Lipid Res..

[44] Huang S.Y., Yang Z.J., Cheng J., Li H.Y., Chen S., Huang Z.H. (2025). Choline alleviates cognitive impairment in sleep-deprived young mice via reducing neuroinflammation and altering phospholipidomic profile. Redox Biol.

[45] Zwiep J.C., Milaneschi Y., Giltay E.J., Vinkers C.H., Penninx B., Lamers F. (2025). Depression with immuno-metabolic dysregulation: testing pragmatic criteria to stratify patients. Brain Behav Immun..

[46] Feng L., Milleson H.S., Ye Z., Canida T., Ke H., Liang M. (2024). Nongenetic and genetic factors associated with white matter brain aging: exposome-wide and genome-wide association study. Genes..

[47] Akbaraly T., Würtz P., Singh-Manoux A., Shipley M.J., Haapakoski R., Lehto M. (2018). Association of circulating metabolites with healthy diet and risk of cardiovascular disease: analysis of two cohort studies. Sci Rep.

