# Supplementary material for: Metabolomic signatures reveal an association between healthy dietary patterns and brain aging
Source: J Nutr Health Aging. 2026 Feb 19;30(4):100806. doi: 10.1016/j.jnha.2026.100806 (PMC12934320; doi:10.1016/j.jnha.2026.100806)
Supplement: Supplementary file 1 [file mmc1.docx]

Supplementary material

[Supplementary Figure 1 Participant flow diagram 2](#_Toc220523206)

[Supplementary Table 1 Key performance indicators of the brain age prediction model 3](#_Toc220523207)

[Supplementary Table 2 Missing rates of covariates undergoing imputation 4](#_Toc220523208)

[Supplementary Table 3 LASSO cross-validation metrics for dietary metabolomic signatures 4](#_Toc220523209)

[Supplementary Table 4 Mean and Standard Deviation of Dietary Scores 4](#_Toc220523210)

[Supplementary Table 5 Baseline characteristics of participants across tertiles of the DASH diet score 5](#_Toc220523211)

[Supplementary Table 6 Baseline characteristics of participants across tertiles of the e-DII diet score 7](#_Toc220523212)

[Supplementary Table 7 Baseline characteristics of participants across tertiles of the MED diet score 9](#_Toc220523213)

[Supplementary Table 8 Baseline characteristics of participants across tertiles of the MIND diet score 11](#_Toc220523214)

[Supplementary Table 9 Effect of a SD Increase in Dietary Component Intake on BAG 13](#_Toc220523215)

[Supplementary Table 10 Detailed information of metabolomic signature of AHEI-2010 in our study 14](#_Toc220523216)

[Supplementary Table 11 Detailed information of metabolomic signature of DASH in our study 16](#_Toc220523217)

[Supplementary Table 12 Association between AHEI diet components and individual metabolites in metabolomic signature 18](#_Toc220523218)

[Supplementary Table 13 Association between DASH diet components and individual metabolites in metabolomic signature 31](#_Toc220523219)

[Supplementary Table 14 Mediation analysis of metabolomic signatures in the dietary-BAG association 39](#_Toc220523220)

[Supplementary Table 15 Subgroup analysis of association between AHEI-2010 diet and BAG (per SD Increase in dietary score) 40](#_Toc220523221)

[Supplementary Table 16 Subgroup analysis of association between DASH diet and BAG (per SD Increase in dietary score) 41](#_Toc220523222)

[Supplementary Table 17 Sensitivity analysis: further adjustment for additional covariates (diabetes, cardiovascular disease, and lipid-lowering medication use) 42](#_Toc220523223)

[Supplementary Table 18 Sensitivity analysis: association of metabolomic signatures and BAG in individuals with complete metabolomic data but missing dietary data 42](#_Toc220523224)

[Supplementary Table 19 The association between MED and BAG after removing the score of wine intake 43](#_Toc220523225)

[Supplementary Table 20 Correspondence table of metabolite full names and abbreviations 44](#_Toc220523226)

[**Scoring Criteria for Dietary Patterns** 51](#_Toc220523227)


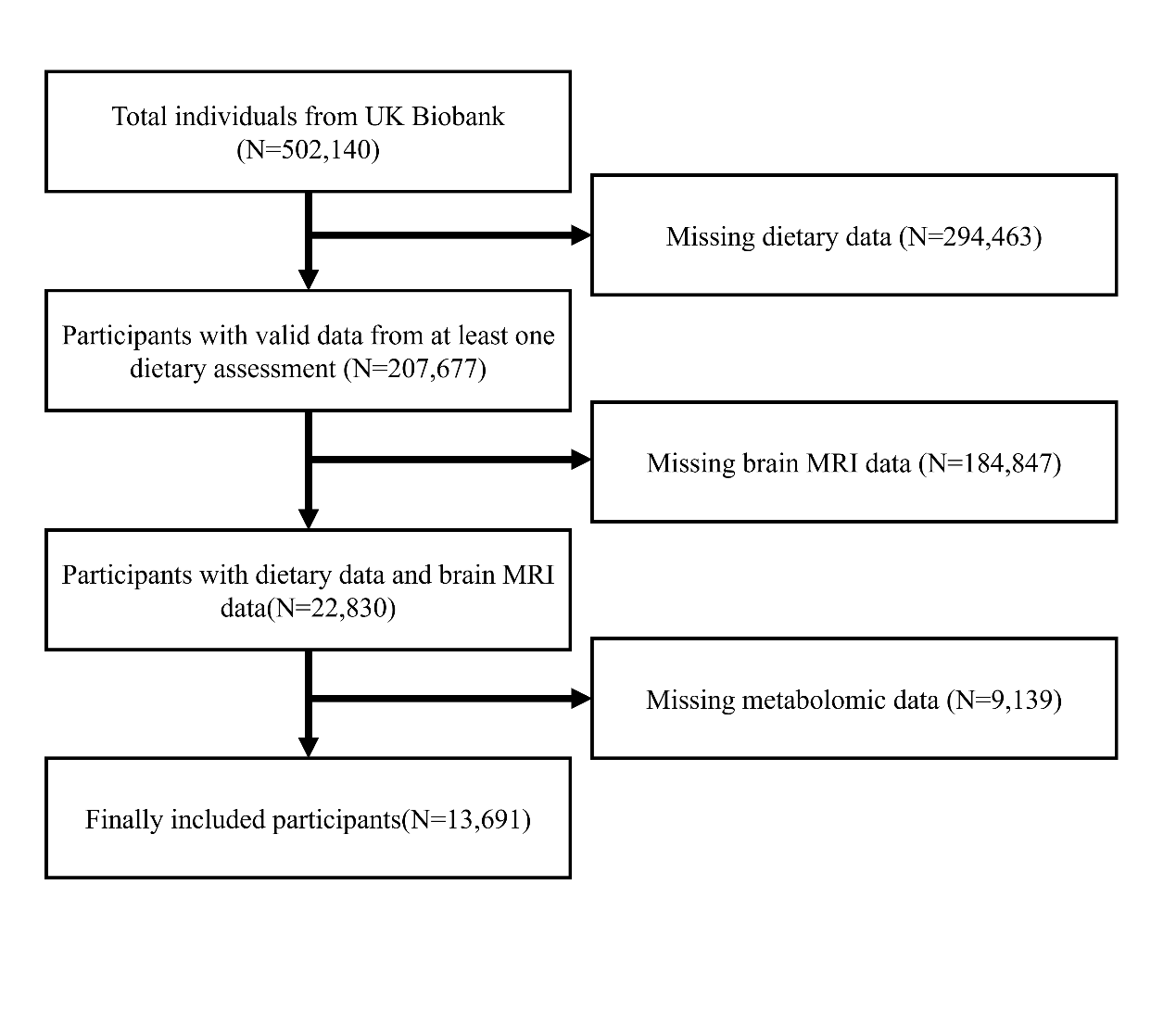


# Supplementary Figure 1 Participant flow diagram

# Supplementary Table 1 Key performance indicators of the brain age prediction model

| R2 | MAE | MSE | Pearson’s r | Correction Formula |
| --- | --- | --- | --- | --- |
| 0.715 | 3.240 | 17.007 | 0.845 | BA_correct_=(BA_predict_-β)/α |

MAE: Mean Absolute Error; MSE: Mean Squared Error; BA: Brain Age

# Supplementary Table 2 Missing rates of covariates undergoing imputation

| Characteristic | Missing rate |
| --- | --- |
| age | 0% |
| sex | 0% |
| race and ethnicity | 2.62% |
| Townsend deprivation index | 0.07% |
| smoking status | 0.03% |
| alcohol consumption | 0.04% |
| physical activity level | 7.33% |
| BMI category | 0.10% |
| education level | 0.23% |
| employment status | 6.00% |
| Standard PRS for AD | 0.82% |

# Supplementary Table 3 LASSO cross-validation metrics for dietary metabolomic signatures

| Dietary pattern | Lambda.min | Number of selected metabolites | |
| --- | --- | --- | --- |
|  |  | Before regularization | After regularization |
| AHEI-2010 | 0.00605 | 95 | 56 |
| DASH | 0.000405 | 56 | 46 |

# Supplementary Table 4 Mean and Standard Deviation of Dietary Scores

|  | AHEI-2010 | DASH | e-DII | MED | MIND |
| --- | --- | --- | --- | --- | --- |
| Mean ± SD | 58.33 ± 13.87 | 24.23 ± 4.90 | -0.59 ± 1.13 | 5.29 ± 1.93 | 6.52 ± 2.02 |

# Supplementary Table 5 Baseline characteristics of participants across tertiles of the DASH diet score

| Characteristics | Low (N=4966) | Medium (N=4217) | High (N=4508) | P |
| --- | --- | --- | --- | --- |
| Age, Mean(SD) | 53.4 ± 7.6 | 55.1 ± 7.4 | 56.2 ± 7.2 | <0.001 |
| Sex, N(%) |  |  |  | <0.001 |
| Female | 2309 (46.5%) | 2386 (56.6%) | 2653 (58.9%) |  |
| Male | 2657 (53.5%) | 1831 (43.4%) | 1855 (41.1%) |  |
| Race and ethnicity, N(%) |  |  |  | 0.120 |
| White | 4832 (97.3%) | 4120 (97.7%) | 4406 (97.7%) |  |
| Black | 58 (1.2%) | 46 (1.1%) | 46 (1%) |  |
| Asian | 35 (0.7%) | 12 (0.3%) | 18 (0.4%) |  |
| Other Race | 41 (0.8%) | 39 (0.9%) | 38 (0.8%) |  |
| Employment, N(%) |  |  |  | <0.001 |
| Unemployed | 235 (4.7%) | 204 (4.8%) | 232 (5.1%) |  |
| Employed | 2698 (54.3%) | 1972 (46.8%) | 1879 (41.7%) |  |
| Retired | 2033 (40.9%) | 2041 (48.4%) | 2397 (53.2%) |  |
| Educational attainment, N(%) |  |  |  | <0.001 |
| College or above | 2178 (43.9%) | 2182 (51.7%) | 2495 (55.3%) |  |
| A/AS levels or equivalent or O levels/GCSE or CSE or equivalent | 1772 (35.7%) | 1331 (31.6%) | 1311 (29.1%) |  |
| NVQ or HND or HNC or equivalent or other professional qualifications | 743 (15%) | 493 (11.7%) | 507 (11.2%) |  |
| Other | 273 (5.5%) | 211 (5%) | 195 (4.3%) |  |
| Alcohol use, N(%) |  |  |  | 0.853 |
| Yes | 4760 (95.9%) | 4032 (95.6%) | 4316 (95.7%) |  |
| No | 206 (4.1%) | 185 (4.4%) | 192 (4.3%) |  |
| Smoking, N(%) |  |  |  | <0.001 |
| Yes | 365 (7.3%) | 217 (5.1%) | 183 (4.1%) |  |
| No | 4601 (92.7%) | 4000 (94.9%) | 4325 (95.9%) |  |
| BMI, N(%) |  |  |  | <0.001 |
| <25 | 1699 (34.2%) | 1786 (42.4%) | 2225 (49.4%) |  |
| 25 to 30 | 2206 (44.4%) | 1790 (42.4%) | 1757 (39%) |  |
| ≥30 | 1061 (21.4%) | 641 (15.2%) | 526 (11.7%) |  |
| Waist, Mean(SD) | 89.8 ± 12.7 | 86.7 ± 12.2 | 85.0 ± 11.8 | <0.001 |
| LTPA, N(%) |  |  |  | <0.001 |
| None | 3344 (67.3%) | 2970 (70.4%) | 3376 (74.9%) |  |
| Inactive | 1556 (31.3%) | 1200 (28.5%) | 1083 (24%) |  |
| Active | 66 (1.3%) | 47 (1.1%) | 49 (1.1%) |  |
| Townsend deprivation index, Mean(SD) | -1.9 ± 2.7 | -2.1 ± 2.6 | -2.1 ± 2.6 | <0.001 |
| Standard PRS for AD, Mean(SD) | 0.0 ± 1.0 | 0.0 ± 1.0 | 0.1 ± 1.0 | 0.199 |
| BAG, Mean(SD) | 0.1 ± 4.9 | -0.3 ± 4.9 | -0.5 ± 4.8 | <0.001 |

# Supplementary Table 6 Baseline characteristics of participants across tertiles of the e-DII diet score

| Characteristics | Low (N=4564) | Medium (N=4563) | High (N=4564) | P |
| --- | --- | --- | --- | --- |
| Age, Mean(SD) | 55.7 ± 7.3 | 55.2 ± 7.6 | 53.8 ± 7.6 | <0.001 |
| Sex, N(%) |  |  |  | <0.001 |
| Female | 2960 (64.9%) | 2426 (53.2%) | 1962 (43%) |  |
| Male | 1604 (35.1%) | 2137 (46.8%) | 2602 (57%) |  |
| Race and ethnicity, N(%) |  |  |  | 0.232 |
| White | 4443 (97.3%) | 4473 (98%) | 4442 (97.3%) |  |
| Black | 53 (1.2%) | 46 (1%) | 51 (1.1%) |  |
| Asian | 24 (0.5%) | 17 (0.4%) | 24 (0.5%) |  |
| Other Race | 44 (1%) | 27 (0.6%) | 47 (1%) |  |
| Employment, N(%) |  |  |  | <0.001 |
| Unemployed | 244 (5.3%) | 204 (4.5%) | 223 (4.9%) |  |
| Employed | 1996 (43.7%) | 2157 (47.3%) | 2396 (52.5%) |  |
| Retired | 2324 (50.9%) | 2202 (48.3%) | 1945 (42.6%) |  |
| Educational attainment, N(%) |  |  |  |  |
| College or above | 2318 (50.8%) | 2361 (51.7%) | 2176 (47.7%) | 0.006 |
| A/AS levels or equivalent or O levels/GCSE or CSE or equivalent | 1462 (32%) | 1424 (31.2%) | 1528 (33.5%) |  |
| NVQ or HND or HNC or equivalent or other professional qualifications | 557 (12.2%) | 558 (12.2%) | 628 (13.8%) |  |
| Other | 227 (5%) | 220 (4.8%) | 232 (5.1%) |  |
| Alcohol use, N(%) |  |  |  | <0.001 |
| Yes | 4352 (95.4%) | 4420 (96.9%) | 4336 (95%) |  |
| No | 212 (4.6%) | 143 (3.1%) | 228 (5%) |  |
| Smoking, N(%) |  |  |  | <0.001 |
| Yes | 195 (4.3%) | 235 (5.2%) | 335 (7.3%) |  |
| No | 4369 (95.7%) | 4328 (94.8%) | 4229 (92.7%) |  |
| BMI, N(%) |  |  |  | <0.001 |
| <25 | 2074 (45.4%) | 1943 (42.6%) | 1693 (37.1%) |  |
| 25 to 30 | 1843 (40.4%) | 1893 (41.5%) | 2017 (44.2%) |  |
| ≥30 | 647 (14.2%) | 727 (15.9%) | 854 (18.7%) |  |
| Waist, Mean(SD) | 85.1 ± 12.2 | 87.2 ± 12.2 | 89.6 ± 12.5 | <0.001 |
| LTPA, N(%) |  |  |  | 0.609 |
| None | 3262 (71.5%) | 3227 (70.7%) | 3201 (70.1%) |  |
| Inactive | 1251 (27.4%) | 1277 (28%) | 1311 (28.7%) |  |
| Active | 51 (1.1%) | 59 (1.3%) | 52 (1.1%) |  |
| Townsend deprivation index, Mean(SD) | -2.2 ± 2.6 | -2.1 ± 2.6 | -1.9 ± 2.7 | <0.001 |
| Standard PRS for AD, Mean(SD) | 0.1 ± 1.0 | 0.0 ± 1.0 | 0.0 ± 1.0 | 0.003 |
| BAG, Mean(SD) | -0.3 ± 4.8 | -0.3 ± 4.9 | -0.1 ± 4.9 | 0.205 |

# Supplementary Table 7 Baseline characteristics of participants across tertiles of the MED diet score

| Characteristics | Low (N=4814) | Medium (N=5170) | High (N=3707) | P |
| --- | --- | --- | --- | --- |
| Age, Mean(SD) | 54.3 ± 7.6 | 54.8 ± 7.5 | 55.8 ± 7.4 | <0.001 |
| Sex, N(%) |  |  |  | <0.001 |
| Female | 2257 (46.9%) | 2869 (55.5%) | 2222 (59.9%) |  |
| Male | 2557 (53.1%) | 2301 (44.5%) | 1485 (40.1%) |  |
| Race and ethnicity, N(%) |  |  |  | 0.462 |
| White | 4710 (97.8%) | 5042 (97.5%) | 3606 (97.3%) |  |
| Black | 45 (0.9%) | 64 (1.2%) | 41 (1.1%) |  |
| Asian | 23 (0.5%) | 22 (0.4%) | 20 (0.5%) |  |
| Other Race | 36 (0.7%) | 42 (0.8%) | 40 (1.1%) |  |
| Employment, N(%) |  |  |  | <0.001 |
| Unemployed | 228 (4.7%) | 260 (5%) | 183 (4.9%) |  |
| Employed | 2439 (50.7%) | 2502 (48.4%) | 1608 (43.4%) |  |
| Retired | 2147 (44.6%) | 2408 (46.6%) | 1916 (51.7%) |  |
| Educational attainment, N(%) |  |  |  | <0.001 |
| College or above | 2074 (43.1%) | 2656 (51.4%) | 2125 (57.3%) |  |
| A/AS levels or equivalent or O levels/GCSE or CSE or equivalent | 1709 (35.5%) | 1638 (31.7%) | 1067 (28.8%) |  |
| NVQ or HND or HNC or equivalent or other professional qualifications | 733 (15.2%) | 621 (12%) | 389 (10.5%) |  |
| Other | 298 (6.2%) | 255 (4.9%) | 126 (3.4%) |  |
| Alcohol use, N(%) |  |  |  | <0.001 |
| Yes | 4554 (94.6%) | 4956 (95.9%) | 3598 (97.1%) |  |
| No | 260 (5.4%) | 214 (4.1%) | 109 (2.9%) |  |
| Smoking, N(%) |  |  |  | 0.004 |
| Yes | 287 (6%) | 311 (6%) | 167 (4.5%) |  |
| No | 4527 (94%) | 4859 (94%) | 3540 (95.5%) |  |
| BMI, N(%) |  |  |  | <0.001 |
| <25 | 1709 (35.5%) | 2233 (43.2%) | 1768 (47.7%) |  |
| 25 to 30 | 2126 (44.2%) | 2143 (41.5%) | 1484 (40%) |  |
| ≥30 | 979 (20.3%) | 794 (15.4%) | 455 (12.3%) |  |
| Waist, Mean(SD) | 89.5 ± 12.9 | 86.8 ± 12.2 | 85.1 ± 11.7 | <0.001 |
| LTPA, N(%) |  |  |  | <0.001 |
| None | 3299 (68.5%) | 3645 (70.5%) | 2746 (74.1%) |  |
| Inactive | 1455 (30.2%) | 1464 (28.3%) | 920 (24.8%) |  |
| Active | 60 (1.2%) | 61 (1.2%) | 41 (1.1%) |  |
| Townsend deprivation index, Mean(SD) | -1.9 ± 2.7 | -2.1 ± 2.6 | -2.1 ± 2.6 | 0.007 |
| Standard PRS for AD, Mean(SD) | 0.0 ± 1.0 | 0.0 ± 1.0 | 0.1 ± 1.0 | 0.065 |
| BAG, Mean(SD) | -0.2 ± 4.9 | -0.3 ± 4.9 | -0.2 ± 4.8 | 0.341 |

# Supplementary Table 8 Baseline characteristics of participants across tertiles of the MIND diet score

| Characteristics | Low (N=4876) | Medium (N=4999) | High (N=3816) | P |
| --- | --- | --- | --- | --- |
| Age, Mean(SD) | 53.8 ± 7.6 | 54.9 ± 7.5 | 56.2 ± 7.3 | <0.001 |
| Sex, N(%) |  |  |  | <0.001 |
| Female | 2254 (46.2%) | 2778 (55.6%) | 2316 (60.7%) |  |
| Male | 2622 (53.8%) | 2221 (44.4%) | 1500 (39.3%) |  |
| Race and ethnicity, N(%) |  |  |  | 0.097 |
| White | 4776 (97.9%) | 4869 (97.4%) | 3713 (97.3%) |  |
| Black | 52 (1.1%) | 58 (1.2%) | 40 (1%) |  |
| Asian | 22 (0.5%) | 23 (0.5%) | 20 (0.5%) |  |
| Other Race | 26 (0.5%) | 49 (1%) | 43 (1.1%) |  |
| Employment, N(%) |  |  |  | <0.001 |
| Unemployed | 226 (4.6%) | 260 (5.2%) | 185 (4.8%) |  |
| Employed | 2563 (52.6%) | 2385 (47.7%) | 1601 (42%) |  |
| Retired | 2087 (42.8%) | 2354 (47.1%) | 2030 (53.2%) |  |
| Educational attainment, N(%) |  |  |  | <0.001 |
| College or above | 2034 (41.7%) | 2629 (52.6%) | 2192 (57.4%) |  |
| A/AS levels or equivalent or O levels/GCSE or CSE or equivalent | 1765 (36.2%) | 1553 (31.1%) | 1096 (28.7%) |  |
| NVQ or HND or HNC or equivalent or other professional qualifications | 759 (15.6%) | 596 (11.9%) | 388 (10.2%) |  |
| Other | 318 (6.5%) | 221 (4.4%) | 140 (3.7%) |  |
| Alcohol use, N(%) |  |  |  | <0.001 |
| Yes | 4594 (94.2%) | 4810 (96.2%) | 3704 (97.1%) |  |
| No | 282 (5.8%) | 189 (3.8%) | 112 (2.9%) |  |
| Smoking, N(%) |  |  |  | <0.001 |
| Yes | 333 (6.8%) | 261 (5.2%) | 171 (4.5%) |  |
| No | 4543 (93.2%) | 4738 (94.8%) | 3645 (95.5%) |  |
| BMI, N(%) |  |  |  | <0.001 |
| <25 | 1733 (35.5%) | 2125 (42.5%) | 1852 (48.5%) |  |
| 25 to 30 | 2157 (44.2%) | 2091 (41.8%) | 1505 (39.4%) |  |
| ≥30 | 986 (20.2%) | 783 (15.7%) | 459 (12%) |  |
| Waist, Mean(SD) | 89.5 ± 12.7 | 86.9 ± 12.4 | 84.9 ± 11.6 | <0.001 |
| LTPA, N(%) |  |  |  | <0.001 |
| None | 3314 (68%) | 3577 (71.6%) | 2799 (73.3%) |  |
| Inactive | 1496 (30.7%) | 1373 (27.5%) | 970 (25.4%) |  |
| Active | 66 (1.4%) | 49 (1%) | 47 (1.2%) |  |
| Townsend deprivation index, Mean(SD) | -1.9 ± 2.7 | -2.1 ± 2.6 | -2.1 ± 2.6 | 0.002 |
| Standard PRS for AD, Mean(SD) | 0.0 ± 1.0 | 0.0 ± 1.0 | 0.1 ± 1.0 | 0.104 |
| BAG, Mean(SD) | -0.1 ± 4.8 | -0.3 ± 5.0 | -0.4 ± 4.8 | 0.085 |

# Supplementary Table 9 Effect of a SD Increase in Dietary Component Intake on BAG

| Variable | Model | β (95% CI) | P value |
| --- | --- | --- | --- |
| vegetables | Model 1 | -0.020 (-0.102, 0.061) | 0.629 |
| vegetables | Model 2 | 0.046 (-0.037, 0.129) | 0.278 |
| vegetables | Model 3 | 0.043 (-0.041, 0.126) | 0.316 |
| fruit (including fruit juice) | Model 1 | -0.151 (-0.232, -0.069) | <0.001 |
| fruit (including fruit juice) | Model 2 | -0.041 (-0.124, 0.042) | 0.336 |
| fruit (including fruit juice) | Model 3 | -0.044 (-0.128, 0.039) | 0.296 |
| red and processed meat | Model 1 | 0.112 (0.030, 0.193) | 0.007 |
| red and processed meat | Model 2 | 0.048 (-0.034, 0.130) | 0.251 |
| red and processed meat | Model 3 | 0.047 (-0.035, 0.129) | 0.262 |
| nuts and legumes | Model 1 | -0.041 (-0.123, 0.040) | 0.320 |
| nuts and legumes | Model 2 | -0.019 (-0.100, 0.063) | 0.652 |
| nuts and legumes | Model 3 | -0.022 (-0.103, 0.060) | 0.604 |
| sugary drinks and fruit juices | Model 1 | 0.083 (0.001, 0.165) | 0.046 |
| sugary drinks and fruit juices | Model 2 | 0.082 (0.000, 0.163) | 0.049 |
| sugary drinks and fruit juices | Model 3 | 0.080 (-0.001, 0.162) | 0.053 |
| whole grains | Model 1 | -0.208 (-0.289, -0.126) | <0.001 |
| whole grains | Model 2 | -0.136 (-0.218, -0.054) | 0.001 |
| whole grains | Model 3 | -0.137 (-0.219, -0.055) | 0.001 |
| trans fat | Model 1 | -0.097 (-0.179, -0.016) | 0.019 |
| trans fat | Model 2 | -0.106 (-0.188, -0.025) | 0.011 |
| trans fat | Model 3 | -0.115 (-0.197, -0.034) | 0.006 |
| long chain (n-3) fats | Model 1 | 0.028 (-0.054, 0.109) | 0.505 |
| long chain (n-3) fats | Model 2 | 0.061 (-0.020, 0.142) | 0.141 |
| long chain (n-3) fats | Model 3 | 0.059 (-0.022, 0.141) | 0.153 |
| PUFA | Model 1 | -0.136 (-0.218, -0.054) | 0.001 |
| PUFA | Model 2 | -0.125 (-0.206, -0.043) | 0.003 |
| PUFA | Model 3 | -0.133 (-0.215, -0.052) | 0.001 |
| sodium | Model 1 | 0.033 (-0.048, 0.115) | 0.422 |
| sodium | Model 2 | -0.029 (-0.110, 0.053) | 0.493 |
| sodium | Model 3 | -0.028 (-0.110, 0.053) | 0.496 |
| alcohol | Model 1 | 0.540 (0.458, 0.621) | <0.001 |
| alcohol | Model 2 | 0.499 (0.416, 0.582) | <0.001 |
| alcohol | Model 3 | 0.496 (0.413, 0.580) | <0.001 |
| low-fat dairy | Model 1 | -0.177 (-0.258, -0.095) | <0.001 |
| low-fat dairy | Model 2 | -0.141 (-0.223, -0.059) | 0.001 |
| low-fat dairy | Model 3 | -0.143 (-0.225, -0.061) | 0.001 |

# Supplementary Table 10 Detailed information of metabolomic signature of AHEI-2010 in our study

| Category | Shorten name | Weight | Estimate | Std. Error | t value |
| --- | --- | --- | --- | --- | --- |
| Fatty acids | LA_pct | 61.585 | 8.483 | 0.891 | 9.516 |
| Fatty acids | PUFA_pct | 38.930 | 13.659 | 1.104 | 12.376 |
| Proteins | Cholines | 37.116 | -3.976 | 0.703 | -5.659 |
| Lipoprotein | M_HDL_P | 13.520 | -4.742 | 0.550 | -8.628 |
| Lipoprotein | M_LDL_FC_pct | 10.116 | 7.344 | 1.293 | 5.679 |
| Fatty acids | PUFA_by_MUFA | 7.982 | 7.560 | 0.635 | 11.902 |
| Lipoprotein | IDL_FC_pct | 7.591 | 10.416 | 1.977 | 5.267 |
| Lipoprotein | IDL_TG | 7.493 | -1.918 | 0.493 | -3.894 |
| Phospholipids | M_HDL_PL | 6.309 | -6.330 | 0.629 | -10.059 |
| Lipoprotein | M_VLDL_TG_pct | 5.781 | -3.998 | 0.747 | -5.350 |
| Lipoprotein | S_HDL_TG | 5.619 | -2.733 | 0.370 | -7.379 |
| Lipoprotein | S_LDL_FC_pct | 3.706 | 4.691 | 1.036 | 4.528 |
| Lipoprotein | HDL_FC | 3.117 | -2.383 | 0.577 | -4.131 |
| Lipoprotein | L_VLDL_C_pct | 3.091 | 3.714 | 0.780 | 4.762 |
| Lipoprotein | XL_HDL_P | 2.220 | 1.572 | 0.354 | 4.448 |
| Fatty acids | DHA | 1.223 | 2.935 | 0.299 | 9.801 |
| Lipoprotein | L_VLDL_P | 0.920 | -0.825 | 0.212 | -3.898 |
| Lipoprotein | XL_VLDL_L | 0.742 | -0.656 | 0.154 | -4.252 |
| Lipoprotein | XXL_VLDL_L | 0.609 | -0.386 | 0.101 | -3.826 |
| Fatty acids | Omega_3_pct | 0.411 | 4.381 | 0.320 | 13.696 |
| Phospholipids | HDL_PL | 0.326 | -4.909 | 0.667 | -7.362 |
| Lipoprotein | XL_HDL_TG_pct | 0.310 | -1.376 | 0.254 | -5.424 |
| Lipoprotein | XL_HDL_FC | 0.246 | 2.669 | 0.438 | 6.096 |
| Lipoprotein | XXL_VLDL_CE | 0.231 | -0.621 | 0.119 | -5.231 |
| Fatty acids | DHA_pct | 0.000 | 3.909 | 0.305 | 12.830 |
| Lipoprotein | L_LDL_TG | -0.001 | -2.456 | 0.498 | -4.933 |
| Lipoprotein | M_VLDL_TG | -0.074 | -1.182 | 0.294 | -4.021 |
| Lipoprotein | XXL_VLDL_FC | -0.205 | -0.511 | 0.118 | -4.319 |
| Lipoprotein | L_HDL_TG_pct | -0.264 | -1.284 | 0.260 | -4.945 |
| Lipoprotein | XL_VLDL_TG | -0.373 | -0.541 | 0.123 | -4.394 |
| Lipoprotein | XXL_VLDL_P | -0.509 | -0.355 | 0.084 | -4.206 |
| Phospholipids | M_LDL_PL_pct | -0.819 | 18.417 | 3.720 | 4.950 |
| Lipoprotein | Total_TG | -0.872 | -1.571 | 0.302 | -5.201 |
| Lipoprotein | HDL_TG | -1.049 | -2.980 | 0.372 | -8.017 |
| Lipoprotein | M_VLDL_CE_pct | -1.316 | 1.464 | 0.307 | 4.764 |
| Lipoprotein | XL_HDL_CE | -1.436 | 1.019 | 0.254 | 4.017 |
| Lipoprotein | Total_L | -1.448 | -3.497 | 0.655 | -5.340 |
| Lipoprotein | LDL_TG | -1.648 | -2.238 | 0.473 | -4.728 |
| Lipoprotein | S_HDL_FC_pct | -2.470 | 17.164 | 2.438 | 7.042 |
| Fatty acids | Omega_6_by_Omega_3 | -2.791 | -3.425 | 0.310 | -11.037 |
| Lipoprotein | M_HDL_CE | -3.553 | -4.301 | 0.553 | -7.777 |
| Lipoprotein | M_LDL_TG | -3.803 | -1.908 | 0.433 | -4.409 |
| Lipoprotein | VLDL_size | -7.097 | -19.462 | 4.197 | -4.637 |
| Phospholipids | L_HDL_PL_pct | -7.778 | -21.558 | 2.439 | -8.838 |
| Lipoprotein | M_HDL_TG_pct | -8.718 | -1.572 | 0.347 | -4.526 |
| Phospholipids | Sphingomyelins | -9.060 | -3.046 | 0.711 | -4.287 |
| Fatty acids | SFA_pct | -13.137 | -18.378 | 1.412 | -13.019 |
| Phospholipids | IDL_PL_pct | -18.262 | 27.172 | 3.149 | 8.628 |
| Lipoprotein | HDL_P | -19.200 | -7.466 | 0.801 | -9.320 |
| Phospholipids | Phosphatidylc | -23.338 | -3.824 | 0.641 | -5.968 |
| Lipoprotein | IDL_CE_pct | -24.791 | -11.301 | 2.669 | -4.233 |
| Lipoprotein | Total_P | -26.091 | -7.601 | 0.845 | -8.997 |
| Phospholipids | S_LDL_PL_pct | -26.690 | 16.790 | 2.177 | 7.711 |
| Phospholipids | XS_VLDL_PL_pct | -28.054 | 14.964 | 3.975 | 3.764 |
| Lipoprotein | S_LDL_CE_pct | -46.269 | -22.278 | 2.693 | -8.272 |
| Fatty acids | Omega_6_pct | -104.321 | 7.628 | 1.030 | 7.405 |

# Supplementary Table 11 Detailed information of metabolomic signature of DASH in our study

| Category | Shorten name | Weight | Estimate | Std. Error | t value |
| --- | --- | --- | --- | --- | --- |
| Lipoprotein | HDL_size | 40.527 | 12.601 | 2.341 | 5.382 |
| Fatty acids | PUFA_pct | 22.185 | 3.140 | 0.386 | 8.141 |
| Fatty acids | LA_pct | 18.023 | 1.851 | 0.311 | 5.947 |
| Lipoprotein | S_LDL_C_pct | 12.769 | -6.984 | 1.369 | -5.102 |
| Lipoprotein | S_HDL_CE | 12.063 | -2.540 | 0.282 | -8.998 |
| Lipoprotein | S_HDL_FC_pct | 9.420 | 4.379 | 0.849 | 5.154 |
| Phospholipids | M_HDL_PL | 9.114 | -0.914 | 0.220 | -4.155 |
| Lipoprotein | M_HDL_P | 6.367 | -0.727 | 0.192 | -3.784 |
| Lipoprotein | Total_L | 4.020 | -0.875 | 0.228 | -3.836 |
| Lipoprotein | M_LDL_C | 3.228 | -0.644 | 0.147 | -4.388 |
| Fatty acids | SFA_pct | 3.142 | -2.789 | 0.494 | -5.641 |
| Phospholipids | IDL_PL_pct | 2.957 | 6.244 | 1.100 | 5.678 |
| Lipoprotein | S_HDL_TG | 2.873 | -0.572 | 0.129 | -4.441 |
| Proteins | Albumin | 2.342 | 1.769 | 0.465 | 3.803 |
| Lipoprotein | XL_HDL_L | 2.107 | 0.648 | 0.104 | 6.235 |
| Lipoprotein | M_LDL_L | 1.275 | -0.682 | 0.154 | -4.442 |
| Lipoprotein | S_HDL_C | 1.274 | -2.620 | 0.298 | -8.801 |
| Lipoprotein | XL_HDL_FC_pct | 0.838 | -0.922 | 0.233 | -3.954 |
| Lipoprotein | LDL_L | 0.441 | -0.648 | 0.173 | -3.756 |
| Lipoprotein | XL_HDL_FC | 0.271 | 0.965 | 0.152 | 6.341 |
| Lipoprotein | XXL_VLDL_CE | 0.044 | -0.162 | 0.041 | -3.930 |
| Lipoprotein | XL_HDL_C | 0.001 | 0.687 | 0.118 | 5.828 |
| Lipoprotein | XL_HDL_TG_pct | -0.018 | -0.388 | 0.088 | -4.402 |
| Fatty acids | MUFA_pct | -0.046 | -2.277 | 0.353 | -6.453 |
| Fatty acids | DHA_pct | -0.110 | 1.033 | 0.106 | 9.708 |
| Fatty acids | Omega_3 | -0.110 | 0.664 | 0.095 | 6.978 |
| Fatty acids | Omega_3_pct | -0.134 | 1.164 | 0.112 | 10.422 |
| Phospholipids | XL_HDL_PL | -0.147 | 0.271 | 0.059 | 4.600 |
| Lipoprotein | XL_HDL_CE | -0.187 | 0.403 | 0.088 | 4.562 |
| Proteins | GlycA | -0.352 | -1.253 | 0.306 | -4.098 |
| Lipoprotein | S_LDL_CE | -0.445 | -0.643 | 0.166 | -3.865 |
| Fatty acids | DHA | -0.448 | 0.778 | 0.104 | 7.453 |
| Lipoprotein | VLDL_size | -0.450 | -6.281 | 1.456 | -4.314 |
| Lipoprotein | XL_HDL_P | -1.267 | 0.652 | 0.123 | 5.301 |
| Phospholipids | L_HDL_PL_pct | -1.276 | -3.996 | 0.852 | -4.689 |
| Lipoprotein | M_HDL_TG | -1.453 | -0.438 | 0.116 | -3.760 |
| Fatty acids | MUFA | -1.926 | -0.867 | 0.152 | -5.706 |
| Fatty acids | Omega_6_by_Omega_3 | -2.013 | -0.914 | 0.108 | -8.439 |
| Phospholipids | S_LDL_PL_pct | -3.151 | 4.784 | 0.758 | 6.312 |
| Lipoprotein | M_HDL_CE | -3.877 | -0.742 | 0.193 | -3.840 |
| Fatty acids | Unsaturation | -4.184 | 3.809 | 0.504 | 7.557 |
| Phospholipids | S_HDL_PL | -5.230 | -2.634 | 0.303 | -8.695 |
| Lipoprotein | LDL_CE | -8.528 | -0.625 | 0.163 | -3.833 |
| Lipoprotein | S_LDL_CE_pct | -13.725 | -5.971 | 0.937 | -6.372 |
| Lipoprotein | HDL_P | -22.037 | -1.581 | 0.280 | -5.650 |
| Fatty acids | Omega_6_pct | -31.262 | 1.628 | 0.359 | 4.529 |

# Supplementary Table 12 Association between AHEI diet components and individual metabolites in metabolomic signature

| Metabolite | Diet |  | P |
| --- | --- | --- | --- |
| VLDL_size | vegetables | -0.055 (-0.093, -0.017) | 0.005 |
| L_VLDL_C_pct | vegetables | 0.010 (-0.023, 0.043) | 0.563 |
| XXL_VLDL_CE | vegetables | -0.024 (-0.059, 0.012) | 0.193 |
| M_HDL_CE | vegetables | 0.064 (0.027, 0.100) | <0.001 |
| XL_HDL_CE | vegetables | 0.074 (0.034, 0.114) | <0.001 |
| IDL_CE_pct | vegetables | -0.045 (-0.079, -0.012) | 0.007 |
| M_VLDL_CE_pct | vegetables | 0.004 (-0.032, 0.040) | 0.839 |
| S_LDL_CE_pct | vegetables | -0.037 (-0.070, -0.004) | 0.029 |
| XXL_VLDL_P | vegetables | -0.020 (-0.055, 0.016) | 0.280 |
| HDL_P | vegetables | 0.045 (0.010, 0.080) | 0.011 |
| L_VLDL_P | vegetables | -0.024 (-0.060, 0.012) | 0.185 |
| M_HDL_P | vegetables | 0.064 (0.028, 0.100) | <0.001 |
| XL_HDL_P | vegetables | 0.069 (0.030, 0.108) | <0.001 |
| DHA | vegetables | 0.191 (0.157, 0.225) | <0.001 |
| DHA_pct | vegetables | 0.193 (0.159, 0.227) | <0.001 |
| XXL_VLDL_FC | vegetables | -0.013 (-0.049, 0.022) | 0.455 |
| HDL_FC | vegetables | 0.079 (0.041, 0.118) | <0.001 |
| XL_HDL_FC | vegetables | 0.045 (0.008, 0.083) | 0.018 |
| IDL_FC_pct | vegetables | -0.008 (-0.041, 0.025) | 0.631 |
| M_LDL_FC_pct | vegetables | 0.015 (-0.021, 0.051) | 0.412 |
| S_HDL_FC_pct | vegetables | 0.047 (0.012, 0.082) | 0.009 |
| S_LDL_FC_pct | vegetables | -0.022 (-0.056, 0.012) | 0.199 |
| LA_pct | vegetables | -0.015 (-0.050, 0.020) | 0.396 |
| Omega_3_pct | vegetables | 0.207 (0.174, 0.240) | <0.001 |
| Omega_6_by_Omega_3 | vegetables | -0.146 (-0.178, -0.113) | <0.001 |
| Omega_6_pct | vegetables | 0.001 (-0.034, 0.036) | 0.969 |
| Phosphatidylc | vegetables | 0.047 (0.013, 0.082) | 0.007 |
| HDL_PL | vegetables | 0.077 (0.040, 0.114) | <0.001 |
| M_HDL_PL | vegetables | 0.063 (0.028, 0.098) | <0.001 |
| IDL_PL_pct | vegetables | 0.036 (0.004, 0.069) | 0.027 |
| L_HDL_PL_pct | vegetables | -0.001 (-0.037, 0.035) | 0.954 |
| M_LDL_PL_pct | vegetables | 0.036 (0.004, 0.069) | 0.029 |
| S_LDL_PL_pct | vegetables | 0.040 (0.007, 0.074) | 0.018 |
| XS_VLDL_PL_pct | vegetables | 0.025 (-0.009, 0.058) | 0.148 |
| PUFA_by_MUFA | vegetables | 0.091 (0.054, 0.127) | <0.001 |
| PUFA_pct | vegetables | 0.098 (0.063, 0.134) | <0.001 |
| SFA_pct | vegetables | -0.100 (-0.132, -0.067) | <0.001 |
| Sphingomyelins | vegetables | 0.015 (-0.019, 0.049) | 0.396 |
| Cholines | vegetables | 0.044 (0.009, 0.078) | 0.012 |
| Total_P | vegetables | 0.039 (0.005, 0.073) | 0.026 |
| XXL_VLDL_L | vegetables | -0.025 (-0.060, 0.011) | 0.168 |
| Total_L | vegetables | 0.004 (-0.028, 0.037) | 0.791 |
| XL_VLDL_L | vegetables | -0.032 (-0.068, 0.004) | 0.083 |
| Total_TG | vegetables | -0.011 (-0.046, 0.024) | 0.544 |
| HDL_TG | vegetables | 0.014 (-0.019, 0.048) | 0.399 |
| IDL_TG | vegetables | 0.029 (-0.004, 0.063) | 0.088 |
| LDL_TG | vegetables | 0.019 (-0.014, 0.053) | 0.260 |
| L_LDL_TG | vegetables | 0.023 (-0.011, 0.057) | 0.178 |
| M_LDL_TG | vegetables | 0.017 (-0.018, 0.051) | 0.342 |
| M_VLDL_TG | vegetables | -0.015 (-0.050, 0.021) | 0.419 |
| S_HDL_TG | vegetables | -0.009 (-0.045, 0.026) | 0.604 |
| XL_VLDL_TG | vegetables | -0.031 (-0.067, 0.006) | 0.097 |
| L_HDL_TG_pct | vegetables | -0.016 (-0.052, 0.020) | 0.380 |
| M_HDL_TG_pct | vegetables | -0.019 (-0.055, 0.017) | 0.296 |
| M_VLDL_TG_pct | vegetables | 0.003 (-0.032, 0.038) | 0.871 |
| XL_HDL_TG_pct | vegetables | -0.011 (-0.047, 0.024) | 0.536 |
| VLDL_size | fruit (not fruit juice) | -0.027 (-0.061, 0.007) | 0.119 |
| L_VLDL_C_pct | fruit (not fruit juice) | 0.034 (0.004, 0.063) | 0.026 |
| XXL_VLDL_CE | fruit (not fruit juice) | -0.023 (-0.055, 0.009) | 0.151 |
| M_HDL_CE | fruit (not fruit juice) | -0.114 (-0.146, -0.081) | <0.001 |
| XL_HDL_CE | fruit (not fruit juice) | 0.024 (-0.011, 0.060) | 0.181 |
| IDL_CE_pct | fruit (not fruit juice) | -0.095 (-0.125, -0.065) | <0.001 |
| M_VLDL_CE_pct | fruit (not fruit juice) | -0.003 (-0.035, 0.030) | 0.864 |
| S_LDL_CE_pct | fruit (not fruit juice) | -0.059 (-0.089, -0.029) | <0.001 |
| XXL_VLDL_P | fruit (not fruit juice) | -0.006 (-0.038, 0.026) | 0.710 |
| HDL_P | fruit (not fruit juice) | -0.123 (-0.154, -0.092) | <0.001 |
| L_VLDL_P | fruit (not fruit juice) | -0.007 (-0.039, 0.025) | 0.672 |
| M_HDL_P | fruit (not fruit juice) | -0.103 (-0.135, -0.070) | <0.001 |
| XL_HDL_P | fruit (not fruit juice) | 0.034 (-0.001, 0.069) | 0.054 |
| DHA | fruit (not fruit juice) | 0.129 (0.099, 0.159) | <0.001 |
| DHA_pct | fruit (not fruit juice) | 0.182 (0.151, 0.212) | <0.001 |
| XXL_VLDL_FC | fruit (not fruit juice) | -0.007 (-0.039, 0.024) | 0.648 |
| HDL_FC | fruit (not fruit juice) | -0.060 (-0.094, -0.025) | <0.001 |
| XL_HDL_FC | fruit (not fruit juice) | 0.058 (0.024, 0.091) | <0.001 |
| IDL_FC_pct | fruit (not fruit juice) | 0.016 (-0.014, 0.045) | 0.293 |
| M_LDL_FC_pct | fruit (not fruit juice) | -0.017 (-0.049, 0.015) | 0.301 |
| S_HDL_FC_pct | fruit (not fruit juice) | 0.060 (0.028, 0.091) | <0.001 |
| S_LDL_FC_pct | fruit (not fruit juice) | -0.023 (-0.054, 0.007) | 0.135 |
| LA_pct | fruit (not fruit juice) | 0.003 (-0.028, 0.035) | 0.831 |
| Omega_3_pct | fruit (not fruit juice) | 0.192 (0.163, 0.222) | <0.001 |
| Omega_6_by_Omega_3 | fruit (not fruit juice) | -0.149 (-0.179, -0.120) | <0.001 |
| Omega_6_pct | fruit (not fruit juice) | 0.008 (-0.023, 0.040) | 0.609 |
| Phosphatidylc | fruit (not fruit juice) | -0.063 (-0.094, -0.032) | <0.001 |
| HDL_PL | fruit (not fruit juice) | -0.080 (-0.113, -0.046) | <0.001 |
| M_HDL_PL | fruit (not fruit juice) | -0.096 (-0.128, -0.065) | <0.001 |
| IDL_PL_pct | fruit (not fruit juice) | 0.096 (0.067, 0.125) | <0.001 |
| L_HDL_PL_pct | fruit (not fruit juice) | -0.026 (-0.058, 0.006) | 0.117 |
| M_LDL_PL_pct | fruit (not fruit juice) | -0.018 (-0.047, 0.011) | 0.229 |
| S_LDL_PL_pct | fruit (not fruit juice) | 0.061 (0.031, 0.091) | <0.001 |
| XS_VLDL_PL_pct | fruit (not fruit juice) | 0.062 (0.032, 0.092) | <0.001 |
| PUFA_by_MUFA | fruit (not fruit juice) | 0.112 (0.079, 0.145) | <0.001 |
| PUFA_pct | fruit (not fruit juice) | 0.099 (0.067, 0.131) | <0.001 |
| SFA_pct | fruit (not fruit juice) | -0.062 (-0.092, -0.033) | <0.001 |
| Sphingomyelins | fruit (not fruit juice) | -0.075 (-0.105, -0.044) | <0.001 |
| Cholines | fruit (not fruit juice) | -0.069 (-0.100, -0.038) | <0.001 |
| Total_P | fruit (not fruit juice) | -0.120 (-0.151, -0.089) | <0.001 |
| XXL_VLDL_L | fruit (not fruit juice) | -0.010 (-0.042, 0.022) | 0.528 |
| Total_L | fruit (not fruit juice) | -0.053 (-0.082, -0.023) | <0.001 |
| XL_VLDL_L | fruit (not fruit juice) | -0.010 (-0.042, 0.023) | 0.565 |
| Total_TG | fruit (not fruit juice) | -0.006 (-0.038, 0.026) | 0.724 |
| HDL_TG | fruit (not fruit juice) | 0.005 (-0.025, 0.035) | 0.761 |
| IDL_TG | fruit (not fruit juice) | 0.018 (-0.012, 0.048) | 0.237 |
| LDL_TG | fruit (not fruit juice) | -0.007 (-0.037, 0.024) | 0.665 |
| L_LDL_TG | fruit (not fruit juice) | -0.005 (-0.036, 0.025) | 0.731 |
| M_LDL_TG | fruit (not fruit juice) | -0.012 (-0.043, 0.019) | 0.451 |
| M_VLDL_TG | fruit (not fruit juice) | -0.015 (-0.047, 0.017) | 0.355 |
| S_HDL_TG | fruit (not fruit juice) | -0.008 (-0.041, 0.024) | 0.614 |
| XL_VLDL_TG | fruit (not fruit juice) | -0.011 (-0.044, 0.021) | 0.501 |
| L_HDL_TG_pct | fruit (not fruit juice) | 0.040 (0.008, 0.073) | 0.014 |
| M_HDL_TG_pct | fruit (not fruit juice) | 0.057 (0.025, 0.089) | <0.001 |
| M_VLDL_TG_pct | fruit (not fruit juice) | -0.001 (-0.033, 0.031) | 0.939 |
| XL_HDL_TG_pct | fruit (not fruit juice) | -0.002 (-0.034, 0.030) | 0.904 |
| VLDL_size | red and processed meat | 0.020 (0.003, 0.037) | 0.021 |
| L_VLDL_C_pct | red and processed meat | -0.004 (-0.018, 0.011) | 0.634 |
| XXL_VLDL_CE | red and processed meat | 0.016 (0.000, 0.032) | 0.046 |
| M_HDL_CE | red and processed meat | 0.025 (0.009, 0.041) | 0.003 |
| XL_HDL_CE | red and processed meat | -0.020 (-0.038, -0.002) | 0.026 |
| IDL_CE_pct | red and processed meat | 0.019 (0.004, 0.034) | 0.011 |
| M_VLDL_CE_pct | red and processed meat | -0.014 (-0.030, 0.002) | 0.093 |
| S_LDL_CE_pct | red and processed meat | 0.029 (0.014, 0.044) | <0.001 |
| XXL_VLDL_P | red and processed meat | 0.017 (0.001, 0.033) | 0.039 |
| HDL_P | red and processed meat | 0.031 (0.015, 0.046) | <0.001 |
| L_VLDL_P | red and processed meat | 0.018 (0.002, 0.035) | 0.025 |
| M_HDL_P | red and processed meat | 0.030 (0.014, 0.046) | <0.001 |
| XL_HDL_P | red and processed meat | -0.017 (-0.035, 0.000) | 0.050 |
| DHA | red and processed meat | 0.005 (-0.010, 0.021) | 0.476 |
| DHA_pct | red and processed meat | -0.012 (-0.027, 0.004) | 0.138 |
| XXL_VLDL_FC | red and processed meat | 0.017 (0.001, 0.032) | 0.039 |
| HDL_FC | red and processed meat | 0.015 (-0.003, 0.032) | 0.098 |
| XL_HDL_FC | red and processed meat | -0.021 (-0.038, -0.004) | 0.013 |
| IDL_FC_pct | red and processed meat | -0.009 (-0.023, 0.006) | 0.243 |
| M_LDL_FC_pct | red and processed meat | -0.014 (-0.030, 0.002) | 0.083 |
| S_HDL_FC_pct | red and processed meat | -0.024 (-0.039, -0.008) | 0.003 |
| S_LDL_FC_pct | red and processed meat | -0.011 (-0.026, 0.005) | 0.171 |
| LA_pct | red and processed meat | -0.064 (-0.079, -0.048) | <0.001 |
| Omega_3_pct | red and processed meat | 0.009 (-0.006, 0.023) | 0.259 |
| Omega_6_by_Omega_3 | red and processed meat | -0.031 (-0.045, -0.016) | <0.001 |
| Omega_6_pct | red and processed meat | -0.039 (-0.055, -0.023) | <0.001 |
| Phosphatidylc | red and processed meat | 0.024 (0.008, 0.039) | 0.002 |
| HDL_PL | red and processed meat | 0.027 (0.010, 0.043) | 0.002 |
| M_HDL_PL | red and processed meat | 0.035 (0.019, 0.051) | <0.001 |
| IDL_PL_pct | red and processed meat | -0.028 (-0.042, -0.013) | <0.001 |
| L_HDL_PL_pct | red and processed meat | 0.042 (0.026, 0.058) | <0.001 |
| M_LDL_PL_pct | red and processed meat | -0.014 (-0.028, 0.001) | 0.064 |
| S_LDL_PL_pct | red and processed meat | -0.027 (-0.042, -0.013) | <0.001 |
| XS_VLDL_PL_pct | red and processed meat | -0.021 (-0.036, -0.006) | 0.007 |
| PUFA_by_MUFA | red and processed meat | -0.026 (-0.042, -0.010) | 0.002 |
| PUFA_pct | red and processed meat | -0.034 (-0.050, -0.018) | <0.001 |
| SFA_pct | red and processed meat | 0.027 (0.012, 0.041) | <0.001 |
| Sphingomyelins | red and processed meat | 0.020 (0.005, 0.036) | 0.009 |
| Cholines | red and processed meat | 0.023 (0.008, 0.039) | 0.003 |
| Total_P | red and processed meat | 0.031 (0.015, 0.046) | <0.001 |
| XXL_VLDL_L | red and processed meat | 0.017 (0.001, 0.033) | 0.038 |
| Total_L | red and processed meat | 0.022 (0.008, 0.037) | 0.003 |
| XL_VLDL_L | red and processed meat | 0.019 (0.003, 0.036) | 0.018 |
| Total_TG | red and processed meat | 0.019 (0.003, 0.035) | 0.017 |
| HDL_TG | red and processed meat | 0.025 (0.010, 0.040) | 0.001 |
| IDL_TG | red and processed meat | 0.010 (-0.005, 0.025) | 0.184 |
| LDL_TG | red and processed meat | 0.011 (-0.005, 0.026) | 0.170 |
| L_LDL_TG | red and processed meat | 0.011 (-0.004, 0.026) | 0.156 |
| M_LDL_TG | red and processed meat | 0.010 (-0.006, 0.025) | 0.221 |
| M_VLDL_TG | red and processed meat | 0.019 (0.003, 0.035) | 0.017 |
| S_HDL_TG | red and processed meat | 0.025 (0.009, 0.041) | 0.002 |
| XL_VLDL_TG | red and processed meat | 0.020 (0.004, 0.036) | 0.014 |
| L_HDL_TG_pct | red and processed meat | 0.011 (-0.005, 0.027) | 0.187 |
| M_HDL_TG_pct | red and processed meat | 0.011 (-0.005, 0.027) | 0.181 |
| M_VLDL_TG_pct | red and processed meat | 0.011 (-0.005, 0.027) | 0.179 |
| XL_HDL_TG_pct | red and processed meat | 0.016 (0.000, 0.032) | 0.050 |
| VLDL_size | nuts and legumes | -0.011 (-0.023, 0.001) | 0.069 |
| L_VLDL_C_pct | nuts and legumes | 0.003 (-0.007, 0.013) | 0.538 |
| XXL_VLDL_CE | nuts and legumes | -0.009 (-0.020, 0.002) | 0.108 |
| M_HDL_CE | nuts and legumes | -0.001 (-0.013, 0.010) | 0.840 |
| XL_HDL_CE | nuts and legumes | 0.010 (-0.002, 0.023) | 0.097 |
| IDL_CE_pct | nuts and legumes | 0.003 (-0.007, 0.013) | 0.586 |
| M_VLDL_CE_pct | nuts and legumes | 0.019 (0.008, 0.030) | <0.001 |
| S_LDL_CE_pct | nuts and legumes | -0.010 (-0.021, 0.000) | 0.050 |
| XXL_VLDL_P | nuts and legumes | -0.011 (-0.022, 0.000) | 0.061 |
| HDL_P | nuts and legumes | -0.004 (-0.015, 0.007) | 0.494 |
| L_VLDL_P | nuts and legumes | -0.010 (-0.021, 0.001) | 0.088 |
| M_HDL_P | nuts and legumes | -0.006 (-0.017, 0.006) | 0.324 |
| XL_HDL_P | nuts and legumes | 0.005 (-0.007, 0.017) | 0.403 |
| DHA | nuts and legumes | 0.008 (-0.002, 0.019) | 0.122 |
| DHA_pct | nuts and legumes | 0.014 (0.004, 0.025) | 0.008 |
| XXL_VLDL_FC | nuts and legumes | -0.009 (-0.020, 0.002) | 0.114 |
| HDL_FC | nuts and legumes | -0.002 (-0.014, 0.010) | 0.740 |
| XL_HDL_FC | nuts and legumes | 0.004 (-0.008, 0.016) | 0.513 |
| IDL_FC_pct | nuts and legumes | 0.005 (-0.005, 0.015) | 0.341 |
| M_LDL_FC_pct | nuts and legumes | 0.019 (0.008, 0.031) | <0.001 |
| S_HDL_FC_pct | nuts and legumes | 0.004 (-0.007, 0.015) | 0.437 |
| S_LDL_FC_pct | nuts and legumes | 0.014 (0.004, 0.025) | 0.008 |
| LA_pct | nuts and legumes | 0.050 (0.039, 0.061) | <0.001 |
| Omega_3_pct | nuts and legumes | 0.005 (-0.006, 0.015) | 0.369 |
| Omega_6_by_Omega_3 | nuts and legumes | 0.011 (0.001, 0.021) | 0.037 |
| Omega_6_pct | nuts and legumes | 0.039 (0.028, 0.050) | <0.001 |
| Phosphatidylc | nuts and legumes | -0.006 (-0.017, 0.004) | 0.244 |
| HDL_PL | nuts and legumes | -0.006 (-0.018, 0.005) | 0.293 |
| M_HDL_PL | nuts and legumes | -0.010 (-0.021, 0.001) | 0.085 |
| IDL_PL_pct | nuts and legumes | 0.011 (0.000, 0.021) | 0.040 |
| L_HDL_PL_pct | nuts and legumes | -0.016 (-0.028, -0.005) | 0.004 |
| M_LDL_PL_pct | nuts and legumes | 0.017 (0.006, 0.027) | 0.001 |
| S_LDL_PL_pct | nuts and legumes | 0.011 (0.001, 0.022) | 0.035 |
| XS_VLDL_PL_pct | nuts and legumes | 0.000 (-0.010, 0.011) | 0.954 |
| PUFA_by_MUFA | nuts and legumes | 0.027 (0.015, 0.038) | <0.001 |
| PUFA_pct | nuts and legumes | 0.040 (0.029, 0.051) | <0.001 |
| SFA_pct | nuts and legumes | -0.045 (-0.055, -0.035) | <0.001 |
| Sphingomyelins | nuts and legumes | -0.007 (-0.017, 0.004) | 0.216 |
| Cholines | nuts and legumes | -0.004 (-0.015, 0.007) | 0.445 |
| Total_P | nuts and legumes | -0.004 (-0.015, 0.007) | 0.465 |
| XXL_VLDL_L | nuts and legumes | -0.010 (-0.021, 0.001) | 0.071 |
| Total_L | nuts and legumes | -0.008 (-0.018, 0.003) | 0.143 |
| XL_VLDL_L | nuts and legumes | -0.012 (-0.023, 0.000) | 0.043 |
| Total_TG | nuts and legumes | -0.013 (-0.024, -0.001) | 0.026 |
| HDL_TG | nuts and legumes | -0.021 (-0.032, -0.011) | <0.001 |
| IDL_TG | nuts and legumes | -0.014 (-0.025, -0.004) | 0.007 |
| LDL_TG | nuts and legumes | -0.013 (-0.023, -0.002) | 0.019 |
| L_LDL_TG | nuts and legumes | -0.014 (-0.024, -0.003) | 0.011 |
| M_LDL_TG | nuts and legumes | -0.011 (-0.022, 0.000) | 0.047 |
| M_VLDL_TG | nuts and legumes | -0.008 (-0.019, 0.003) | 0.150 |
| S_HDL_TG | nuts and legumes | -0.020 (-0.031, -0.008) | <0.001 |
| XL_VLDL_TG | nuts and legumes | -0.013 (-0.024, -0.001) | 0.029 |
| L_HDL_TG_pct | nuts and legumes | -0.010 (-0.021, 0.001) | 0.084 |
| M_HDL_TG_pct | nuts and legumes | -0.021 (-0.032, -0.009) | <0.001 |
| M_VLDL_TG_pct | nuts and legumes | -0.015 (-0.026, -0.004) | 0.009 |
| XL_HDL_TG_pct | nuts and legumes | -0.002 (-0.013, 0.010) | 0.791 |
| VLDL_size | sugary drinks and fruit juices | 0.037 (0.021, 0.053) | <0.001 |
| L_VLDL_C_pct | sugary drinks and fruit juices | -0.012 (-0.026, 0.002) | 0.087 |
| XXL_VLDL_CE | sugary drinks and fruit juices | 0.031 (0.016, 0.046) | <0.001 |
| M_HDL_CE | sugary drinks and fruit juices | -0.019 (-0.035, -0.004) | 0.014 |
| XL_HDL_CE | sugary drinks and fruit juices | -0.040 (-0.056, -0.023) | <0.001 |
| IDL_CE_pct | sugary drinks and fruit juices | -0.016 (-0.030, -0.002) | 0.024 |
| M_VLDL_CE_pct | sugary drinks and fruit juices | -0.035 (-0.050, -0.020) | <0.001 |
| S_LDL_CE_pct | sugary drinks and fruit juices | 0.015 (0.001, 0.029) | 0.041 |
| XXL_VLDL_P | sugary drinks and fruit juices | 0.028 (0.013, 0.043) | <0.001 |
| HDL_P | sugary drinks and fruit juices | -0.008 (-0.022, 0.007) | 0.306 |
| L_VLDL_P | sugary drinks and fruit juices | 0.036 (0.021, 0.051) | <0.001 |
| M_HDL_P | sugary drinks and fruit juices | -0.013 (-0.028, 0.002) | 0.087 |
| XL_HDL_P | sugary drinks and fruit juices | -0.032 (-0.049, -0.016) | <0.001 |
| DHA | sugary drinks and fruit juices | -0.005 (-0.019, 0.009) | 0.464 |
| DHA_pct | sugary drinks and fruit juices | -0.019 (-0.033, -0.005) | 0.010 |
| XXL_VLDL_FC | sugary drinks and fruit juices | 0.026 (0.011, 0.041) | <0.001 |
| HDL_FC | sugary drinks and fruit juices | -0.024 (-0.040, -0.007) | 0.004 |
| XL_HDL_FC | sugary drinks and fruit juices | -0.033 (-0.049, -0.017) | <0.001 |
| IDL_FC_pct | sugary drinks and fruit juices | -0.021 (-0.035, -0.007) | 0.003 |
| M_LDL_FC_pct | sugary drinks and fruit juices | -0.034 (-0.049, -0.019) | <0.001 |
| S_HDL_FC_pct | sugary drinks and fruit juices | -0.012 (-0.027, 0.003) | 0.106 |
| S_LDL_FC_pct | sugary drinks and fruit juices | -0.022 (-0.036, -0.008) | 0.002 |
| LA_pct | sugary drinks and fruit juices | -0.028 (-0.043, -0.014) | <0.001 |
| Omega_3_pct | sugary drinks and fruit juices | -0.008 (-0.022, 0.006) | 0.281 |
| Omega_6_by_Omega_3 | sugary drinks and fruit juices | 0.013 (-0.001, 0.027) | 0.071 |
| Omega_6_pct | sugary drinks and fruit juices | -0.037 (-0.052, -0.022) | <0.001 |
| Phosphatidylc | sugary drinks and fruit juices | 0.003 (-0.012, 0.017) | 0.729 |
| HDL_PL | sugary drinks and fruit juices | -0.016 (-0.031, 0.000) | 0.045 |
| M_HDL_PL | sugary drinks and fruit juices | -0.006 (-0.021, 0.009) | 0.420 |
| IDL_PL_pct | sugary drinks and fruit juices | 0.000 (-0.013, 0.014) | 0.993 |
| L_HDL_PL_pct | sugary drinks and fruit juices | 0.034 (0.019, 0.049) | <0.001 |
| M_LDL_PL_pct | sugary drinks and fruit juices | -0.010 (-0.024, 0.004) | 0.147 |
| S_LDL_PL_pct | sugary drinks and fruit juices | -0.017 (-0.031, -0.003) | 0.015 |
| XS_VLDL_PL_pct | sugary drinks and fruit juices | 0.015 (0.001, 0.029) | 0.039 |
| PUFA_by_MUFA | sugary drinks and fruit juices | -0.043 (-0.059, -0.028) | <0.001 |
| PUFA_pct | sugary drinks and fruit juices | -0.040 (-0.054, -0.025) | <0.001 |
| SFA_pct | sugary drinks and fruit juices | 0.019 (0.005, 0.032) | 0.007 |
| Sphingomyelins | sugary drinks and fruit juices | -0.005 (-0.019, 0.010) | 0.515 |
| Cholines | sugary drinks and fruit juices | 0.001 (-0.013, 0.015) | 0.891 |
| Total_P | sugary drinks and fruit juices | -0.005 (-0.020, 0.009) | 0.478 |
| XXL_VLDL_L | sugary drinks and fruit juices | 0.027 (0.012, 0.042) | <0.001 |
| Total_L | sugary drinks and fruit juices | 0.012 (-0.001, 0.026) | 0.079 |
| XL_VLDL_L | sugary drinks and fruit juices | 0.036 (0.021, 0.051) | <0.001 |
| Total_TG | sugary drinks and fruit juices | 0.036 (0.021, 0.051) | <0.001 |
| HDL_TG | sugary drinks and fruit juices | 0.030 (0.016, 0.044) | <0.001 |
| IDL_TG | sugary drinks and fruit juices | 0.028 (0.014, 0.042) | <0.001 |
| LDL_TG | sugary drinks and fruit juices | 0.029 (0.015, 0.044) | <0.001 |
| L_LDL_TG | sugary drinks and fruit juices | 0.027 (0.013, 0.042) | <0.001 |
| M_LDL_TG | sugary drinks and fruit juices | 0.031 (0.017, 0.046) | <0.001 |
| M_VLDL_TG | sugary drinks and fruit juices | 0.036 (0.022, 0.051) | <0.001 |
| S_HDL_TG | sugary drinks and fruit juices | 0.042 (0.027, 0.057) | <0.001 |
| XL_VLDL_TG | sugary drinks and fruit juices | 0.036 (0.021, 0.052) | <0.001 |
| L_HDL_TG_pct | sugary drinks and fruit juices | 0.040 (0.024, 0.055) | <0.001 |
| M_HDL_TG_pct | sugary drinks and fruit juices | 0.040 (0.025, 0.055) | <0.001 |
| M_VLDL_TG_pct | sugary drinks and fruit juices | 0.030 (0.015, 0.045) | <0.001 |
| XL_HDL_TG_pct | sugary drinks and fruit juices | 0.032 (0.017, 0.047) | <0.001 |
| VLDL_size | whole grains | -0.914 (-2.591, 0.764) | 0.286 |
| L_VLDL_C_pct | whole grains | 0.711 (-0.741, 2.162) | 0.337 |
| XXL_VLDL_CE | whole grains | -1.259 (-2.830, 0.312) | 0.116 |
| M_HDL_CE | whole grains | -5.293 (-6.903, -3.683) | <0.001 |
| XL_HDL_CE | whole grains | 1.401 (-0.354, 3.156) | 0.118 |
| IDL_CE_pct | whole grains | -3.308 (-4.776, -1.839) | <0.001 |
| M_VLDL_CE_pct | whole grains | 0.119 (-1.472, 1.711) | 0.883 |
| S_LDL_CE_pct | whole grains | -3.643 (-5.117, -2.169) | <0.001 |
| XXL_VLDL_P | whole grains | -0.808 (-2.377, 0.762) | 0.313 |
| HDL_P | whole grains | -5.927 (-7.460, -4.395) | <0.001 |
| L_VLDL_P | whole grains | -0.671 (-2.261, 0.919) | 0.408 |
| M_HDL_P | whole grains | -5.216 (-6.812, -3.621) | <0.001 |
| XL_HDL_P | whole grains | 1.448 (-0.268, 3.163) | 0.098 |
| DHA | whole grains | 4.912 (3.421, 6.402) | <0.001 |
| DHA_pct | whole grains | 7.135 (5.636, 8.635) | <0.001 |
| XXL_VLDL_FC | whole grains | -0.671 (-2.236, 0.893) | 0.400 |
| HDL_FC | whole grains | -3.238 (-4.934, -1.541) | <0.001 |
| XL_HDL_FC | whole grains | 2.286 (0.632, 3.940) | 0.007 |
| IDL_FC_pct | whole grains | -0.130 (-1.580, 1.319) | 0.860 |
| M_LDL_FC_pct | whole grains | 0.405 (-1.175, 1.984) | 0.616 |
| S_HDL_FC_pct | whole grains | 2.468 (0.918, 4.018) | 0.002 |
| S_LDL_FC_pct | whole grains | 0.121 (-1.378, 1.621) | 0.874 |
| LA_pct | whole grains | 4.345 (2.810, 5.880) | <0.001 |
| Omega_3_pct | whole grains | 7.334 (5.870, 8.798) | <0.001 |
| Omega_6_by_Omega_3 | whole grains | -4.945 (-6.398, -3.493) | <0.001 |
| Omega_6_pct | whole grains | 3.451 (1.897, 5.005) | <0.001 |
| Phosphatidylc | whole grains | -3.634 (-5.151, -2.116) | <0.001 |
| HDL_PL | whole grains | -4.271 (-5.910, -2.633) | <0.001 |
| M_HDL_PL | whole grains | -5.196 (-6.746, -3.645) | <0.001 |
| IDL_PL_pct | whole grains | 4.997 (3.577, 6.417) | <0.001 |
| L_HDL_PL_pct | whole grains | -2.449 (-4.043, -0.856) | 0.003 |
| M_LDL_PL_pct | whole grains | 0.989 (-0.448, 2.426) | 0.177 |
| S_LDL_PL_pct | whole grains | 3.511 (2.034, 4.988) | <0.001 |
| XS_VLDL_PL_pct | whole grains | 3.540 (2.061, 5.020) | <0.001 |
| PUFA_by_MUFA | whole grains | 6.271 (4.649, 7.892) | <0.001 |
| PUFA_pct | whole grains | 6.851 (5.287, 8.415) | <0.001 |
| SFA_pct | whole grains | -6.412 (-7.853, -4.971) | <0.001 |
| Sphingomyelins | whole grains | -4.057 (-5.573, -2.541) | <0.001 |
| Cholines | whole grains | -3.891 (-5.402, -2.380) | <0.001 |
| Total_P | whole grains | -5.848 (-7.367, -4.329) | <0.001 |
| XXL_VLDL_L | whole grains | -0.985 (-2.551, 0.582) | 0.218 |
| Total_L | whole grains | -3.091 (-4.544, -1.638) | <0.001 |
| XL_VLDL_L | whole grains | -0.855 (-2.455, 0.744) | 0.295 |
| Total_TG | whole grains | -0.771 (-2.334, 0.792) | 0.334 |
| HDL_TG | whole grains | -1.250 (-2.724, 0.224) | 0.097 |
| IDL_TG | whole grains | -0.612 (-2.094, 0.869) | 0.418 |
| LDL_TG | whole grains | -1.486 (-2.986, 0.013) | 0.052 |
| L_LDL_TG | whole grains | -1.654 (-3.141, -0.166) | 0.029 |
| M_LDL_TG | whole grains | -1.406 (-2.919, 0.106) | 0.068 |
| M_VLDL_TG | whole grains | -0.603 (-2.164, 0.958) | 0.449 |
| S_HDL_TG | whole grains | -1.474 (-3.059, 0.110) | 0.068 |
| XL_VLDL_TG | whole grains | -0.903 (-2.503, 0.698) | 0.269 |
| L_HDL_TG_pct | whole grains | 0.032 (-1.553, 1.618) | 0.968 |
| M_HDL_TG_pct | whole grains | 0.948 (-0.629, 2.525) | 0.239 |
| M_VLDL_TG_pct | whole grains | -0.029 (-1.588, 1.530) | 0.971 |
| XL_HDL_TG_pct | whole grains | -0.853 (-2.423, 0.716) | 0.287 |
| VLDL_size | trans fat | 0.003 (-0.001, 0.008) | 0.141 |
| L_VLDL_C_pct | trans fat | 0.000 (-0.004, 0.003) | 0.810 |
| XXL_VLDL_CE | trans fat | 0.003 (-0.001, 0.007) | 0.121 |
| M_HDL_CE | trans fat | 0.001 (-0.004, 0.005) | 0.758 |
| XL_HDL_CE | trans fat | 0.005 (0.001, 0.010) | 0.028 |
| IDL_CE_pct | trans fat | 0.006 (0.002, 0.010) | 0.004 |
| M_VLDL_CE_pct | trans fat | 0.001 (-0.003, 0.005) | 0.613 |
| S_LDL_CE_pct | trans fat | 0.005 (0.001, 0.009) | 0.017 |
| XXL_VLDL_P | trans fat | 0.004 (-0.001, 0.008) | 0.090 |
| HDL_P | trans fat | 0.002 (-0.002, 0.006) | 0.263 |
| L_VLDL_P | trans fat | 0.004 (0.000, 0.008) | 0.065 |
| M_HDL_P | trans fat | 0.002 (-0.002, 0.007) | 0.271 |
| XL_HDL_P | trans fat | 0.007 (0.003, 0.012) | 0.002 |
| DHA | trans fat | -0.016 (-0.020, -0.012) | <0.001 |
| DHA_pct | trans fat | -0.020 (-0.024, -0.017) | <0.001 |
| XXL_VLDL_FC | trans fat | 0.003 (-0.001, 0.007) | 0.171 |
| HDL_FC | trans fat | 0.005 (0.000, 0.009) | 0.038 |
| XL_HDL_FC | trans fat | 0.007 (0.002, 0.011) | 0.002 |
| IDL_FC_pct | trans fat | 0.002 (-0.002, 0.006) | 0.313 |
| M_LDL_FC_pct | trans fat | -0.001 (-0.005, 0.003) | 0.680 |
| S_HDL_FC_pct | trans fat | 0.005 (0.001, 0.009) | 0.020 |
| S_LDL_FC_pct | trans fat | -0.002 (-0.006, 0.002) | 0.342 |
| LA_pct | trans fat | 0.002 (-0.003, 0.006) | 0.466 |
| Omega_3_pct | trans fat | -0.019 (-0.023, -0.016) | <0.001 |
| Omega_6_by_Omega_3 | trans fat | 0.014 (0.010, 0.017) | <0.001 |
| Omega_6_pct | trans fat | -0.004 (-0.008, 0.000) | 0.055 |
| Phosphatidylc | trans fat | 0.004 (0.000, 0.008) | 0.038 |
| HDL_PL | trans fat | 0.003 (-0.001, 0.008) | 0.130 |
| M_HDL_PL | trans fat | 0.002 (-0.002, 0.006) | 0.418 |
| IDL_PL_pct | trans fat | -0.005 (-0.009, -0.002) | 0.005 |
| L_HDL_PL_pct | trans fat | -0.004 (-0.008, 0.000) | 0.059 |
| M_LDL_PL_pct | trans fat | -0.004 (-0.007, 0.000) | 0.064 |
| S_LDL_PL_pct | trans fat | -0.004 (-0.008, 0.000) | 0.036 |
| XS_VLDL_PL_pct | trans fat | -0.006 (-0.010, -0.003) | 0.001 |
| PUFA_by_MUFA | trans fat | -0.009 (-0.013, -0.004) | <0.001 |
| PUFA_pct | trans fat | -0.013 (-0.017, -0.009) | <0.001 |
| SFA_pct | trans fat | 0.014 (0.010, 0.018) | <0.001 |
| Sphingomyelins | trans fat | 0.004 (0.000, 0.008) | 0.044 |
| Cholines | trans fat | 0.004 (0.000, 0.008) | 0.071 |
| Total_P | trans fat | 0.003 (-0.001, 0.007) | 0.140 |
| XXL_VLDL_L | trans fat | 0.004 (0.000, 0.008) | 0.058 |
| Total_L | trans fat | 0.006 (0.002, 0.010) | 0.001 |
| XL_VLDL_L | trans fat | 0.004 (0.000, 0.008) | 0.051 |
| Total_TG | trans fat | 0.004 (0.000, 0.008) | 0.056 |
| HDL_TG | trans fat | 0.006 (0.002, 0.010) | 0.004 |
| IDL_TG | trans fat | 0.002 (-0.002, 0.006) | 0.245 |
| LDL_TG | trans fat | 0.003 (-0.001, 0.006) | 0.207 |
| L_LDL_TG | trans fat | 0.002 (-0.002, 0.006) | 0.238 |
| M_LDL_TG | trans fat | 0.002 (-0.002, 0.006) | 0.237 |
| M_VLDL_TG | trans fat | 0.004 (-0.001, 0.008) | 0.091 |
| S_HDL_TG | trans fat | 0.003 (-0.001, 0.007) | 0.140 |
| XL_VLDL_TG | trans fat | 0.004 (0.000, 0.008) | 0.063 |
| L_HDL_TG_pct | trans fat | 0.003 (-0.001, 0.007) | 0.121 |
| M_HDL_TG_pct | trans fat | 0.005 (0.001, 0.009) | 0.018 |
| M_VLDL_TG_pct | trans fat | -0.002 (-0.006, 0.002) | 0.268 |
| XL_HDL_TG_pct | trans fat | 0.001 (-0.004, 0.005) | 0.785 |
| VLDL_size | long chain (n-3) fats | -0.142 (-0.178, -0.106) | <0.001 |
| L_VLDL_C_pct | long chain (n-3) fats | 0.053 (0.022, 0.084) | <0.001 |
| XXL_VLDL_CE | long chain (n-3) fats | -0.093 (-0.127, -0.060) | <0.001 |
| M_HDL_CE | long chain (n-3) fats | 0.035 (0.000, 0.069) | 0.051 |
| XL_HDL_CE | long chain (n-3) fats | 0.114 (0.076, 0.152) | <0.001 |
| IDL_CE_pct | long chain (n-3) fats | -0.011 (-0.042, 0.021) | 0.506 |
| M_VLDL_CE_pct | long chain (n-3) fats | 0.100 (0.066, 0.135) | <0.001 |
| S_LDL_CE_pct | long chain (n-3) fats | -0.101 (-0.133, -0.070) | <0.001 |
| XXL_VLDL_P | long chain (n-3) fats | -0.096 (-0.130, -0.062) | <0.001 |
| HDL_P | long chain (n-3) fats | 0.016 (-0.017, 0.049) | 0.332 |
| L_VLDL_P | long chain (n-3) fats | -0.083 (-0.118, -0.049) | <0.001 |
| M_HDL_P | long chain (n-3) fats | 0.023 (-0.011, 0.058) | 0.187 |
| XL_HDL_P | long chain (n-3) fats | 0.098 (0.061, 0.134) | <0.001 |
| DHA | long chain (n-3) fats | 0.347 (0.315, 0.378) | <0.001 |
| DHA_pct | long chain (n-3) fats | 0.402 (0.371, 0.434) | <0.001 |
| XXL_VLDL_FC | long chain (n-3) fats | -0.096 (-0.130, -0.063) | <0.001 |
| HDL_FC | long chain (n-3) fats | 0.073 (0.037, 0.110) | <0.001 |
| XL_HDL_FC | long chain (n-3) fats | 0.095 (0.060, 0.131) | <0.001 |
| IDL_FC_pct | long chain (n-3) fats | 0.114 (0.083, 0.145) | <0.001 |
| M_LDL_FC_pct | long chain (n-3) fats | 0.115 (0.081, 0.149) | <0.001 |
| S_HDL_FC_pct | long chain (n-3) fats | 0.132 (0.098, 0.165) | <0.001 |
| S_LDL_FC_pct | long chain (n-3) fats | 0.092 (0.060, 0.125) | <0.001 |
| LA_pct | long chain (n-3) fats | -0.078 (-0.111, -0.045) | <0.001 |
| Omega_3_pct | long chain (n-3) fats | 0.383 (0.352, 0.414) | <0.001 |
| Omega_6_by_Omega_3 | long chain (n-3) fats | -0.268 (-0.299, -0.237) | <0.001 |
| Omega_6_pct | long chain (n-3) fats | -0.021 (-0.055, 0.012) | 0.209 |
| Phosphatidylc | long chain (n-3) fats | 0.009 (-0.024, 0.042) | 0.593 |
| HDL_PL | long chain (n-3) fats | 0.035 (0.000, 0.071) | 0.050 |
| M_HDL_PL | long chain (n-3) fats | 0.005 (-0.028, 0.039) | 0.755 |
| IDL_PL_pct | long chain (n-3) fats | 0.000 (-0.030, 0.031) | 0.994 |
| L_HDL_PL_pct | long chain (n-3) fats | -0.067 (-0.101, -0.033) | <0.001 |
| M_LDL_PL_pct | long chain (n-3) fats | 0.034 (0.003, 0.065) | 0.031 |
| S_LDL_PL_pct | long chain (n-3) fats | 0.084 (0.052, 0.116) | <0.001 |
| XS_VLDL_PL_pct | long chain (n-3) fats | -0.069 (-0.100, -0.037) | <0.001 |
| PUFA_by_MUFA | long chain (n-3) fats | 0.212 (0.177, 0.246) | <0.001 |
| PUFA_pct | long chain (n-3) fats | 0.160 (0.126, 0.194) | <0.001 |
| SFA_pct | long chain (n-3) fats | -0.061 (-0.092, -0.030) | <0.001 |
| Sphingomyelins | long chain (n-3) fats | 0.056 (0.024, 0.089) | <0.001 |
| Cholines | long chain (n-3) fats | 0.009 (-0.024, 0.041) | 0.606 |
| Total_P | long chain (n-3) fats | 0.016 (-0.017, 0.048) | 0.351 |
| XXL_VLDL_L | long chain (n-3) fats | -0.098 (-0.131, -0.064) | <0.001 |
| Total_L | long chain (n-3) fats | -0.008 (-0.039, 0.023) | 0.608 |
| XL_VLDL_L | long chain (n-3) fats | -0.095 (-0.129, -0.060) | <0.001 |
| Total_TG | long chain (n-3) fats | -0.077 (-0.111, -0.044) | <0.001 |
| HDL_TG | long chain (n-3) fats | -0.057 (-0.089, -0.025) | <0.001 |
| IDL_TG | long chain (n-3) fats | -0.015 (-0.047, 0.017) | 0.360 |
| LDL_TG | long chain (n-3) fats | -0.023 (-0.055, 0.009) | 0.161 |
| L_LDL_TG | long chain (n-3) fats | -0.013 (-0.045, 0.019) | 0.426 |
| M_LDL_TG | long chain (n-3) fats | -0.030 (-0.063, 0.002) | 0.070 |
| M_VLDL_TG | long chain (n-3) fats | -0.072 (-0.106, -0.038) | <0.001 |
| S_HDL_TG | long chain (n-3) fats | -0.083 (-0.117, -0.049) | <0.001 |
| XL_VLDL_TG | long chain (n-3) fats | -0.096 (-0.131, -0.062) | <0.001 |
| L_HDL_TG_pct | long chain (n-3) fats | -0.055 (-0.089, -0.021) | 0.002 |
| M_HDL_TG_pct | long chain (n-3) fats | -0.082 (-0.116, -0.048) | <0.001 |
| M_VLDL_TG_pct | long chain (n-3) fats | -0.091 (-0.124, -0.057) | <0.001 |
| XL_HDL_TG_pct | long chain (n-3) fats | -0.055 (-0.089, -0.021) | 0.001 |
| VLDL_size | PUFA | 0.047 (0.013, 0.081) | 0.007 |
| L_VLDL_C_pct | PUFA | 0.000 (-0.030, 0.029) | 0.979 |
| XXL_VLDL_CE | PUFA | -0.002 (-0.034, 0.030) | 0.883 |
| M_HDL_CE | PUFA | -0.066 (-0.099, -0.033) | <0.001 |
| XL_HDL_CE | PUFA | 0.014 (-0.022, 0.049) | 0.456 |
| IDL_CE_pct | PUFA | 0.003 (-0.027, 0.033) | 0.850 |
| M_VLDL_CE_pct | PUFA | 0.027 (-0.005, 0.060) | 0.100 |
| S_LDL_CE_pct | PUFA | -0.041 (-0.071, -0.011) | 0.007 |
| XXL_VLDL_P | PUFA | -0.012 (-0.044, 0.020) | 0.478 |
| HDL_P | PUFA | -0.058 (-0.089, -0.027) | <0.001 |
| L_VLDL_P | PUFA | 0.008 (-0.025, 0.040) | 0.643 |
| M_HDL_P | PUFA | -0.086 (-0.119, -0.054) | <0.001 |
| XL_HDL_P | PUFA | -0.005 (-0.040, 0.029) | 0.759 |
| DHA | PUFA | 0.039 (0.008, 0.069) | 0.012 |
| DHA_pct | PUFA | 0.040 (0.010, 0.071) | 0.010 |
| XXL_VLDL_FC | PUFA | -0.003 (-0.035, 0.029) | 0.860 |
| HDL_FC | PUFA | -0.066 (-0.100, -0.031) | <0.001 |
| XL_HDL_FC | PUFA | -0.012 (-0.046, 0.022) | 0.490 |
| IDL_FC_pct | PUFA | 0.010 (-0.019, 0.040) | 0.502 |
| M_LDL_FC_pct | PUFA | 0.060 (0.028, 0.092) | <0.001 |
| S_HDL_FC_pct | PUFA | 0.027 (-0.004, 0.059) | 0.092 |
| S_LDL_FC_pct | PUFA | 0.044 (0.014, 0.075) | 0.004 |
| LA_pct | PUFA | 0.274 (0.243, 0.305) | <0.001 |
| Omega_3_pct | PUFA | 0.036 (0.006, 0.066) | 0.018 |
| Omega_6_by_Omega_3 | PUFA | 0.016 (-0.014, 0.045) | 0.300 |
| Omega_6_pct | PUFA | 0.192 (0.161, 0.224) | <0.001 |
| Phosphatidylc | PUFA | -0.049 (-0.080, -0.018) | 0.002 |
| HDL_PL | PUFA | -0.094 (-0.127, -0.060) | <0.001 |
| M_HDL_PL | PUFA | -0.107 (-0.138, -0.075) | <0.001 |
| IDL_PL_pct | PUFA | 0.063 (0.034, 0.092) | <0.001 |
| L_HDL_PL_pct | PUFA | -0.093 (-0.126, -0.061) | <0.001 |
| M_LDL_PL_pct | PUFA | 0.101 (0.071, 0.130) | <0.001 |
| S_LDL_PL_pct | PUFA | 0.037 (0.007, 0.067) | 0.015 |
| XS_VLDL_PL_pct | PUFA | 0.042 (0.012, 0.073) | 0.006 |
| PUFA_by_MUFA | PUFA | 0.087 (0.054, 0.120) | <0.001 |
| PUFA_pct | PUFA | 0.205 (0.173, 0.237) | <0.001 |
| SFA_pct | PUFA | -0.309 (-0.338, -0.280) | <0.001 |
| Sphingomyelins | PUFA | -0.046 (-0.077, -0.015) | 0.003 |
| Cholines | PUFA | -0.036 (-0.067, -0.006) | 0.021 |
| Total_P | PUFA | -0.052 (-0.083, -0.021) | <0.001 |
| XXL_VLDL_L | PUFA | -0.013 (-0.045, 0.019) | 0.438 |
| Total_L | PUFA | -0.025 (-0.055, 0.005) | 0.097 |
| XL_VLDL_L | PUFA | -0.005 (-0.038, 0.027) | 0.745 |
| Total_TG | PUFA | -0.016 (-0.048, 0.016) | 0.331 |
| HDL_TG | PUFA | -0.099 (-0.129, -0.069) | <0.001 |
| IDL_TG | PUFA | -0.072 (-0.102, -0.041) | <0.001 |
| LDL_TG | PUFA | -0.046 (-0.076, -0.015) | 0.003 |
| L_LDL_TG | PUFA | -0.062 (-0.092, -0.032) | <0.001 |
| M_LDL_TG | PUFA | -0.025 (-0.055, 0.006) | 0.118 |
| M_VLDL_TG | PUFA | 0.022 (-0.010, 0.054) | 0.175 |
| S_HDL_TG | PUFA | -0.066 (-0.098, -0.033) | <0.001 |
| XL_VLDL_TG | PUFA | -0.012 (-0.044, 0.021) | 0.488 |
| L_HDL_TG_pct | PUFA | -0.043 (-0.075, -0.010) | 0.010 |
| M_HDL_TG_pct | PUFA | -0.074 (-0.106, -0.042) | <0.001 |
| M_VLDL_TG_pct | PUFA | -0.011 (-0.043, 0.021) | 0.495 |
| XL_HDL_TG_pct | PUFA | -0.003 (-0.035, 0.029) | 0.842 |
| VLDL_size | sodium | 11.736 (1.458, 22.013) | 0.025 |
| L_VLDL_C_pct | sodium | -5.601 (-14.493, 3.292) | 0.217 |
| XXL_VLDL_CE | sodium | 10.937 (1.312, 20.562) | 0.026 |
| M_HDL_CE | sodium | -3.597 (-13.477, 6.284) | 0.476 |
| XL_HDL_CE | sodium | -2.896 (-13.649, 7.857) | 0.598 |
| IDL_CE_pct | sodium | -0.475 (-9.480, 8.530) | 0.918 |
| M_VLDL_CE_pct | sodium | -3.848 (-13.600, 5.904) | 0.439 |
| S_LDL_CE_pct | sodium | 7.056 (-1.984, 16.095) | 0.126 |
| XXL_VLDL_P | sodium | 7.578 (-2.039, 17.194) | 0.122 |
| HDL_P | sodium | -0.270 (-9.679, 9.139) | 0.955 |
| L_VLDL_P | sodium | 9.754 (0.013, 19.495) | 0.050 |
| M_HDL_P | sodium | -4.006 (-13.797, 5.784) | 0.422 |
| XL_HDL_P | sodium | -3.166 (-13.677, 7.346) | 0.555 |
| DHA | sodium | -4.263 (-13.409, 4.882) | 0.361 |
| DHA_pct | sodium | -10.981 (-20.195, -1.767) | 0.020 |
| XXL_VLDL_FC | sodium | 9.012 (-0.572, 18.597) | 0.065 |
| HDL_FC | sodium | -5.024 (-15.423, 5.374) | 0.344 |
| XL_HDL_FC | sodium | -5.170 (-15.307, 4.966) | 0.317 |
| IDL_FC_pct | sodium | 2.509 (-6.372, 11.391) | 0.580 |
| M_LDL_FC_pct | sodium | -3.153 (-12.832, 6.525) | 0.523 |
| S_HDL_FC_pct | sodium | -2.623 (-12.123, 6.877) | 0.588 |
| S_LDL_FC_pct | sodium | -4.098 (-13.284, 5.087) | 0.382 |
| LA_pct | sodium | 12.139 (2.725, 21.554) | 0.012 |
| Omega_3_pct | sodium | -3.792 (-12.793, 5.210) | 0.409 |
| Omega_6_by_Omega_3 | sodium | 2.768 (-6.144, 11.680) | 0.543 |
| Omega_6_pct | sodium | 7.408 (-2.118, 16.935) | 0.127 |
| Phosphatidylc | sodium | -0.347 (-9.653, 8.958) | 0.942 |
| HDL_PL | sodium | -5.097 (-15.148, 4.955) | 0.320 |
| M_HDL_PL | sodium | -4.964 (-14.478, 4.549) | 0.306 |
| IDL_PL_pct | sodium | 1.463 (-7.250, 10.177) | 0.742 |
| L_HDL_PL_pct | sodium | -1.463 (-11.230, 8.303) | 0.769 |
| M_LDL_PL_pct | sodium | 4.122 (-4.682, 12.925) | 0.359 |
| S_LDL_PL_pct | sodium | -5.664 (-14.720, 3.392) | 0.220 |
| XS_VLDL_PL_pct | sodium | 7.778 (-1.293, 16.850) | 0.093 |
| PUFA_by_MUFA | sodium | -6.864 (-16.819, 3.092) | 0.177 |
| PUFA_pct | sodium | 5.455 (-4.154, 15.065) | 0.266 |
| SFA_pct | sodium | -21.599 (-30.443, -12.754) | <0.001 |
| Sphingomyelins | sodium | 1.085 (-8.214, 10.385) | 0.819 |
| Cholines | sodium | 2.026 (-7.239, 11.291) | 0.668 |
| Total_P | sodium | 0.766 (-8.561, 10.093) | 0.872 |
| XXL_VLDL_L | sodium | 7.539 (-2.059, 17.138) | 0.124 |
| Total_L | sodium | 4.623 (-4.285, 13.530) | 0.309 |
| XL_VLDL_L | sodium | 8.595 (-1.206, 18.396) | 0.086 |
| Total_TG | sodium | 7.222 (-2.355, 16.799) | 0.139 |
| HDL_TG | sodium | -1.128 (-10.159, 7.903) | 0.807 |
| IDL_TG | sodium | -0.989 (-10.066, 8.088) | 0.831 |
| LDL_TG | sodium | 2.440 (-6.746, 11.627) | 0.603 |
| L_LDL_TG | sodium | 0.446 (-8.671, 9.562) | 0.924 |
| M_LDL_TG | sodium | 4.809 (-4.462, 14.080) | 0.309 |
| M_VLDL_TG | sodium | 10.822 (1.261, 20.383) | 0.027 |
| S_HDL_TG | sodium | 3.431 (-6.279, 13.141) | 0.489 |
| XL_VLDL_TG | sodium | 7.349 (-2.459, 17.157) | 0.142 |
| L_HDL_TG_pct | sodium | 8.162 (-1.554, 17.877) | 0.100 |
| M_HDL_TG_pct | sodium | 0.755 (-8.909, 10.419) | 0.878 |
| M_VLDL_TG_pct | sodium | 3.860 (-5.690, 13.410) | 0.428 |
| XL_HDL_TG_pct | sodium | 7.508 (-2.109, 17.125) | 0.126 |
| VLDL_size | alcohol | -0.177 (-0.208, -0.146) | <0.001 |
| L_VLDL_C_pct | alcohol | -0.033 (-0.060, -0.006) | 0.017 |
| XXL_VLDL_CE | alcohol | -0.105 (-0.134, -0.076) | <0.001 |
| M_HDL_CE | alcohol | 0.461 (0.432, 0.490) | <0.001 |
| XL_HDL_CE | alcohol | 0.143 (0.110, 0.175) | <0.001 |
| IDL_CE_pct | alcohol | 0.134 (0.106, 0.161) | <0.001 |
| M_VLDL_CE_pct | alcohol | 0.044 (0.015, 0.074) | 0.003 |
| S_LDL_CE_pct | alcohol | 0.028 (0.001, 0.056) | 0.043 |
| XXL_VLDL_P | alcohol | -0.093 (-0.122, -0.064) | <0.001 |
| HDL_P | alcohol | 0.410 (0.382, 0.437) | <0.001 |
| L_VLDL_P | alcohol | -0.132 (-0.162, -0.103) | <0.001 |
| M_HDL_P | alcohol | 0.448 (0.420, 0.477) | <0.001 |
| XL_HDL_P | alcohol | 0.114 (0.082, 0.146) | <0.001 |
| DHA | alcohol | 0.184 (0.156, 0.211) | <0.001 |
| DHA_pct | alcohol | 0.141 (0.113, 0.169) | <0.001 |
| XXL_VLDL_FC | alcohol | -0.095 (-0.124, -0.066) | <0.001 |
| HDL_FC | alcohol | 0.388 (0.357, 0.419) | <0.001 |
| XL_HDL_FC | alcohol | 0.027 (-0.004, 0.058) | 0.083 |
| IDL_FC_pct | alcohol | -0.046 (-0.073, -0.019) | <0.001 |
| M_LDL_FC_pct | alcohol | 0.063 (0.033, 0.092) | <0.001 |
| S_HDL_FC_pct | alcohol | 0.006 (-0.022, 0.035) | 0.662 |
| S_LDL_FC_pct | alcohol | 0.024 (-0.004, 0.052) | 0.093 |
| LA_pct | alcohol | -0.220 (-0.249, -0.192) | <0.001 |
| Omega_3_pct | alcohol | 0.106 (0.079, 0.133) | <0.001 |
| Omega_6_by_Omega_3 | alcohol | -0.118 (-0.145, -0.091) | <0.001 |
| Omega_6_pct | alcohol | -0.128 (-0.157, -0.099) | <0.001 |
| Phosphatidylc | alcohol | 0.281 (0.253, 0.308) | <0.001 |
| HDL_PL | alcohol | 0.435 (0.406, 0.465) | <0.001 |
| M_HDL_PL | alcohol | 0.444 (0.416, 0.471) | <0.001 |
| IDL_PL_pct | alcohol | -0.190 (-0.217, -0.164) | <0.001 |
| L_HDL_PL_pct | alcohol | 0.094 (0.064, 0.123) | <0.001 |
| M_LDL_PL_pct | alcohol | -0.020 (-0.046, 0.007) | 0.149 |
| S_LDL_PL_pct | alcohol | -0.032 (-0.060, -0.005) | 0.022 |
| XS_VLDL_PL_pct | alcohol | -0.205 (-0.232, -0.178) | <0.001 |
| PUFA_by_MUFA | alcohol | 0.038 (0.008, 0.068) | 0.014 |
| PUFA_pct | alcohol | -0.076 (-0.105, -0.046) | <0.001 |
| SFA_pct | alcohol | 0.230 (0.203, 0.257) | <0.001 |
| Sphingomyelins | alcohol | 0.218 (0.190, 0.246) | <0.001 |
| Cholines | alcohol | 0.264 (0.236, 0.291) | <0.001 |
| Total_P | alcohol | 0.379 (0.351, 0.406) | <0.001 |
| XXL_VLDL_L | alcohol | -0.094 (-0.123, -0.065) | <0.001 |
| Total_L | alcohol | 0.106 (0.080, 0.133) | <0.001 |
| XL_VLDL_L | alcohol | -0.122 (-0.151, -0.092) | <0.001 |
| Total_TG | alcohol | -0.075 (-0.104, -0.046) | <0.001 |
| HDL_TG | alcohol | 0.055 (0.028, 0.083) | <0.001 |
| IDL_TG | alcohol | 0.042 (0.014, 0.069) | 0.003 |
| LDL_TG | alcohol | 0.034 (0.006, 0.062) | 0.017 |
| L_LDL_TG | alcohol | 0.063 (0.035, 0.091) | <0.001 |
| M_LDL_TG | alcohol | 0.008 (-0.021, 0.036) | 0.599 |
| M_VLDL_TG | alcohol | -0.105 (-0.134, -0.076) | <0.001 |
| S_HDL_TG | alcohol | -0.025 (-0.054, 0.005) | 0.100 |
| XL_VLDL_TG | alcohol | -0.102 (-0.132, -0.072) | <0.001 |
| L_HDL_TG_pct | alcohol | -0.161 (-0.190, -0.132) | <0.001 |
| M_HDL_TG_pct | alcohol | -0.170 (-0.199, -0.140) | <0.001 |
| M_VLDL_TG_pct | alcohol | -0.033 (-0.062, -0.005) | 0.023 |
| XL_HDL_TG_pct | alcohol | -0.040 (-0.069, -0.011) | 0.007 |

# Supplementary Table 13 Association between DASH diet components and individual metabolites in metabolomic signature

| Metabolite | Dietary component | β (95%CI) | p |
| --- | --- | --- | --- |
| Albumin | vegetables | 0.088 (0.056, 0.120) | <0.001 |
| HDL_size | vegetables | 0.085 (0.044, 0.126) | <0.001 |
| VLDL_size | vegetables | -0.055 (-0.093, -0.017) | 0.005 |
| M_LDL_C | vegetables | -0.035 (-0.068, -0.002) | 0.036 |
| S_HDL_C | vegetables | 0.002 (-0.031, 0.034) | 0.918 |
| XL_HDL_C | vegetables | 0.069 (0.029, 0.108) | <0.001 |
| S_LDL_C_pct | vegetables | -0.058 (-0.090, -0.026) | <0.001 |
| XXL_VLDL_CE | vegetables | -0.024 (-0.059, 0.012) | 0.193 |
| LDL_CE | vegetables | -0.021 (-0.054, 0.011) | 0.204 |
| M_HDL_CE | vegetables | 0.064 (0.027, 0.100) | <0.001 |
| S_HDL_CE | vegetables | -0.006 (-0.038, 0.026) | 0.722 |
| S_LDL_CE | vegetables | -0.031 (-0.064, 0.002) | 0.064 |
| XL_HDL_CE | vegetables | 0.074 (0.034, 0.114) | <0.001 |
| S_LDL_CE_pct | vegetables | -0.037 (-0.070, -0.004) | 0.029 |
| HDL_P | vegetables | 0.045 (0.010, 0.080) | 0.011 |
| M_HDL_P | vegetables | 0.064 (0.028, 0.100) | <0.001 |
| XL_HDL_P | vegetables | 0.069 (0.030, 0.108) | <0.001 |
| Unsaturation | vegetables | 0.159 (0.125, 0.193) | <0.001 |
| DHA | vegetables | 0.191 (0.157, 0.225) | <0.001 |
| DHA_pct | vegetables | 0.193 (0.159, 0.227) | <0.001 |
| XL_HDL_FC | vegetables | 0.045 (0.008, 0.083) | 0.018 |
| S_HDL_FC_pct | vegetables | 0.047 (0.012, 0.082) | 0.009 |
| XL_HDL_FC_pct | vegetables | -0.055 (-0.094, -0.017) | 0.005 |
| GlycA | vegetables | -0.003 (-0.038, 0.032) | 0.859 |
| LA_pct | vegetables | -0.015 (-0.050, 0.020) | 0.398 |
| MUFA | vegetables | 0.001 (-0.033, 0.036) | 0.942 |
| MUFA_pct | vegetables | -0.053 (-0.089, -0.016) | 0.005 |
| Omega_3 | vegetables | 0.183 (0.150, 0.216) | <0.001 |
| Omega_3_pct | vegetables | 0.207 (0.174, 0.240) | <0.001 |
| Omega_6_by_Omega_3 | vegetables | -0.146 (-0.178, -0.113) | <0.001 |
| Omega_6_pct | vegetables | 0.001 (-0.034, 0.036) | 0.974 |
| M_HDL_PL | vegetables | 0.063 (0.028, 0.098) | <0.001 |
| S_HDL_PL | vegetables | 0.015 (-0.018, 0.048) | 0.361 |
| XL_HDL_PL | vegetables | 0.077 (0.038, 0.116) | <0.001 |
| IDL_PL_pct | vegetables | 0.036 (0.004, 0.069) | 0.027 |
| L_HDL_PL_pct | vegetables | -0.001 (-0.037, 0.035) | 0.954 |
| S_LDL_PL_pct | vegetables | 0.040 (0.007, 0.074) | 0.018 |
| PUFA_pct | vegetables | 0.096 (0.061, 0.132) | <0.001 |
| SFA_pct | vegetables | -0.096 (-0.129, -0.063) | <0.001 |
| LDL_L | vegetables | -0.021 (-0.054, 0.012) | 0.205 |
| Total_L | vegetables | 0.004 (-0.028, 0.037) | 0.791 |
| M_LDL_L | vegetables | -0.031 (-0.065, 0.002) | 0.062 |
| XL_HDL_L | vegetables | 0.074 (0.035, 0.113) | <0.001 |
| M_HDL_TG | vegetables | 0.013 (-0.021, 0.046) | 0.450 |
| S_HDL_TG | vegetables | -0.009 (-0.045, 0.026) | 0.604 |
| XL_HDL_TG_pct | vegetables | -0.011 (-0.047, 0.024) | 0.536 |
| Albumin | fruit | 0.046 (0.016, 0.076) | 0.003 |
| HDL_size | fruit | 0.037 (-0.001, 0.076) | 0.054 |
| VLDL_size | fruit | -0.020 (-0.056, 0.015) | 0.263 |
| M_LDL_C | fruit | -0.054 (-0.084, -0.023) | <0.001 |
| S_HDL_C | fruit | -0.136 (-0.166, -0.105) | <0.001 |
| XL_HDL_C | fruit | 0.029 (-0.008, 0.066) | 0.124 |
| S_LDL_C_pct | fruit | -0.082 (-0.112, -0.052) | <0.001 |
| XXL_VLDL_CE | fruit | -0.021 (-0.054, 0.012) | 0.219 |
| LDL_CE | fruit | -0.050 (-0.080, -0.019) | 0.001 |
| M_HDL_CE | fruit | -0.113 (-0.147, -0.079) | <0.001 |
| S_HDL_CE | fruit | -0.141 (-0.171, -0.110) | <0.001 |
| S_LDL_CE | fruit | -0.047 (-0.078, -0.016) | 0.003 |
| XL_HDL_CE | fruit | 0.021 (-0.016, 0.058) | 0.275 |
| S_LDL_CE_pct | fruit | -0.058 (-0.089, -0.026) | <0.001 |
| HDL_P | fruit | -0.121 (-0.153, -0.088) | <0.001 |
| M_HDL_P | fruit | -0.101 (-0.135, -0.067) | <0.001 |
| XL_HDL_P | fruit | 0.032 (-0.005, 0.068) | 0.086 |
| Unsaturation | fruit | 0.121 (0.089, 0.153) | <0.001 |
| DHA | fruit | 0.137 (0.106, 0.169) | <0.001 |
| DHA_pct | fruit | 0.187 (0.155, 0.219) | <0.001 |
| XL_HDL_FC | fruit | 0.055 (0.020, 0.090) | 0.002 |
| S_HDL_FC_pct | fruit | 0.060 (0.027, 0.092) | <0.001 |
| XL_HDL_FC_pct | fruit | 0.004 (-0.032, 0.040) | 0.818 |
| GlycA | fruit | -0.061 (-0.094, -0.028) | <0.001 |
| LA_pct | fruit | -0.008 (-0.041, 0.025) | 0.632 |
| MUFA | fruit | -0.064 (-0.096, -0.031) | <0.001 |
| MUFA_pct | fruit | -0.092 (-0.126, -0.057) | <0.001 |
| Omega_3 | fruit | 0.135 (0.104, 0.167) | <0.001 |
| Omega_3_pct | fruit | 0.201 (0.170, 0.232) | <0.001 |
| Omega_6_by_Omega_3 | fruit | -0.161 (-0.191, -0.130) | <0.001 |
| Omega_6_pct | fruit | -0.004 (-0.037, 0.029) | 0.811 |
| M_HDL_PL | fruit | -0.093 (-0.126, -0.060) | <0.001 |
| S_HDL_PL | fruit | -0.120 (-0.151, -0.089) | <0.001 |
| XL_HDL_PL | fruit | 0.045 (0.008, 0.082) | 0.016 |
| IDL_PL_pct | fruit | 0.094 (0.064, 0.124) | <0.001 |
| L_HDL_PL_pct | fruit | -0.018 (-0.052, 0.016) | 0.292 |
| S_LDL_PL_pct | fruit | 0.059 (0.028, 0.090) | <0.001 |
| PUFA_pct | fruit | 0.089 (0.056, 0.122) | <0.001 |
| SFA_pct | fruit | -0.048 (-0.078, -0.017) | 0.002 |
| LDL_L | fruit | -0.050 (-0.080, -0.019) | 0.001 |
| Total_L | fruit | -0.048 (-0.079, -0.018) | 0.002 |
| M_LDL_L | fruit | -0.053 (-0.084, -0.022) | <0.001 |
| XL_HDL_L | fruit | 0.040 (0.003, 0.076) | 0.035 |
| M_HDL_TG | fruit | 0.001 (-0.030, 0.033) | 0.934 |
| S_HDL_TG | fruit | -0.002 (-0.035, 0.032) | 0.926 |
| XL_HDL_TG_pct | fruit | -0.001 (-0.034, 0.033) | 0.970 |
| Albumin | nuts and legumes | 0.002 (-0.009, 0.012) | 0.757 |
| HDL_size | nuts and legumes | 0.006 (-0.007, 0.018) | 0.392 |
| VLDL_size | nuts and legumes | -0.011 (-0.023, 0.001) | 0.069 |
| M_LDL_C | nuts and legumes | -0.003 (-0.013, 0.008) | 0.626 |
| S_HDL_C | nuts and legumes | -0.004 (-0.014, 0.006) | 0.416 |
| XL_HDL_C | nuts and legumes | 0.009 (-0.003, 0.021) | 0.145 |
| S_LDL_C_pct | nuts and legumes | 0.002 (-0.008, 0.012) | 0.692 |
| XXL_VLDL_CE | nuts and legumes | -0.009 (-0.020, 0.002) | 0.108 |
| LDL_CE | nuts and legumes | -0.002 (-0.012, 0.008) | 0.677 |
| M_HDL_CE | nuts and legumes | -0.001 (-0.013, 0.010) | 0.840 |
| S_HDL_CE | nuts and legumes | -0.002 (-0.012, 0.008) | 0.670 |
| S_LDL_CE | nuts and legumes | -0.001 (-0.011, 0.009) | 0.865 |
| XL_HDL_CE | nuts and legumes | 0.010 (-0.002, 0.023) | 0.097 |
| S_LDL_CE_pct | nuts and legumes | -0.010 (-0.021, 0.000) | 0.050 |
| HDL_P | nuts and legumes | -0.004 (-0.015, 0.007) | 0.494 |
| M_HDL_P | nuts and legumes | -0.006 (-0.017, 0.006) | 0.324 |
| XL_HDL_P | nuts and legumes | 0.005 (-0.007, 0.017) | 0.403 |
| Unsaturation | nuts and legumes | 0.025 (0.014, 0.035) | <0.001 |
| DHA | nuts and legumes | 0.008 (-0.002, 0.019) | 0.122 |
| DHA_pct | nuts and legumes | 0.014 (0.004, 0.025) | 0.008 |
| XL_HDL_FC | nuts and legumes | 0.004 (-0.008, 0.016) | 0.513 |
| S_HDL_FC_pct | nuts and legumes | 0.004 (-0.007, 0.015) | 0.437 |
| XL_HDL_FC_pct | nuts and legumes | -0.002 (-0.014, 0.010) | 0.747 |
| GlycA | nuts and legumes | -0.007 (-0.018, 0.004) | 0.227 |
| LA_pct | nuts and legumes | 0.050 (0.039, 0.061) | <0.001 |
| MUFA | nuts and legumes | -0.007 (-0.018, 0.004) | 0.191 |
| MUFA_pct | nuts and legumes | -0.007 (-0.018, 0.004) | 0.235 |
| Omega_3 | nuts and legumes | 0.000 (-0.010, 0.011) | 0.940 |
| Omega_3_pct | nuts and legumes | 0.005 (-0.006, 0.015) | 0.368 |
| Omega_6_by_Omega_3 | nuts and legumes | 0.011 (0.001, 0.021) | 0.037 |
| Omega_6_pct | nuts and legumes | 0.039 (0.028, 0.050) | <0.001 |
| M_HDL_PL | nuts and legumes | -0.010 (-0.021, 0.001) | 0.085 |
| S_HDL_PL | nuts and legumes | -0.013 (-0.023, -0.003) | 0.013 |
| XL_HDL_PL | nuts and legumes | 0.006 (-0.006, 0.018) | 0.332 |
| IDL_PL_pct | nuts and legumes | 0.011 (0.000, 0.021) | 0.040 |
| L_HDL_PL_pct | nuts and legumes | -0.016 (-0.028, -0.005) | 0.004 |
| S_LDL_PL_pct | nuts and legumes | 0.011 (0.001, 0.022) | 0.035 |
| PUFA_pct | nuts and legumes | 0.040 (0.029, 0.051) | <0.001 |
| SFA_pct | nuts and legumes | -0.043 (-0.053, -0.033) | <0.001 |
| LDL_L | nuts and legumes | -0.002 (-0.012, 0.008) | 0.656 |
| Total_L | nuts and legumes | -0.008 (-0.018, 0.003) | 0.143 |
| M_LDL_L | nuts and legumes | -0.003 (-0.013, 0.008) | 0.606 |
| XL_HDL_L | nuts and legumes | 0.007 (-0.005, 0.019) | 0.278 |
| M_HDL_TG | nuts and legumes | -0.023 (-0.033, -0.012) | <0.001 |
| S_HDL_TG | nuts and legumes | -0.020 (-0.031, -0.008) | <0.001 |
| XL_HDL_TG_pct | nuts and legumes | -0.001 (-0.013, 0.010) | 0.791 |
| Albumin | whole grains | 0.014 (-0.011, 0.039) | 0.275 |
| HDL_size | whole grains | 0.029 (-0.002, 0.061) | 0.070 |
| VLDL_size | whole grains | -0.009 (-0.039, 0.021) | 0.549 |
| M_LDL_C | whole grains | -0.042 (-0.068, -0.017) | 0.001 |
| S_HDL_C | whole grains | -0.085 (-0.110, -0.060) | <0.001 |
| XL_HDL_C | whole grains | 0.026 (-0.004, 0.057) | 0.094 |
| S_LDL_C_pct | whole grains | -0.038 (-0.063, -0.013) | 0.003 |
| XXL_VLDL_CE | whole grains | -0.015 (-0.043, 0.013) | 0.284 |
| LDL_CE | whole grains | -0.042 (-0.068, -0.017) | 0.001 |
| M_HDL_CE | whole grains | -0.065 (-0.093, -0.036) | <0.001 |
| S_HDL_CE | whole grains | -0.084 (-0.109, -0.059) | <0.001 |
| S_LDL_CE | whole grains | -0.033 (-0.059, -0.008) | 0.011 |
| XL_HDL_CE | whole grains | 0.024 (-0.007, 0.055) | 0.122 |
| S_LDL_CE_pct | whole grains | -0.045 (-0.071, -0.019) | <0.001 |
| HDL_P | whole grains | -0.075 (-0.103, -0.048) | <0.001 |
| M_HDL_P | whole grains | -0.065 (-0.093, -0.037) | <0.001 |
| XL_HDL_P | whole grains | 0.022 (-0.008, 0.053) | 0.147 |
| Unsaturation | whole grains | 0.084 (0.058, 0.111) | <0.001 |
| DHA | whole grains | 0.037 (0.010, 0.063) | 0.006 |
| DHA_pct | whole grains | 0.068 (0.041, 0.094) | <0.001 |
| XL_HDL_FC | whole grains | 0.031 (0.001, 0.060) | 0.040 |
| S_HDL_FC_pct | whole grains | 0.022 (-0.005, 0.050) | 0.109 |
| XL_HDL_FC_pct | whole grains | -0.028 (-0.058, 0.002) | 0.067 |
| GlycA | whole grains | -0.009 (-0.037, 0.018) | 0.503 |
| LA_pct | whole grains | 0.116 (0.089, 0.143) | <0.001 |
| MUFA | whole grains | -0.044 (-0.071, -0.017) | 0.001 |
| MUFA_pct | whole grains | -0.047 (-0.075, -0.018) | 0.001 |
| Omega_3 | whole grains | 0.036 (0.010, 0.062) | 0.007 |
| Omega_3_pct | whole grains | 0.069 (0.043, 0.095) | <0.001 |
| Omega_6_by_Omega_3 | whole grains | -0.035 (-0.061, -0.009) | 0.007 |
| Omega_6_pct | whole grains | 0.082 (0.055, 0.110) | <0.001 |
| M_HDL_PL | whole grains | -0.066 (-0.094, -0.039) | <0.001 |
| S_HDL_PL | whole grains | -0.086 (-0.111, -0.060) | <0.001 |
| XL_HDL_PL | whole grains | 0.033 (0.002, 0.064) | 0.035 |
| IDL_PL_pct | whole grains | 0.041 (0.016, 0.066) | 0.001 |
| L_HDL_PL_pct | whole grains | -0.025 (-0.053, 0.003) | 0.082 |
| S_LDL_PL_pct | whole grains | 0.043 (0.017, 0.069) | 0.001 |
| PUFA_pct | whole grains | 0.112 (0.085, 0.140) | <0.001 |
| SFA_pct | whole grains | -0.105 (-0.131, -0.080) | <0.001 |
| LDL_L | whole grains | -0.042 (-0.068, -0.017) | 0.001 |
| Total_L | whole grains | -0.044 (-0.069, -0.018) | <0.001 |
| M_LDL_L | whole grains | -0.042 (-0.068, -0.017) | 0.001 |
| XL_HDL_L | whole grains | 0.030 (-0.001, 0.061) | 0.056 |
| M_HDL_TG | whole grains | -0.033 (-0.059, -0.007) | 0.014 |
| S_HDL_TG | whole grains | -0.029 (-0.057, -0.001) | 0.041 |
| XL_HDL_TG_pct | whole grains | -0.028 (-0.055, 0.000) | 0.050 |
| Albumin | sugary drinks | -0.018 (-0.032, -0.005) | 0.008 |
| HDL_size | sugary drinks | -0.020 (-0.037, -0.002) | 0.025 |
| VLDL_size | sugary drinks | 0.001 (-0.015, 0.017) | 0.866 |
| M_LDL_C | sugary drinks | -0.016 (-0.030, -0.002) | 0.023 |
| S_HDL_C | sugary drinks | 0.001 (-0.012, 0.015) | 0.866 |
| XL_HDL_C | sugary drinks | -0.028 (-0.044, -0.011) | 0.001 |
| S_LDL_C_pct | sugary drinks | -0.019 (-0.032, -0.005) | 0.006 |
| XXL_VLDL_CE | sugary drinks | 0.008 (-0.007, 0.023) | 0.281 |
| LDL_CE | sugary drinks | -0.017 (-0.031, -0.004) | 0.013 |
| M_HDL_CE | sugary drinks | -0.005 (-0.020, 0.010) | 0.514 |
| S_HDL_CE | sugary drinks | 0.001 (-0.013, 0.015) | 0.891 |
| S_LDL_CE | sugary drinks | -0.016 (-0.030, -0.002) | 0.026 |
| XL_HDL_CE | sugary drinks | -0.028 (-0.045, -0.011) | 0.001 |
| S_LDL_CE_pct | sugary drinks | -0.001 (-0.015, 0.013) | 0.926 |
| HDL_P | sugary drinks | -0.006 (-0.021, 0.008) | 0.392 |
| M_HDL_P | sugary drinks | -0.004 (-0.019, 0.012) | 0.652 |
| XL_HDL_P | sugary drinks | -0.025 (-0.041, -0.009) | 0.003 |
| Unsaturation | sugary drinks | -0.023 (-0.037, -0.009) | 0.002 |
| DHA | sugary drinks | -0.020 (-0.035, -0.006) | 0.005 |
| DHA_pct | sugary drinks | -0.020 (-0.034, -0.005) | 0.007 |
| XL_HDL_FC | sugary drinks | -0.025 (-0.041, -0.009) | 0.002 |
| S_HDL_FC_pct | sugary drinks | -0.018 (-0.033, -0.003) | 0.018 |
| XL_HDL_FC_pct | sugary drinks | 0.021 (0.005, 0.037) | 0.012 |
| GlycA | sugary drinks | 0.007 (-0.008, 0.022) | 0.355 |
| LA_pct | sugary drinks | -0.036 (-0.050, -0.021) | <0.001 |
| MUFA | sugary drinks | 0.010 (-0.005, 0.024) | 0.191 |
| MUFA_pct | sugary drinks | 0.026 (0.011, 0.042) | <0.001 |
| Omega_3 | sugary drinks | -0.015 (-0.029, -0.001) | 0.040 |
| Omega_3_pct | sugary drinks | -0.017 (-0.031, -0.003) | 0.014 |
| Omega_6_by_Omega_3 | sugary drinks | 0.022 (0.008, 0.036) | 0.002 |
| Omega_6_pct | sugary drinks | -0.019 (-0.034, -0.005) | 0.010 |
| M_HDL_PL | sugary drinks | 0.003 (-0.012, 0.017) | 0.737 |
| S_HDL_PL | sugary drinks | 0.009 (-0.005, 0.023) | 0.203 |
| XL_HDL_PL | sugary drinks | -0.023 (-0.039, -0.006) | 0.007 |
| IDL_PL_pct | sugary drinks | 0.005 (-0.008, 0.019) | 0.452 |
| L_HDL_PL_pct | sugary drinks | 0.039 (0.024, 0.054) | <0.001 |
| S_LDL_PL_pct | sugary drinks | 0.001 (-0.013, 0.015) | 0.900 |
| PUFA_pct | sugary drinks | -0.027 (-0.042, -0.012) | <0.001 |
| SFA_pct | sugary drinks | 0.014 (0.000, 0.028) | 0.043 |
| LDL_L | sugary drinks | -0.017 (-0.031, -0.003) | 0.015 |
| Total_L | sugary drinks | -0.014 (-0.028, 0.000) | 0.051 |
| M_LDL_L | sugary drinks | -0.014 (-0.028, 0.000) | 0.046 |
| XL_HDL_L | sugary drinks | -0.025 (-0.041, -0.008) | 0.004 |
| M_HDL_TG | sugary drinks | 0.016 (0.002, 0.030) | 0.027 |
| S_HDL_TG | sugary drinks | 0.020 (0.005, 0.035) | 0.008 |
| XL_HDL_TG_pct | sugary drinks | 0.022 (0.007, 0.037) | 0.004 |
| Albumin | low-fat dairy | -0.006 (-0.016, 0.004) | 0.254 |
| HDL_size | low-fat dairy | -0.004 (-0.017, 0.009) | 0.574 |
| VLDL_size | low-fat dairy | -0.004 (-0.017, 0.008) | 0.469 |
| M_LDL_C | low-fat dairy | -0.024 (-0.034, -0.013) | <0.001 |
| S_HDL_C | low-fat dairy | -0.046 (-0.056, -0.035) | <0.001 |
| XL_HDL_C | low-fat dairy | -0.002 (-0.015, 0.010) | 0.728 |
| S_LDL_C_pct | low-fat dairy | -0.024 (-0.034, -0.014) | <0.001 |
| XXL_VLDL_CE | low-fat dairy | -0.004 (-0.015, 0.008) | 0.543 |
| LDL_CE | low-fat dairy | -0.026 (-0.036, -0.015) | <0.001 |
| M_HDL_CE | low-fat dairy | -0.041 (-0.053, -0.030) | <0.001 |
| S_HDL_CE | low-fat dairy | -0.046 (-0.056, -0.035) | <0.001 |
| S_LDL_CE | low-fat dairy | -0.022 (-0.033, -0.012) | <0.001 |
| XL_HDL_CE | low-fat dairy | -0.006 (-0.018, 0.007) | 0.387 |
| S_LDL_CE_pct | low-fat dairy | -0.018 (-0.029, -0.007) | <0.001 |
| HDL_P | low-fat dairy | -0.046 (-0.057, -0.035) | <0.001 |
| M_HDL_P | low-fat dairy | -0.038 (-0.050, -0.026) | <0.001 |
| XL_HDL_P | low-fat dairy | -0.004 (-0.016, 0.009) | 0.580 |
| Unsaturation | low-fat dairy | 0.004 (-0.007, 0.015) | 0.459 |
| DHA | low-fat dairy | -0.002 (-0.013, 0.009) | 0.685 |
| DHA_pct | low-fat dairy | 0.011 (0.000, 0.022) | 0.044 |
| XL_HDL_FC | low-fat dairy | 0.009 (-0.003, 0.021) | 0.123 |
| S_HDL_FC_pct | low-fat dairy | -0.002 (-0.014, 0.009) | 0.682 |
| XL_HDL_FC_pct | low-fat dairy | 0.008 (-0.004, 0.020) | 0.210 |
| GlycA | low-fat dairy | 0.003 (-0.008, 0.015) | 0.557 |
| LA_pct | low-fat dairy | -0.028 (-0.039, -0.016) | <0.001 |
| MUFA | low-fat dairy | -0.023 (-0.034, -0.012) | <0.001 |
| MUFA_pct | low-fat dairy | -0.013 (-0.025, -0.001) | 0.031 |
| Omega_3 | low-fat dairy | 0.002 (-0.008, 0.013) | 0.651 |
| Omega_3_pct | low-fat dairy | 0.017 (0.006, 0.027) | 0.002 |
| Omega_6_by_Omega_3 | low-fat dairy | -0.025 (-0.035, -0.014) | <0.001 |
| Omega_6_pct | low-fat dairy | -0.011 (-0.022, 0.001) | 0.065 |
| M_HDL_PL | low-fat dairy | -0.032 (-0.044, -0.021) | <0.001 |
| S_HDL_PL | low-fat dairy | -0.037 (-0.047, -0.026) | <0.001 |
| XL_HDL_PL | low-fat dairy | 0.005 (-0.008, 0.017) | 0.476 |
| IDL_PL_pct | low-fat dairy | 0.025 (0.014, 0.035) | <0.001 |
| L_HDL_PL_pct | low-fat dairy | 0.012 (0.000, 0.023) | 0.045 |
| S_LDL_PL_pct | low-fat dairy | 0.020 (0.010, 0.031) | <0.001 |
| PUFA_pct | low-fat dairy | -0.003 (-0.014, 0.009) | 0.654 |
| SFA_pct | low-fat dairy | 0.016 (0.005, 0.026) | 0.003 |
| LDL_L | low-fat dairy | -0.025 (-0.036, -0.015) | <0.001 |
| Total_L | low-fat dairy | -0.024 (-0.035, -0.014) | <0.001 |
| M_LDL_L | low-fat dairy | -0.024 (-0.035, -0.014) | <0.001 |
| XL_HDL_L | low-fat dairy | 0.002 (-0.011, 0.014) | 0.803 |
| M_HDL_TG | low-fat dairy | -0.001 (-0.011, 0.010) | 0.908 |
| S_HDL_TG | low-fat dairy | -0.001 (-0.012, 0.011) | 0.910 |
| XL_HDL_TG_pct | low-fat dairy | -0.007 (-0.018, 0.004) | 0.220 |
| Albumin | sodium | -5.874 (-14.654, 2.905) | 0.190 |
| HDL_size | sodium | -9.593 (-20.614, 1.429) | 0.088 |
| VLDL_size | sodium | 11.736 (1.458, 22.013) | 0.025 |
| M_LDL_C | sodium | 7.184 (-1.743, 16.112) | 0.115 |
| S_HDL_C | sodium | 3.193 (-5.590, 11.976) | 0.476 |
| XL_HDL_C | sodium | -3.461 (-14.112, 7.191) | 0.524 |
| S_LDL_C_pct | sodium | 3.595 (-5.115, 12.305) | 0.418 |
| XXL_VLDL_CE | sodium | 10.937 (1.312, 20.562) | 0.026 |
| LDL_CE | sodium | 6.035 (-2.798, 14.868) | 0.181 |
| M_HDL_CE | sodium | -3.597 (-13.477, 6.284) | 0.476 |
| S_HDL_CE | sodium | 3.739 (-5.027, 12.505) | 0.403 |
| S_LDL_CE | sodium | 9.090 (0.125, 18.055) | 0.047 |
| XL_HDL_CE | sodium | -2.896 (-13.649, 7.857) | 0.598 |
| S_LDL_CE_pct | sodium | 7.056 (-1.984, 16.095) | 0.126 |
| HDL_P | sodium | -0.270 (-9.679, 9.139) | 0.955 |
| M_HDL_P | sodium | -4.006 (-13.797, 5.784) | 0.422 |
| XL_HDL_P | sodium | -3.166 (-13.677, 7.346) | 0.555 |
| Unsaturation | sodium | 6.317 (-2.948, 15.583) | 0.181 |
| DHA | sodium | -4.263 (-13.409, 4.883) | 0.361 |
| DHA_pct | sodium | -10.980 (-20.194, -1.766) | 0.020 |
| XL_HDL_FC | sodium | -5.170 (-15.307, 4.966) | 0.317 |
| S_HDL_FC_pct | sodium | -2.623 (-12.123, 6.877) | 0.588 |
| XL_HDL_FC_pct | sodium | 9.945 (-0.478, 20.368) | 0.061 |
| GlycA | sodium | 15.061 (5.568, 24.555) | 0.002 |
| LA_pct | sodium | 12.051 (2.648, 21.454) | 0.012 |
| MUFA | sodium | 12.486 (3.199, 21.772) | 0.008 |
| MUFA_pct | sodium | 14.704 (4.813, 24.594) | 0.004 |
| Omega_3 | sodium | 2.313 (-6.720, 11.346) | 0.616 |
| Omega_3_pct | sodium | -3.790 (-12.792, 5.211) | 0.409 |
| Omega_6_by_Omega_3 | sodium | 2.770 (-6.142, 11.682) | 0.542 |
| Omega_6_pct | sodium | 7.330 (-2.175, 16.835) | 0.131 |
| M_HDL_PL | sodium | -4.964 (-14.478, 4.549) | 0.306 |
| S_HDL_PL | sodium | 0.743 (-8.162, 9.647) | 0.870 |
| XL_HDL_PL | sodium | -4.844 (-15.503, 5.815) | 0.373 |
| IDL_PL_pct | sodium | 1.463 (-7.250, 10.177) | 0.742 |
| L_HDL_PL_pct | sodium | -1.463 (-11.230, 8.303) | 0.769 |
| S_LDL_PL_pct | sodium | -5.664 (-14.720, 3.392) | 0.220 |
| PUFA_pct | sodium | 5.391 (-4.189, 14.970) | 0.270 |
| SFA_pct | sodium | -20.694 (-29.527, -11.862) | <0.001 |
| LDL_L | sodium | 5.479 (-3.352, 14.310) | 0.224 |
| Total_L | sodium | 4.623 (-4.285, 13.530) | 0.309 |
| M_LDL_L | sodium | 7.442 (-1.515, 16.398) | 0.103 |
| XL_HDL_L | sodium | -4.182 (-14.823, 6.458) | 0.441 |
| M_HDL_TG | sodium | -2.199 (-11.245, 6.848) | 0.634 |
| S_HDL_TG | sodium | 3.431 (-6.279, 13.141) | 0.489 |
| XL_HDL_TG_pct | sodium | 7.510 (-2.107, 17.127) | 0.126 |
| Albumin | red and processed meat | 0.008 (-0.007, 0.022) | 0.298 |
| HDL_size | red and processed meat | -0.015 (-0.033, 0.003) | 0.113 |
| VLDL_size | red and processed meat | 0.020 (0.003, 0.037) | 0.021 |
| M_LDL_C | red and processed meat | 0.015 (0.000, 0.030) | 0.043 |
| S_HDL_C | red and processed meat | 0.034 (0.019, 0.048) | <0.001 |
| XL_HDL_C | red and processed meat | -0.021 (-0.038, -0.003) | 0.021 |
| S_LDL_C_pct | red and processed meat | 0.020 (0.006, 0.035) | 0.006 |
| XXL_VLDL_CE | red and processed meat | 0.016 (0.000, 0.032) | 0.046 |
| LDL_CE | red and processed meat | 0.014 (0.000, 0.029) | 0.058 |
| M_HDL_CE | red and processed meat | 0.025 (0.009, 0.041) | 0.003 |
| S_HDL_CE | red and processed meat | 0.032 (0.018, 0.047) | <0.001 |
| S_LDL_CE | red and processed meat | 0.012 (-0.003, 0.027) | 0.116 |
| XL_HDL_CE | red and processed meat | -0.020 (-0.038, -0.002) | 0.026 |
| S_LDL_CE_pct | red and processed meat | 0.029 (0.014, 0.044) | <0.001 |
| HDL_P | red and processed meat | 0.031 (0.015, 0.046) | <0.001 |
| M_HDL_P | red and processed meat | 0.030 (0.014, 0.046) | <0.001 |
| XL_HDL_P | red and processed meat | -0.017 (-0.035, 0.000) | 0.050 |
| Unsaturation | red and processed meat | 0.000 (-0.016, 0.015) | 0.965 |
| DHA | red and processed meat | 0.005 (-0.010, 0.021) | 0.476 |
| DHA_pct | red and processed meat | -0.012 (-0.027, 0.004) | 0.138 |
| XL_HDL_FC | red and processed meat | -0.021 (-0.038, -0.004) | 0.013 |
| S_HDL_FC_pct | red and processed meat | -0.024 (-0.039, -0.008) | 0.003 |
| XL_HDL_FC_pct | red and processed meat | 0.019 (0.002, 0.037) | 0.027 |
| GlycA | red and processed meat | 0.018 (0.003, 0.034) | 0.022 |
| LA_pct | red and processed meat | -0.064 (-0.079, -0.048) | <0.001 |
| MUFA | red and processed meat | 0.025 (0.010, 0.040) | 0.001 |
| MUFA_pct | red and processed meat | 0.014 (-0.003, 0.030) | 0.098 |
| Omega_3 | red and processed meat | 0.021 (0.006, 0.036) | 0.005 |
| Omega_3_pct | red and processed meat | 0.009 (-0.006, 0.023) | 0.260 |
| Omega_6_by_Omega_3 | red and processed meat | -0.031 (-0.045, -0.016) | <0.001 |
| Omega_6_pct | red and processed meat | -0.039 (-0.055, -0.023) | <0.001 |
| M_HDL_PL | red and processed meat | 0.035 (0.019, 0.051) | <0.001 |
| S_HDL_PL | red and processed meat | 0.045 (0.030, 0.060) | <0.001 |
| XL_HDL_PL | red and processed meat | -0.018 (-0.035, 0.000) | 0.046 |
| IDL_PL_pct | red and processed meat | -0.028 (-0.042, -0.013) | <0.001 |
| L_HDL_PL_pct | red and processed meat | 0.042 (0.026, 0.058) | <0.001 |
| S_LDL_PL_pct | red and processed meat | -0.027 (-0.042, -0.013) | <0.001 |
| PUFA_pct | red and processed meat | -0.034 (-0.050, -0.018) | <0.001 |
| SFA_pct | red and processed meat | 0.025 (0.010, 0.040) | <0.001 |
| LDL_L | red and processed meat | 0.013 (-0.001, 0.028) | 0.075 |
| Total_L | red and processed meat | 0.022 (0.008, 0.037) | 0.003 |
| M_LDL_L | red and processed meat | 0.015 (0.000, 0.030) | 0.050 |
| XL_HDL_L | red and processed meat | -0.019 (-0.036, -0.001) | 0.036 |
| M_HDL_TG | red and processed meat | 0.030 (0.015, 0.045) | <0.001 |
| S_HDL_TG | red and processed meat | 0.025 (0.009, 0.041) | 0.002 |
| XL_HDL_TG_pct | red and processed meat | 0.016 (0.000, 0.032) | 0.050 |

# Supplementary Table 14 Mediation analysis of metabolomic signatures in the dietary-BAG association

|  | Estimate | 95%CI | P |
| --- | --- | --- | --- |
| **DASH** | | | |
| Indirect effect | -0.0467 | -0.0637, -0.0304 | <0.001 |
| Direct effect | -0.0850 | -0.1683, -0.0002 | 0.054 |
| Total effect | -0.1317 | -0.2117, -0.0452 | <0.001 |
| Proportion | 0.3547 | 0.1876, 1.0049 | <0.001 |
| **AHEI-2010** | | | |
| Indirect effect | -0.0637 | -0.0871, -0.0404 | <0.001 |
| Direct effect | -0.1456 | -0.2359, -0.0568 | 0.004 |
| Total effect | -0.2093 | -0.2929, -0.1221 | <0.001 |
| Proportion | 0.3043 | 0.1676, 0.5412 | <0.001 |

# Supplementary Table 15 Subgroup analysis of association between AHEI-2010 diet and BAG (per SD Increase in dietary score)

| Characteristics | Counts | Percent | β (95%CI） | P | P for interaction |
| --- | --- | --- | --- | --- | --- |
| age |  |  |  |  | 0.265 |
| ≤55 | 6299 | 48.04% | -0.277 (-0.390, -0.164) | <0.001 |  |
| >55 | 6813 | 51.96% | -0.180 (-0.304, -0.056) | 0.004 |  |
| Sex |  |  |  |  | 0.115 |
| Female | 7348 | 53.67% | -0.195 (-0.309, -0.081) | 0.001 |  |
| Male | 6343 | 46.33% | -0.220 (-0.344, -0.095) | 0.001 |  |
| BMI |  |  |  |  | 0.582 |
| <25 | 5710 | 41.71% | -0.209 (-0.333, -0.085) | 0.001 |  |
| 25-30 | 5753 | 42.02% | -0.229 (-0.360, -0.085) | 0.001 |  |
| ≥30 | 2228 | 16.27% | -0.140 (-0.368, 0.088) | 0.228 |  |
| Alcohol consumption |  |  |  |  | 0.837 |
| Yes | 13108 | 95.74% | -0.210 (-0.295, -0.124) | <0.001 |  |
| No | 583 | 4.26% | -0.233 (-0.645, 0.180) | 0.269 |  |
| Cardiovascular disease |  |  |  |  | 0.911 |
| Yes | 303 | 2.21% | -0.159 (-0.847, 0.529) | 0.649 |  |
| No | 13388 | 97.79% | -0.210 (-0.294, -0.126) | <0.001 |  |
| Diabetes |  |  |  |  | 0.106 |
| Yes | 922 | 6.73% | -0.404 (-0.753, -0.055) | 0.023 |  |
| No | 12769 | 93.27% | -0.197 (-0.284, -0.111) | <0.001 |  |

# Supplementary Table 16 Subgroup analysis of association between DASH diet and BAG (per SD Increase in dietary score)

| Variable | Counts | Percent | β (95%CI) | P | P for interaction |
| --- | --- | --- | --- | --- | --- |
| age |  |  |  |  | 0.854 |
| ≤55 | 6299 | 48.04% | -0.162 (-0.274, -0.049) | 0.005 |  |
| >55 | 6813 | 51.96% | -0.167 (-0.296, -0.038) | 0.011 |  |
| Sex |  |  |  |  | 0.157 |
| Female | 7348 | 53.67% | -0.139 (-0.257, -0.020) | 0.022 |  |
| Male | 6343 | 46.33% | -0.133 (-0.257, -0.009) | 0.035 |  |
| BMI |  |  |  |  | 0.012 |
| <25 | 5710 | 41.71% | -0.088 (-0.217, 0.041) | 0.182 |  |
| 25-30 | 5753 | 42.02% | -0.128 (-0.260, 0.003) | 0.056 |  |
| ≥30 | 2228 | 16.27% | -0.281 (-0.508, -0.055) | 0.015 |  |
| Alcohol consumption |  |  |  |  | 0.417 |
| Yes | 13108 | 95.74% | -0.134 (-0.222, -0.047) | 0.003 |  |
| No | 583 | 4.26% | -0.221 (-0.637, 0.194) | 0.296 |  |
| Cardiovascular disease |  |  |  |  | 0.212 |
| Yes | 303 | 2.21% | -0.478 (-1.168, 0.213) | 0.174 |  |
| No | 13388 | 97.79% | -0.130 (-0.216, -0.044) | 0.003 |  |
| Diabetes |  |  |  |  | 0.327 |
| Yes | 922 | 6.73% | -0.228 (-0.592, 0.136) | 0.220 |  |
| No | 12769 | 93.27% | -0.135 (-0.223, -0.048) | 0.003 |  |

# Supplementary Table 17 Sensitivity analysis: further adjustment for additional covariates (diabetes, cardiovascular disease, and lipid-lowering medication use)

| Dietary pattern | β (95%CI) | P |
| --- | --- | --- |
| **MED** |  |  |
| Low | Reference | - |
| Medium | 0.021 (-0.171, 0.213) | 0.830 |
| High | 0.263 (0.049, 0.477) | 0.016 |
| **DASH** |  |  |
| Low | Reference | - |
| Medium | -0.219 (-0.421, -0.018) | 0.033 |
| High | -0.266 (-0.469, -0.062) | 0.010 |
| **MIND** |  |  |
| Low | Reference | - |
| Medium | -0.011 (-0.205, 0.183) | 0.913 |
| High | -0.003 (-0.216, 0.210) | 0.978 |
| **AHEI-2010** |  |  |
| Low | Reference | - |
| Medium | -0.370 (-0.569, -0.170) | <0.001 |
| High | -0.462 (-0.666, -0.259) | <0.001 |
| **e-DII** |  |  |
| Low | Reference | - |
| Medium | -0.101 (-0.303, 0.100) | 0.324 |
| High | -0.115 (-0.326, 0.095) | 0.283 |

# Supplementary Table 18 Sensitivity analysis: association of metabolomic signatures and BAG in individuals with complete metabolomic data but missing dietary data

|  | **AHEI-2010** | | **DASH** | |
| --- | --- | --- | --- | --- |
|  | β (95%CI) | P | β (95%CI) | P |
| Model 1 | -0.496 (-0.560, -0.432) | <0.001 | -0.476 (-0.540, -0.412) | <0.001 |
| Model 2 | -0.260 (-0.379, -0.142) | <0.001 | -0.299 (-0.393, -0.206) | <0.001 |
| Model 3 | -0.235 (-0.373, -0.098) | <0.001 | -0.302 (-0.396, -0.208) | <0.001 |

# Supplementary Table 19 The association between MED and BAG after removing the score of wine intake

| Dietary pattern | Model 1 | P | Model 2 | P | Model 3 | P |
| --- | --- | --- | --- | --- | --- | --- |
| **MED** | | | | | | |
| Low | Reference | - | Reference | - | Reference | - |
| Medium | -0.055 (-0.237, 0.238) | 0.556 | 0.065 (-0.118, 0.249) | 0.485 | 0.057 (-0.126, 0.241) | 0.540 |
| High | -0.062 (-0.284, 0.160) | 0.585 | 0.141 (-0.084, 0.366) | 0.218 | 0.122 (-0.104, 0.348) | 0.291 |
| **< 7 wine drinks per day** | | | | | | |
| Low | Reference | - | Reference | - | Reference | - |
| Medium | -0.203 (-0.433,0.027) | 0.083 | -0.050 (-0.280,0.180) | 0.668 | -0.063 (-0.294,0.167) | 0.591 |
| High | -0.285 (-0.571,0.000) | 0.050 | -0.015 (-0.304,0.273) | 0.917 | -0.048 (-0.338,0.242) | 0.745 |
| **≥7 wine drinks per day** | | | | | | |
| Low | Reference | - | Reference | - | Reference | - |
| Medium | 0.121 (-0.181,0.424) | 0.432 | 0.203 (-0.100,0.507) | 0.189 | 0.212 (-0.093,0.516) | 0.173 |
| High | 0.192 (-0.164,0.549) | 0.291 | 0.322 (-0.038,0.682) | 0.080 | 0.342 (-0.021,0.704) | 0.065 |

# Supplementary Table 20 Correspondence table of metabolite full names and abbreviations

| Metabolite | Shortened name | Category |
| --- | --- | --- |
| Alanine | Ala | Amino acids |
| Glutamine | Gln | Amino acids |
| Glycine | Gly | Amino acids |
| Histidine | His | Amino acids |
| Total Concentration of Branched Chain Amino Acids Leucine Isoleucine Valine | Total_BCAA | Amino acids |
| Isoleucine | Ile | Amino acids |
| Leucine | Leu | Amino acids |
| Valine | Val | Amino acids |
| Phenylalanine | Phe | Amino acids |
| Tyrosine | Tyr | Amino acids |
| Spectrometer corrected alanine | SCA | Amino acids |
| Glucose lactate | GL | Carbohydrates and carbohydrate metabolism intermediates |
| Glucose | Glucose | Carbohydrates and carbohydrate metabolism intermediates |
| Lactate | Lactate | Carbohydrates and carbohydrate metabolism intermediates |
| Pyruvate | Pyruvate | Carbohydrates and carbohydrate metabolism intermediates |
| Citrate | Citrate | Carbohydrates and carbohydrate metabolism intermediates |
| Total Fatty Acids | Total_FA | Fatty acids |
| Degree of Unsaturation | Unsaturation | Fatty acids |
| Omega 3 Fatty Acids | Omega_3 | Fatty acids |
| Omega 6 Fatty Acids | Omega_6 | Fatty acids |
| Polyunsaturated Fatty Acids | PUFA | Fatty acids |
| Monounsaturated Fatty Acids | MUFA | Fatty acids |
| Saturated Fatty Acids | SFA | Fatty acids |
| Linoleic Acid | LA | Fatty acids |
| Docosahexaenoic Acid | DHA | Fatty acids |
| Omega 3 Fatty Acids to Total Fatty Acids percentage | Omega_3_pct | Fatty acids |
| Omega 6 Fatty Acids to Total Fatty Acids percentage | Omega_6_pct | Fatty acids |
| Polyunsaturated Fatty Acids to Total Fatty Acids percentage | PUFA_pct | Fatty acids |
| Monounsaturated Fatty Acids to Total Fatty Acids percentage | MUFA_pct | Fatty acids |
| Saturated Fatty Acids to Total Fatty Acids percentage | SFA_pct | Fatty acids |
| Linoleic Acid to Total Fatty Acids percentage | LA_pct | Fatty acids |
| Docosahexaenoic Acid to Total Fatty Acids percentage | DHA_pct | Fatty acids |
| Polyunsaturated Fatty Acids to Monounsaturated Fatty Acids ratio | PUFA_by_MUFA | Fatty acids |
| Omega 6 Fatty Acids to Omega 3 Fatty Acids ratio | Omega_6_by_Omega_3 | Fatty acids |
| Total Cholesterol | Total_C | Lipoprotein |
| Total Cholesterol Minus HDL C | non_HDL_C | Lipoprotein |
| Remnant Cholesterol Non HDL, Non LDL Cholesterol | Remnant_C | Lipoprotein |
| VLDL Cholesterol | VLDL_C | Lipoprotein |
| Clinical LDL Cholesterol | Clinical_LDL_C | Lipoprotein |
| LDL Cholesterol | LDL_C | Lipoprotein |
| HDL Cholesterol | HDL_C | Lipoprotein |
| Total Triglycerides | Total_TG | Lipoprotein |
| Triglycerides in VLDL | VLDL_TG | Lipoprotein |
| Triglycerides in LDL | LDL_TG | Lipoprotein |
| Triglycerides in HDL | HDL_TG | Lipoprotein |
| Total Esterified Cholesterol | Total_CE | Lipoprotein |
| Cholesteryl Esters in VLDL | VLDL_CE | Lipoprotein |
| Cholesteryl Esters in LDL | LDL_CE | Lipoprotein |
| Cholesteryl Esters in HDL | HDL_CE | Lipoprotein |
| Total Free Cholesterol | Total_FC | Lipoprotein |
| Free Cholesterol in VLDL | VLDL_FC | Lipoprotein |
| Free Cholesterol in LDL | LDL_FC | Lipoprotein |
| Free Cholesterol in HDL | HDL_FC | Lipoprotein |
| Total Lipids in Lipoprotein Particles | Total_L | Lipoprotein |
| Total Lipids in VLDL | VLDL_L | Lipoprotein |
| Total Lipids in LDL | LDL_L | Lipoprotein |
| Total Lipids in HDL | HDL_L | Lipoprotein |
| Total Concentration of Lipoprotein Particles | Total_P | Lipoprotein |
| Concentration of VLDL Particles | VLDL_P | Lipoprotein |
| Concentration of LDL Particles | LDL_P | Lipoprotein |
| Concentration of HDL Particles | HDL_P | Lipoprotein |
| Average Diameter for VLDL Particles | VLDL_size | Lipoprotein |
| Average Diameter for LDL Particles | LDL_size | Lipoprotein |
| Average Diameter for HDL Particles | HDL_size | Lipoprotein |
| Triglycerides to Phosphoglycerides ratio | TG_by_PG | Lipoprotein |
| Concentration of Chylomicrons and Extremely Large VLDL Particles | XXL_VLDL_P | Lipoprotein |
| Total Lipids in Chylomicrons and Extremely Large VLDL | XXL_VLDL_L | Lipoprotein |
| Cholesterol in Chylomicrons and Extremely Large VLDL | XXL_VLDL_C | Lipoprotein |
| Cholesteryl Esters in Chylomicrons and Extremely Large VLDL | XXL_VLDL_CE | Lipoprotein |
| Free Cholesterol in Chylomicrons and Extremely Large VLDL | XXL_VLDL_FC | Lipoprotein |
| Triglycerides in Chylomicrons and Extremely Large VLDL | XXL_VLDL_TG | Lipoprotein |
| Concentration of Very Large VLDL Particles | XL_VLDL_P | Lipoprotein |
| Total Lipids in Very Large VLDL | XL_VLDL_L | Lipoprotein |
| Cholesterol in Very Large VLDL | XL_VLDL_C | Lipoprotein |
| Cholesteryl Esters in Very Large VLDL | XL_VLDL_CE | Lipoprotein |
| Free Cholesterol in Very Large VLDL | XL_VLDL_FC | Lipoprotein |
| Triglycerides in Very Large VLDL | XL_VLDL_TG | Lipoprotein |
| Concentration of Large VLDL Particles | L_VLDL_P | Lipoprotein |
| Total Lipids in Large VLDL | L_VLDL_L | Lipoprotein |
| Cholesterol in Large VLDL | L_VLDL_C | Lipoprotein |
| Cholesteryl Esters in Large VLDL | L_VLDL_CE | Lipoprotein |
| Free Cholesterol in Large VLDL | L_VLDL_FC | Lipoprotein |
| Triglycerides in Large VLDL | L_VLDL_TG | Lipoprotein |
| Concentration of Medium VLDL Particles | M_VLDL_P | Lipoprotein |
| Total Lipids in Medium VLDL | M_VLDL_L | Lipoprotein |
| Cholesterol in Medium VLDL | M_VLDL_C | Lipoprotein |
| Cholesteryl Esters in Medium VLDL | M_VLDL_CE | Lipoprotein |
| Free Cholesterol in Medium VLDL | M_VLDL_FC | Lipoprotein |
| Triglycerides in Medium VLDL | M_VLDL_TG | Lipoprotein |
| Concentration of Small VLDL Particles | S_VLDL_P | Lipoprotein |
| Total Lipids in Small VLDL | S_VLDL_L | Lipoprotein |
| Cholesterol in Small VLDL | S_VLDL_C | Lipoprotein |
| Cholesteryl Esters in Small VLDL | S_VLDL_CE | Lipoprotein |
| Free Cholesterol in Small VLDL | S_VLDL_FC | Lipoprotein |
| Triglycerides in Small VLDL | S_VLDL_TG | Lipoprotein |
| Concentration of Very Small VLDL Particles | XS_VLDL_P | Lipoprotein |
| Total Lipids in Very Small VLDL | XS_VLDL_L | Lipoprotein |
| Cholesterol in Very Small VLDL | XS_VLDL_C | Lipoprotein |
| Cholesteryl Esters in Very Small VLDL | XS_VLDL_CE | Lipoprotein |
| Free Cholesterol in Very Small VLDL | XS_VLDL_FC | Lipoprotein |
| Triglycerides in Very Small VLDL | XS_VLDL_TG | Lipoprotein |
| Concentration of IDL Particles | IDL_P | Lipoprotein |
| Total Lipids in IDL | IDL_L | Lipoprotein |
| Cholesterol in IDL | IDL_C | Lipoprotein |
| Cholesteryl Esters in IDL | IDL_CE | Lipoprotein |
| Free Cholesterol in IDL | IDL_FC | Lipoprotein |
| Triglycerides in IDL | IDL_TG | Lipoprotein |
| Concentration of Large LDL Particles | L_LDL_P | Lipoprotein |
| Total Lipids in Large LDL | L_LDL_L | Lipoprotein |
| Cholesterol in Large LDL | L_LDL_C | Lipoprotein |
| Cholesteryl Esters in Large LDL | L_LDL_CE | Lipoprotein |
| Free Cholesterol in Large LDL | L_LDL_FC | Lipoprotein |
| Triglycerides in Large LDL | L_LDL_TG | Lipoprotein |
| Concentration of Medium LDL Particles | M_LDL_P | Lipoprotein |
| Total Lipids in Medium LDL | M_LDL_L | Lipoprotein |
| Cholesterol in Medium LDL | M_LDL_C | Lipoprotein |
| Cholesteryl Esters in Medium LDL | M_LDL_CE | Lipoprotein |
| Free Cholesterol in Medium LDL | M_LDL_FC | Lipoprotein |
| Triglycerides in Medium LDL | M_LDL_TG | Lipoprotein |
| Concentration of Small LDL Particles | S_LDL_P | Lipoprotein |
| Total Lipids in Small LDL | S_LDL_L | Lipoprotein |
| Cholesterol in Small LDL | S_LDL_C | Lipoprotein |
| Cholesteryl Esters in Small LDL | S_LDL_CE | Lipoprotein |
| Free Cholesterol in Small LDL | S_LDL_FC | Lipoprotein |
| Triglycerides in Small LDL | S_LDL_TG | Lipoprotein |
| Concentration of Very Large HDL Particles | XL_HDL_P | Lipoprotein |
| Total Lipids in Very Large HDL | XL_HDL_L | Lipoprotein |
| Cholesterol in Very Large HDL | XL_HDL_C | Lipoprotein |
| Cholesteryl Esters in Very Large HDL | XL_HDL_CE | Lipoprotein |
| Free Cholesterol in Very Large HDL | XL_HDL_FC | Lipoprotein |
| Triglycerides in Very Large HDL | XL_HDL_TG | Lipoprotein |
| Concentration of Large HDL Particles | L_HDL_P | Lipoprotein |
| Total Lipids in Large HDL | L_HDL_L | Lipoprotein |
| Cholesterol in Large HDL | L_HDL_C | Lipoprotein |
| Cholesteryl Esters in Large HDL | L_HDL_CE | Lipoprotein |
| Free Cholesterol in Large HDL | L_HDL_FC | Lipoprotein |
| Triglycerides in Large HDL | L_HDL_TG | Lipoprotein |
| Concentration of Medium HDL Particles | M_HDL_P | Lipoprotein |
| Total Lipids in Medium HDL | M_HDL_L | Lipoprotein |
| Cholesterol in Medium HDL | M_HDL_C | Lipoprotein |
| Cholesteryl Esters in Medium HDL | M_HDL_CE | Lipoprotein |
| Free Cholesterol in Medium HDL | M_HDL_FC | Lipoprotein |
| Triglycerides in Medium HDL | M_HDL_TG | Lipoprotein |
| Concentration of Small HDL Particles | S_HDL_P | Lipoprotein |
| Total Lipids in Small HDL | S_HDL_L | Lipoprotein |
| Cholesterol in Small HDL | S_HDL_C | Lipoprotein |
| Cholesteryl Esters in Small HDL | S_HDL_CE | Lipoprotein |
| Free Cholesterol in Small HDL | S_HDL_FC | Lipoprotein |
| Triglycerides in Small HDL | S_HDL_TG | Lipoprotein |
| Cholesterol to Total Lipids in Chylomicrons and Extremely Large VLDL percentage | XXL_VLDL_C_pct | Lipoprotein |
| Cholesteryl Esters to Total Lipids in Chylomicrons and Extremely Large VLDL percentage | XXL_VLDL_CE_pct | Lipoprotein |
| Free Cholesterol to Total Lipids in Chylomicrons and Extremely Large VLDL percentage | XXL_VLDL_FC_pct | Lipoprotein |
| Triglycerides to Total Lipids in Chylomicrons and Extremely Large VLDL percentage | XXL_VLDL_TG_pct | Lipoprotein |
| Cholesterol to Total Lipids in Very Large VLDL percentage | XL_VLDL_C_pct | Lipoprotein |
| Cholesteryl Esters to Total Lipids in Very Large VLDL percentage | XL_VLDL_CE_pct | Lipoprotein |
| Free Cholesterol to Total Lipids in Very Large VLDL percentage | XL_VLDL_FC_pct | Lipoprotein |
| Triglycerides to Total Lipids in Very Large VLDL percentage | XL_VLDL_TG_pct | Lipoprotein |
| Cholesterol to Total Lipids in Large VLDL percentage | L_VLDL_C_pct | Lipoprotein |
| Cholesteryl Esters to Total Lipids in Large VLDL percentage | L_VLDL_CE_pct | Lipoprotein |
| Free Cholesterol to Total Lipids in Large VLDL percentage | L_VLDL_FC_pct | Lipoprotein |
| Triglycerides to Total Lipids in Large VLDL percentage | L_VLDL_TG_pct | Lipoprotein |
| Cholesterol to Total Lipids in Medium VLDL percentage | M_VLDL_C_pct | Lipoprotein |
| Cholesteryl Esters to Total Lipids in Medium VLDL percentage | M_VLDL_CE_pct | Lipoprotein |
| Free Cholesterol to Total Lipids in Medium VLDL percentage | M_VLDL_FC_pct | Lipoprotein |
| Triglycerides to Total Lipids in Medium VLDL percentage | M_VLDL_TG_pct | Lipoprotein |
| Cholesterol to Total Lipids in Small VLDL percentage | S_VLDL_C_pct | Lipoprotein |
| Cholesteryl Esters to Total Lipids in Small VLDL percentage | S_VLDL_CE_pct | Lipoprotein |
| Free Cholesterol to Total Lipids in Small VLDL percentage | S_VLDL_FC_pct | Lipoprotein |
| Triglycerides to Total Lipids in Small VLDL percentage | S_VLDL_TG_pct | Lipoprotein |
| Cholesterol to Total Lipids in Very Small VLDL percentage | XS_VLDL_C_pct | Lipoprotein |
| Cholesteryl Esters to Total Lipids in Very Small VLDL percentage | XS_VLDL_CE_pct | Lipoprotein |
| Free Cholesterol to Total Lipids in Very Small VLDL percentage | XS_VLDL_FC_pct | Lipoprotein |
| Triglycerides to Total Lipids in Very Small VLDL percentage | XS_VLDL_TG_pct | Lipoprotein |
| Cholesterol to Total Lipids in IDL percentage | IDL_C_pct | Lipoprotein |
| Cholesteryl Esters to Total Lipids in IDL percentage | IDL_CE_pct | Lipoprotein |
| Free Cholesterol to Total Lipids in IDL percentage | IDL_FC_pct | Lipoprotein |
| Triglycerides to Total Lipids in IDL percentage | IDL_TG_pct | Lipoprotein |
| Cholesterol to Total Lipids in Large LDL percentage | L_LDL_C_pct | Lipoprotein |
| Cholesteryl Esters to Total Lipids in Large LDL percentage | L_LDL_CE_pct | Lipoprotein |
| Free Cholesterol to Total Lipids in Large LDL percentage | L_LDL_FC_pct | Lipoprotein |
| Triglycerides to Total Lipids in Large LDL percentage | L_LDL_TG_pct | Lipoprotein |
| Cholesterol to Total Lipids in Medium LDL percentage | M_LDL_C_pct | Lipoprotein |
| Cholesteryl Esters to Total Lipids in Medium LDL percentage | M_LDL_CE_pct | Lipoprotein |
| Free Cholesterol to Total Lipids in Medium LDL percentage | M_LDL_FC_pct | Lipoprotein |
| Triglycerides to Total Lipids in Medium LDL percentage | M_LDL_TG_pct | Lipoprotein |
| Cholesterol to Total Lipids in Small LDL percentage | S_LDL_C_pct | Lipoprotein |
| Cholesteryl Esters to Total Lipids in Small LDL percentage | S_LDL_CE_pct | Lipoprotein |
| Free Cholesterol to Total Lipids in Small LDL percentage | S_LDL_FC_pct | Lipoprotein |
| Triglycerides to Total Lipids in Small LDL percentage | S_LDL_TG_pct | Lipoprotein |
| Cholesterol to Total Lipids in Very Large HDL percentage | XL_HDL_C_pct | Lipoprotein |
| Cholesteryl Esters to Total Lipids in Very Large HDL percentage | XL_HDL_CE_pct | Lipoprotein |
| Free Cholesterol to Total Lipids in Very Large HDL percentage | XL_HDL_FC_pct | Lipoprotein |
| Triglycerides to Total Lipids in Very Large HDL percentage | XL_HDL_TG_pct | Lipoprotein |
| Cholesterol to Total Lipids in Large HDL percentage | L_HDL_C_pct | Lipoprotein |
| Cholesteryl Esters to Total Lipids in Large HDL percentage | L_HDL_CE_pct | Lipoprotein |
| Free Cholesterol to Total Lipids in Large HDL percentage | L_HDL_FC_pct | Lipoprotein |
| Triglycerides to Total Lipids in Large HDL percentage | L_HDL_TG_pct | Lipoprotein |
| Cholesterol to Total Lipids in Medium HDL percentage | M_HDL_C_pct | Lipoprotein |
| Cholesteryl Esters to Total Lipids in Medium HDL percentage | M_HDL_CE_pct | Lipoprotein |
| Free Cholesterol to Total Lipids in Medium HDL percentage | M_HDL_FC_pct | Lipoprotein |
| Triglycerides to Total Lipids in Medium HDL percentage | M_HDL_TG_pct | Lipoprotein |
| Cholesterol to Total Lipids in Small HDL percentage | S_HDL_C_pct | Lipoprotein |
| Cholesteryl Esters to Total Lipids in Small HDL percentage | S_HDL_CE_pct | Lipoprotein |
| Free Cholesterol to Total Lipids in Small HDL percentage | S_HDL_FC_pct | Lipoprotein |
| Triglycerides to Total Lipids in Small HDL percentage | S_HDL_TG_pct | Lipoprotein |
| Total Phospholipids in Lipoprotein Particles | Total_PL | Phospholipids |
| Phospholipids in VLDL | VLDL_PL | Phospholipids |
| Phospholipids in LDL | LDL_PL | Phospholipids |
| Phospholipids in HDL | HDL_PL | Phospholipids |
| Phosphoglycerides | Phosphoglyc | Phospholipids |
| Phosphatidylcholines | Phosphatidylc | Phospholipids |
| Sphingomyelins | Sphingomyelins | Phospholipids |
| Phospholipids in Chylomicrons and Extremely Large VLDL | XXL_VLDL_PL | Phospholipids |
| Phospholipids in Very Large VLDL | XL_VLDL_PL | Phospholipids |
| Phospholipids in Large VLDL | L_VLDL_PL | Phospholipids |
| Phospholipids in Medium VLDL | M_VLDL_PL | Phospholipids |
| Phospholipids in Small VLDL | S_VLDL_PL | Phospholipids |
| Phospholipids in Very Small VLDL | XS_VLDL_PL | Phospholipids |
| Phospholipids in IDL | IDL_PL | Phospholipids |
| Phospholipids in Large LDL | L_LDL_PL | Phospholipids |
| Phospholipids in Medium LDL | M_LDL_PL | Phospholipids |
| Phospholipids in Small LDL | S_LDL_PL | Phospholipids |
| Phospholipids in Very Large HDL | XL_HDL_PL | Phospholipids |
| Phospholipids in Large HDL | L_HDL_PL | Phospholipids |
| Phospholipids in Medium HDL | M_HDL_PL | Phospholipids |
| Phospholipids in Small HDL | S_HDL_PL | Phospholipids |
| Phospholipids to Total Lipids in Chylomicrons and Extremely Large VLDL percentage | XXL_VLDL_PL_pct | Phospholipids |
| Phospholipids to Total Lipids in Very Large VLDL percentage | XL_VLDL_PL_pct | Phospholipids |
| Phospholipids to Total Lipids in Large VLDL percentage | L_VLDL_PL_pct | Phospholipids |
| Phospholipids to Total Lipids in Medium VLDL percentage | M_VLDL_PL_pct | Phospholipids |
| Phospholipids to Total Lipids in Small VLDL percentage | S_VLDL_PL_pct | Phospholipids |
| Phospholipids to Total Lipids in Very Small VLDL percentage | XS_VLDL_PL_pct | Phospholipids |
| Phospholipids to Total Lipids in IDL percentage | IDL_PL_pct | Phospholipids |
| Phospholipids to Total Lipids in Large LDL percentage | L_LDL_PL_pct | Phospholipids |
| Phospholipids to Total Lipids in Medium LDL percentage | M_LDL_PL_pct | Phospholipids |
| Phospholipids to Total Lipids in Small LDL percentage | S_LDL_PL_pct | Phospholipids |
| Phospholipids to Total Lipids in Very Large HDL percentage | XL_HDL_PL_pct | Phospholipids |
| Phospholipids to Total Lipids in Large HDL percentage | L_HDL_PL_pct | Phospholipids |
| Phospholipids to Total Lipids in Medium HDL percentage | M_HDL_PL_pct | Phospholipids |
| Phospholipids to Total Lipids in Small HDL percentage | S_HDL_PL_pct | Phospholipids |
| Total Cholines | Cholines | Proteins |
| Apolipoprotein B | ApoB | Proteins |
| Apolipoprotein A1 | ApoA1 | Proteins |
| Apolipoprotein B to Apolipoprotein A1 ratio | ApoB_by_ApoA1 | Proteins |
| Albumin | Albumin | Proteins |
| Glycoprotein Acetyls | GlycA | Proteins |
| 3-Hydroxybutyrate | bOHbutyrate | Others |
| Acetate | Acetate | Others |
| Acetoacetate | Acetoacetate | Others |
| Acetone | Acetone | Others |
| Creatinine | Creatinine | Others |

# **Scoring Criteria for Dietary Patterns**

1. **Components and details of the Alternative Healthy Eating Index (AHEI-2010) based on previous literature ^1^**

| **Food component** | **Contributing foods from the Oxford WebQ** | **Unit** | **Component score** | |
| --- | --- | --- | --- | --- |
|  |  |  | Min (0) | Max (10) |
| Vegetables | Mixed vegetables, vegetable pieces, coleslaw, side salad, avocado, green beans, beetroot, broccoli, butternut squash, cabbage/kale, carrot, cauliflower, celery, courgette, cucumber, garlic, leek, lettuce, mushroom, onion, parsnip, peas, sweet pepper, spinach, sprouts, sweetcorn, sweet potato, fresh tomato, tin tomato, turnip/swede, watercress, other vegetable, vegetables from canned or homemade soup, guacamole-based sauce, tomato-based sauce | Serving/day | 0 | ≥5 |
| Fruit (not fruit juice) | Stewed fruit, prune, dried fruit, mixed fruit, apple, banana, berry, cherry, grapefruit, grape, mango, melon, orange, satsuma, peach/nectarine, pear, pineapple, plum, other fruit, olives | Serving/day | 0 | ≥4 |
| Red and processed meat | Beef, pork, lamb, red meat from canned or homemade soup, bacon, ham, sausage, liver | Serving/day | ≥1.5 | 0 |
| Nuts and legumes | Unsalted nuts, salted nuts, unsalted peanuts, salted peanuts, peanut butter-based sauce, seeds, broad beans, baked beans, pulses, tofu, hummus-based sauce, pulses from canned or homemade soup | Serving/day | 0 | ≥1 |
| Sugary drinks and fruit juices | Fizzy drink, squash, fruit smoothie, flavored milk, hot chocolate, orange juice, grapefruit juice, pure fruit/vegetable juice | Serving/day | ≥1 | 0 |
| Whole grains | Bran cereal, oat cereal, whole-meal bread cereal/breads, whole-meal pasta, brown rice and other wholegrain, muesli (1/2 serving) | g/d (weight) | 0 | 75 (Female) |
|  |  |  |  | 90 (Male) |
| Trans fat^†^ | Trans fatty acids (g/d), energy (KJ/d) | % energy | ≥4 | ≤0.5 |
| Long chain (n-3) fats | Oily fish | Serving/week | 0 | ≥2 |
| Polyunsaturated fatty acids (PUFA)^†^ | n-3 fatty acids (g/d), n-6 fatty acids (g/d), energy (KJ/d) | % energy | ≤2 | ≥10 |
| Sodium | Sodium | mg/d | Highest decile | Lowest decile |
| Alcohol | Beer and cider(Beer/cider intake), fortified wine, red wine, white wine, spirits | drinks/day^‡^ | >=2.5 (Female)/>=3.5 (Male) | 0.5-1.5 (Female)/0.5-2.0 (Male) |

Each category is scored from 0 to 10, with a total score ranging from 0 to 110. The scores for the other dietary patterns were standardized using the energy residual method, adjusting total energy intake to a uniform baseline of 2000 kcal to eliminate confounding effects of total energy intake on dietary quality scores

1. **Components and details of the Dietary Approaches to Stop Hypertension (DASH) adherence score based on previous literature^1^**

| **Food component** | **Contributing foods from the Oxford WebQ** | **Unit** | **Component score** | |
| --- | --- | --- | --- | --- |
|  |  |  | Min (1) | Max (5) |
| Vegetables | Mixed vegetables, vegetable pieces, coleslaw, side salad, avocado, green beans, beetroot, broccoli, butternut squash, cabbage/kale, carrot, cauliflower, celery, courgetti, cucumber, garlic, leek, lettuce, mushroom, onion, parsnip, peas, sweet pepper, spinach, sprouts, sweetcorn, sweet potato, fresh tomato, tin tomato, turnip/swede, watercress, other vegetable, vegetables from canned or homemade soup, guacamole-based sauce, tomato-based sauce | Serving/day | Q1 | Q5 |
| Fruit (including fruit juice) | Stewed fruit, prune, dried fruit, mixed fruit, apple, banana, berry, cherry, grapefruit, grape, mango, melon, orange, satsuma, peach/nectarine, pear, pineapple, plum, other fruit, olives, grapefruit juice, orange juice, pure fruit/vegetable juice | Serving/day | Q1 | Q5 |
| Red and processed meat | Beef, pork, lamb, red meat from canned or homemade soup, bacon, ham, sausage, liver | Serving/day | Q5 | Q1 |
| Nuts and legumes | Unsalted nuts, salted nuts, unsalted peanuts, salted peanuts, peanut butter-based sauce, seeds, broad beans, baked beans, pulses, tofu, hummus-based sauce, pulses from canned or homemade soup | Serving/day | Q1 | Q5 |
| Whole grains | Porridge, muesli, oat crunch, bran cereal, whole-wheat cereal/bread, sliced bread (type of sliced bread eaten: non-white), baguette (type of baguette eaten: non-white), bap (type of large bap eaten: non-white), bread roll (type of bread roll eaten: non-white), crispbread, oatcakes, whole-meal pasta, brown rice, couscous, other grain | Serving/day | Q1 | Q5 |
| Low-fat dairy | Low fat cheese spread, low fat hard cheese, cottage cheese, skimmed milk, semi-skimmed milk, low fat yogurt | Serving/day | Q1 | Q5 |
| Sugary drinks | Fizzy drinks, low calories drinks, squash | Serving/day | Q5 | Q1 |
| Sodium | Sodium | mg/d | Q5 | Q1 |

Participants were divided into five quintiles by dietary component intakes for the DASH diet, and different scores were assigned to Q1 through Q5. Each component is scored from 0 to 5 points, with a total score range of 0 to 40. The scores for the other dietary patterns were standardized using the energy residual method, adjusting total energy intake to a uniform baseline of 2000 kcal to eliminate confounding effects of total energy intake on dietary quality scores

1. **Components and details of the Energy‑adjusted Diet Inflammatory Index (E‑DII) score based on previous literature^1, 2^**

| **Components** | **Overall inflammatory effect score** | **Global daily mean intake (units/d)** | **SD** |
| --- | --- | --- | --- |
| Alcohol (g) | –0.278 | 13.98 | 3.72 |
| Vitamin B12 (μg) | 0.106 | 5.15 | 2.7 |
| Vitamin B6 (mg) | –0.365 | 1.47 | 0.74 |
| β-Carotene (μg) | –0.584 | 3718 | 1720 |
| Carbohydrate (g) | 0.097 | 272.2 | 40 |
| Cholesterol (mg) | 0.11 | 279·4 | 51.2 |
| Energy (kcal) | 0.18 | 2056 | 338 |
| Total fat (g) | 0.298 | 71.4 | 19.4 |
| Fiber (g) | –0.663 | 18.8 | 4.9 |
| Folate (μg) | –0.190 | 273 | 70.7 |
| Garlic (g) | –0.412 | 4.35 | 2.9 |
| Iron (mg) | 0.032 | 13.35 | 3.71 |
| Magnesium (mg) | –0.484 | 310.1 | 139.4 |
| Monounsaturated fatty acids (g) | –0.009 | 27 | 6.1 |
| Niacin (mg) | –0.246 | 25.9 | 11.77 |
| n-3 Fatty acids (g) | –0.436 | 1.06 | 1.06 |
| n-6 Fatty acids (g) | –0.159 | 10.8 | 7.5 |
| Onion (g) | –0.301 | 35.9 | 18.4 |
| Protein (g) | 0.021 | 79.4 | 13.9 |
| Polyunsaturated fatty acid (g) | –0.337 | 13.88 | 3.76 |
| Riboflavin (mg) | –0.068 | 1.7 | 0.79 |
| Saturated fat (g) | 0.373 | 28.6 | 8 |
| Se (μg) | –0.191 | 67 | 25.1 |
| Thiamin (mg) | –0.098 | 1.7 | 0.66 |
| Trans fat (g) | 0.229 | 3.15 | 3.75 |
| Vitamin A (retinol equivalents) | –0.401 | 983.9 | 518.6 |
| Vitamin C (mg) | –0.424 | 118.2 | 43.46 |
| Vitamin D (μg) | –0.446 | 6.26 | 2.21 |
| Vitamin E (mg) | –0.419 | 8.73 | 1.49 |
| Zn (mg) | –0.313 | 9.84 | 2.19 |
| Green tea (g) | –0.536 | 1.69 | 1.53 |
| Sweet Pepper (g) | –0.131 | 10 | 7.07 |

Dietary components standardized to intake per 1000 Kcal of energy

1. **Components and details of the Mediterranean Diet (MED) score based on previous literature ^3, 4^**

| Food component | Contributing foods from the Oxford WebQ | **Unit** | **Component score** | |
| --- | --- | --- | --- | --- |
|  |  |  | **0** | **1** |
| Olive oil | Type of fat/ oil used for cooking | - | Non-consumption | Consumption |
| Vegetables | Carrot, spinach, broccoli, cabbage/ kale, sprouts, courgette, cauliflower, parsnip, turnip/ swede, leek, onion, garlic, mushroom, sweet pepper, side salad, lettuce, cucumber, celery, watercress, fresh tomato, tin tomato, sweetcorn, beetroot, avocado, mixed vegetables, vegetable pieces, butternut squash, other vegetables, olives, coleslaw, guacamole, vegetables from canned soup, vegetables from homemade soup | Serving/day | <2 (and/or not including 1/d raw or salad) | ≥2 (including ≥1 raw or salad) |
| Fruit | Apple, pear, orange, satsuma, grapefruit, banana, grape, melon, peach/ nectarine, plum, berry, dried fruit, stewed fruit, mixed fruit, prune, cherry, mango, pineapple, other fruit, fruit added to cereal, grapefruit juice, orange juice | Serving/day | <3 | ≥3 |
| Red meat | Beef, pork, lamb, red meat from canned soup, red meat from homemade soup, bacon, ham , sausage, liver, meat from Scotch egg | Serving/day | >1 | <1 |
| Butter, margarine or cream | Butter/ margarine on potato, baguettes with butter/ margarine, baps with butter/ margarine, bread rolls with butter/ margarine, bread slices with butter/ margarine, crackers/ crispbread with butter/ margarine, oatcakes with butter/ margarine, other bread with butter/ margarine, butter/ margarine used in cooking, cream | Serving/day | >1 | <1 |
| Sweetened or carbonated drinks | Fizzy drinks, low calories drinks, squash intake | Serving/day | >1 | <1 |
| Wine | Red wine, rose wine, white wine | drinks/day | <7 | ≥7 |
| Legumes | Peas, green beans, broad beans, baked beans, pulses, tofu, hummus, pulses from canned soup, pulses from homemade soup | Serving/day | <3 | ≥3 |
| Seafood | Battered fish, breaded fish, white fish, oily fish, shellfish, other fish, tinned tuna, prawn, lobster/ crab, fish from canned soup, fish from homemade soup | Serving/day | <3 | ≥3 |
| Sweets or pastries | Chocolate biscuit, chocolate covered biscuit, chocolate bar, chocolate sweets, chocolate raisins, dark chocolate, milk chocolate, white chocolate, sweet biscuits, cakes, cheesecake, doughnut, fruitcake, Danish pastry, sponge pudding, milk-based pudding, other milk-based pudding, other desert intake, soya desert intake, sweets, diet sweets, other sweets, ice cream | Serving/day | >2 | <2 |
| Nuts | Unsalted nuts, salted nuts, unsalted peanuts, salted peanuts, peanut butter | Serving/day | <3 | ≥3 |
| White meat | Poultry, breaded poultry, white meat from canned soup, white meat from homemade soup | - | Less white meat than red meat | More white meat than red meat |
| Sofrito | Tomato-based sauce | Serving/day | <2 | ≥2 |

Each category is scored from 0 to 1, with a total score ranging from 0 to 13. The scores for the other dietary patterns were standardized using the energy residual method, adjusting total energy intake to a uniform baseline of 2000 kcal to eliminate confounding effects of total energy intake on dietary quality scores

1. **Components and details of the Mediterranean-DASH Intervention for Neurodegenerative Delay (MIND) diet score based on previous literature^1, 5^**

| **Food component** | **Contributing foods from the Oxford WebQ** | **Unit** | **Component score** | | |
| --- | --- | --- | --- | --- | --- |
|  |  |  | 0 | 0.5 | 1 |
| Green leafy vegetables | Coleslaw, side salad (1/2 serving), cabbage/kale, lettuce, spinach, watercress | Serving/week | ≤2 | 2-6 | ≥6 |
| Other vegetables | Mixed vegetables, vegetable pieces, side salad (1/2 serving), avocado, green beans, beetroot, broccoli, butternut squash, carrot, cauliflower, celery, courgette, cucumber, garlic, leek, lettuce, mushroom, onion, parsnip, peas, sweet pepper, sprouts, sweetcorn, sweet potato, fresh tomato, tin tomato, turnip/swede, other vegetable, vegetables from canned or homemade soup, guacamole-based sauce, tomato-based sauce | Serving/week | <5 | 5-7 | ≥7 |
| Berries | Berry | Serving/week | <1 | 1-2 | ≥2 |
| Olive oil | Type of fat/ oil used for cooking | - | No | - | Yes |
| Red and processed meat | Beef, pork, lamb, red meat from canned or homemade soup, bacon, ham, sausage, liver | Serving/week | >6 | 4-6 | <4 |
| Poultry | Poultry intake, poultry from canned soup or homemade soup | Serving/week | <1 | 1-2 | >2 |
| Butter and margarine | Butter/ margarine on potato, baguettes with butter/ margarine, baps with butter/ margarine, bread rolls with butter/ margarine, bread slices with butter/ margarine, crackers/ crispbread with butter/ margarine, oatcakes with butter/ margarine, other bread with butter/ margarine, butter/ margarine used in cooking, cream | Tsp/day (1 tsp=15g) | <1 | 1-2 | >2 |
| Cheese (not low fat) | Hard cheese, soft cheese, blue cheese, cheese spread, feta, mozzarella, goat’s cheese, other cheese (not use low-fat version) | Serving/week | ≥6 | 1-6 | <1 |
| Wine | Red wine, rose wine, white wine, fortified wine | Serving/week | <0.25 or >7 | 0.25-1 | >1 |
| Legumes | Broad beans, baked beans, pulses, tofu, hummus-based sauce, pulses from canned or homemade soup | Serving/week | <1 | 1-3 | >3 |
| Nuts | Unsalted nuts, salted nuts, unsalted peanuts, salted peanuts, peanut butter-based sauce, seeds | Serving/week | <0.5 | 0.5-4 | >4 |
| Seafood (not fried) | White fish, oily fish, shellfish, other fish, tinned tuna, prawn, lobster/crab, fish from canned or homemade soup | Serving/week | <0.25 | 0.25-1 | ≥1 |
| Sweets, desserts, and sugary drinks | Chocolate biscuit, chocolate covered biscuit, chocolate bar, chocolate sweets, chocolate raisins, dark chocolate, milk chocolate, white chocolate, sweet biscuits, sweets, diet sweets, other sweets, cakes, cheesecake, doughnut, fruitcake, Danish pastry, sponge pudding, milk-based pudding, other milk-based pudding, other desert, soya desert, ice cream, cereal bar, double crust pastry, single crust pastry, crumble, pancake, scotch pancake, Yorkshire pudding, croissant, scone, flavored milk, hot chocolate, fizzy drink, squash | Serving/week | ≥7 | 5-7 | <5 |
| Whole grain | Porridge, muesli, oat crunch, bran cereal, whole-wheat cereal/bread, sliced bread (type of sliced bread eaten: non-white), baguette (type of baguette eaten: non-white), bap (type of large bap eaten: non-white), bread roll (type of bread roll eaten: non-white), crispbread, oatcakes, whole-meal pasta, brown rice, couscous, other grain | Serving/day | <1 | 1-2 | >2 |
| Fast/fried foods | Crumbed or deep-fried poultry, breaded fish, battered fish, fried potatoes, crisp/chips | Serving/week | >3 | 1-3 | <1 |

The scores for the other dietary patterns were standardized using the energy residual method, adjusting total energy intake to a uniform baseline of 2000 kcal to eliminate confounding effects of total energy intake on dietary quality scores

**References**

1. Zhu K, Li R, Yao P, Yu H, Pan A, Manson JE, et al. Proteomic signatures of healthy dietary patterns are associated with lower risks of major chronic diseases and mortality. Nature Food. 2025;6(1):47-57. doi:<http://dx.doi.org/10.1038/s43016-024-01059-x>.

2. Cavicchia PP, Steck SE, Hurley TG, Hussey JR, Ma Y, Ockene IS, et al. A new dietary inflammatory index predicts interval changes in serum high-sensitivity C-reactive protein. The Journal of nutrition. 2009;139(12):2365-72. doi:<http://dx.doi.org/10.3945/jn.109.114025>.

3. Martínez-González MÁ, Corella D, Salas-Salvadó J, Ros E, Covas MI, Fiol M, et al. Cohort Profile: Design and methods of the PREDIMED study. International Journal of Epidemiology. 2012;41(2):377-85. doi:<http://dx.doi.org/10.1093/ije/dyq250>.

4. Shannon OM, Ranson JM, Gregory S, Macpherson H, Milte C, Lentjes M, et al. Mediterranean diet adherence is associated with lower dementia risk, independent of genetic predisposition: findings from the UK Biobank prospective cohort study. BMC medicine. 2023;21(1):81. doi:<http://dx.doi.org/10.1186/s12916-023-02772-3>.

5. Morris MC, Tangney CC, Wang Y, Sacks FM, Barnes LL, Bennett DA, et al. MIND diet slows cognitive decline with aging. Alzheimer's & Dementia. 2015;11(9):1015-22. doi:<http://dx.doi.org/https://doi.org/10.1016/j.jalz.2015.04.011>.
